# Serum- and glucocorticoid-induced kinase 3 orchestrates glucocorticoid signaling to facilitate chromatin remodeling during murine adipogenesis

**DOI:** 10.1172/JCI186534

**Published:** 2025-07-24

**Authors:** Qilong Chen, Jialu Guo, Yuyi Liu, Du, Jiapei Liu, Yuyao Zhang, Yuming Dai, Mengdi Zhang, Ziqian Zhou, Qiyang Zhang, Caixia Wei, Qiurong Ding, Jun Qin, Qiwei Zhai, Ju Qiu, Mengle Shao, Fang Zhang, Alexander A. Soukas, Ben Zhou

**Affiliations:** 1 Shanghai Institute of Nutrition and Health, University of Chinese Academy of Sciences, Chinese Academy of Sciences, Shanghai, China.; 2 National Clinical Research Center for Eye Diseases, Department of Ophthalmology, Shanghai General Hospital, Shanghai Jiao Tong University School of Medicine, Shanghai, China.; 3 Department of Medicine, Diabetes Unit and Center for Genomic Medicine, Massachusetts General Hospital and Harvard Medical School, Boston, Massachusetts, USA.; 4 State Key Laboratory of Immune Response and Immunotherapy, Shanghai Institute of Materia Medica, Chinese Academy of Sciences, Shanghai, China.

**Keywords:** Cell biology, Metabolism, Adipose tissue, Obesity

## Abstract

Elevated glucocorticoid levels are common in conditions such as aging, chronic stress, Cushing syndrome, and glucocorticoid therapy. While glucocorticoids suppress inflammation through the glucocorticoid receptor (GR), they also cause metabolic side effects. Investigating alternative pathways beyond GR activation is crucial for reducing these side effects. Our phosphoproteomics analysis revealed that glucocorticoid exposure promotes phosphorylation at the RxxS motifs of multiple proteins in preadipocytes, including those mediated by serum- and glucocorticoid-induced kinase 3 (SGK3). SGK3 is a key mediator of glucocorticoid-induced adipogenesis, as shown by impaired adipogenesis after SGK3 inhibition or genetic ablation. *Sgk3*-KO mice were resistant to obesity induced by glucocorticoid or a high-fat diet, and proteolysis targeting chimeras (PROTAC) targeting SGK3 reduced adipogenesis in both obese mice and in a thyroid eye disease cell line. Mechanistically, SGK3 translocated to the nucleus upon glucocorticoid stimulation, interacted with and phosphorylated the BRG1 subunit of the BAF complex, and prevented BRG1 degradation, promoting chromatin remodeling necessary for adipogenesis. These findings highlight SGK3 as a potential therapeutic target to mitigate metabolic side effects of elevated glucocorticoid levels.

## Abstract 2

Glucocorticoids promotes adipogenesis through SGK3-mediated chromatin remodeling independent of GR, and downregulation of SGK3 inhibits glucocorticoid or high fat diet induced adipogenesis in mice.

## Introduction

An elevated glucocorticoid level is a common feature in pathophysiological states, including aging, chronic stress, Cushing syndrome, and glucocorticoid therapy. Glucocorticoids are commonly prescribed for managing inflammation in a spectrum of autoimmune, inflammatory, and allergic conditions such as thyroid eye disease, rheumatoid arthritis, lupus erythematosus, inflammatory bowel disease, transplant rejection, and asthma (1, 2). However, prolonged exposure to glucocorticoids leads to notable metabolic side effects, including central obesity, glucose intolerance, insulin resistance, and muscle mass loss (3), resembling conditions observed in individuals with excessive endogenous glucocorticoids, such as those with Cushing’s syndrome. The multifaceted role of glucocorticoids in human central obesity involves promoting adipogenesis and lipid accumulation in the visceral depot of white adipose tissue (WAT) while stimulating lipolysis in subcutaneous depots (4). Moreover, chronic glucocorticoid treatment contributes to increased lipid accumulation and suppressed thermogenesis in brown adipose tissue (5).

The effects of glucocorticoids are predominantly mediated through the glucocorticoid receptor (GR), a member of the nuclear hormone receptor family of transcription factors. Synthetic glucocorticoids like dexamethasone (DEX) are extensively used to induce adipogenesis. In vitro adipogenesis is usually initiated by treating postconfluent preadipocytes with an adipogenic cocktail consisting of rosiglitazone, isobutyl methylxanthine (IBMX), DEX, and insulin, collectively known as MDI. DEX rapidly binds and activates GR, leading to the expression of early adipogenic transcription factor (6). Activated GR also interacts with the histone acetyltransferase p300, promoting histone H3 acetylation and activation of enhancers for numerous adipogenic genes (7). However, recent studies have demonstrated that adipocyte GR is not required for the development of adipose tissue in mouse models (8–10). Moreover, glucocorticoids are reported to directly bind and activate certain G protein–coupled receptors such as GPR97 (11). Hence, other glucocorticoid-activated pathways independent of GR’s transcriptional activity might contribute to adipogenesis and warrant further exploration.

Adipogenesis involves orchestrated actions of transcription factors and epigenomic regulators at various differentiation stages (12, 13). Transcription factor binding to DNA often involves chromatin remodeling to enhance DNA accessibility. Mammalian SWI/SNF ATP-dependent chromatin remodeling complexes reorganize local nucleosome structures and modulate transcription factor access to their binding sites (14). SWI/SNF complexes are pivotal regulators of chromatin remodeling during adipogenesis (15–18), yet factors governing their activity remain elusive.

Mammalian serum- and glucocorticoid-inducible kinases (SGKs) comprise 3 isoforms: SGK1, SGK2, and SGK3. These AGC protein kinases, functioning parallel to AKT downstream of phosphatidylinositol 3-kinase (PI3K), possess conserved catalytic activation loop and C-terminal hydrophobic motif sites phosphorylated by PDK1 and mTORC2, respectively (19–21). SGK3, distinct from the other isoforms, is activated by both class 1 and class 3 PI3Ks (22). Its phox domain facilitates SGK3 targeting to endosomal compartments and vesicle-like structures, regulating endosome function (23, 24). Although SGK3 is known to regulate cell proliferation and survival (25, 26), its role in energy metabolism remains unclear. SGK3 is rapidly activated by glucocorticoid within minutes after treatment (27), suggesting its involvement in regulating pathways downstream of glucocorticoid independent of GR’s transcriptional activity.

Here, we found that glucocorticoid promptly induced phosphorylation of motifs featuring the RxxS pattern, partially mediated by SGK3. Using *Sgk3*-KO stromal vascular fraction (SVF) cells and a proteolysis targeting chimeras (PROTAC) targeting SGK3 degradation, we demonstrated that SGK3 promoted adipocyte differentiation. Glucocorticoid treatment elevated SGK3 protein levels in SVF cells, prompting SGK3 translocation to the nucleus. Nuclear SGK3 interacted with and stabilized the core SWI/SNF complex member SMARCA4 (BRG1), thereby facilitating adipogenesis via chromatin remodeling mediated by BRG1. *Sgk3*-KO mice exhibited protection from obesity induced by glucocorticoid or a high-fat diet (HFD), and SGK3 PROTAC also showed anti-adipogenesis effects on HFD-induced obesity, suggesting that inhibiting SGK3 could potentially ameliorate glucocorticoid-induced metabolic defects. Our study unveils that glucocorticoid induces adipogenesis via a pathway parallel to the GR pathway, offering a promising therapeutic target for alleviating glucocorticoid’s side effects.

## Results

### Glucocorticoids directly induce phosphoproteomic changes by activating SGK3.

**Figure 1:**
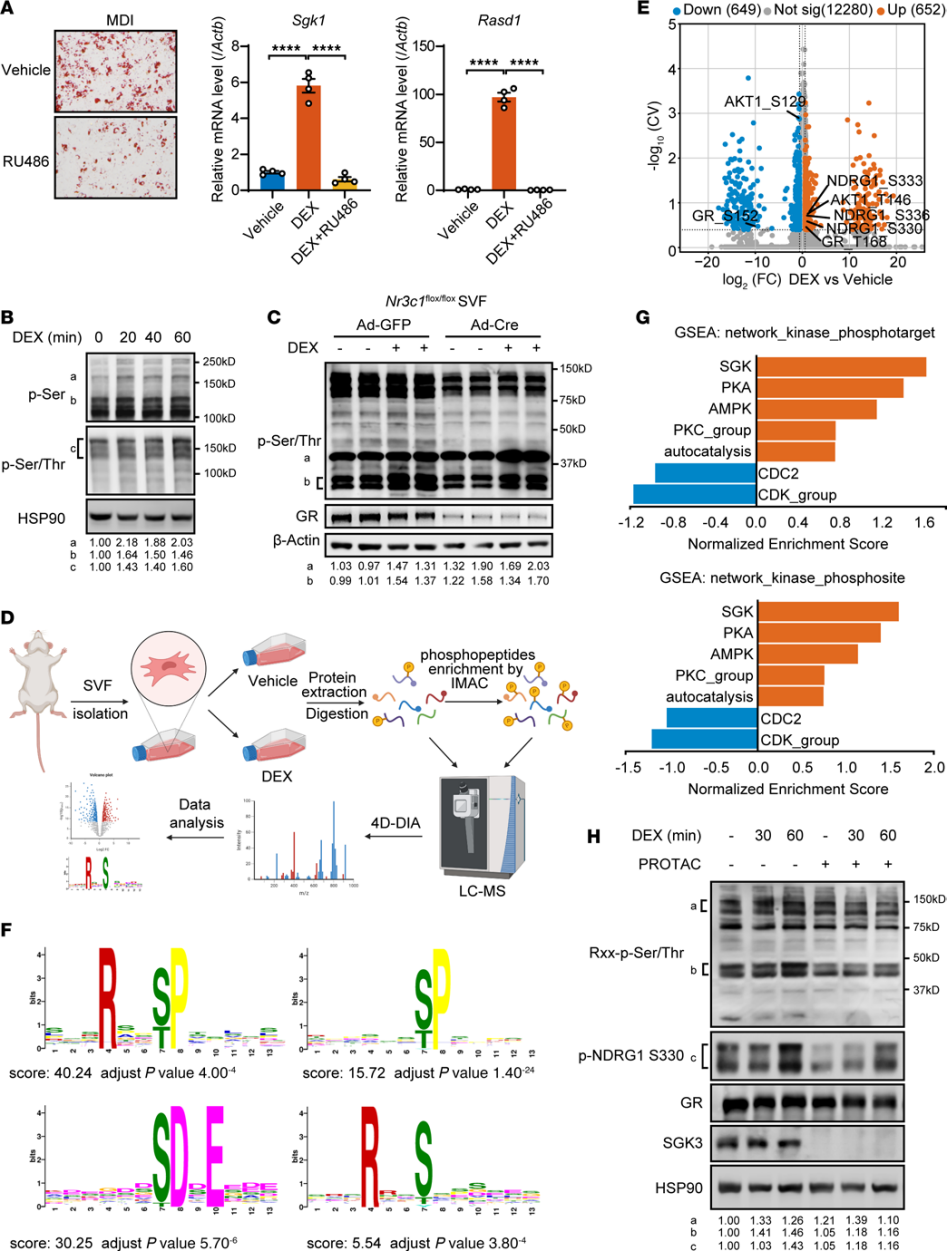
SGK3 is essential for protein phosphorylation in response to glucocorticoid stimulation. (**A**) Representative images of Oil Red O staining of differentiated preadipocytes treated with vehicle or RU486 (left). Scale bar: 200 μm. mRNA level of GR targets *Sgk1* (middle) and *Rasd1* (right) in preadipocytes treated with DEX or DEX and RU486 for 24 hours. *n* = 4 from different culture wells. (**B**) Protein phosphorylation in preadipocytes induced by DEX treatment at various time points. Relative band intensities were quantified (normalized to HSP90) and are shown below the figure. (**C**) Protein phosphorylation in GFP- or Cre-expressed *Nr3c1^fl/fl^* primary SVF cells induced by DEX treatment. Relative band intensities were quantified (normalized to β-actin) and are shown below the figure. (**D**) Experimental workflow to identify glucocorticoid-induced protein phosphorylation. (**E**) Volcano plot illustrating DEX-induced phosphorylation alterations. (**F**) Motif analysis of DEX-induced phosphorylation sites. (**G**) GSEA analysis of DEX-induced phosphorylation sites with datasets of network_kinase_phosphotarget and network_kinase_phosphosite. (**H**) SGK3 deficiency attenuates DEX-induced phosphorylation of RxxS/T peptides and phosphorylation of NDRG1 at serine 330. Relative band intensities were quantified (normalized to HSP90) and are shown below the figure. Identical sample aliquots were loaded on separate gels for immunoblotting analysis in **B**, **C**, and **H**. Data in **A** are represented as mean ± SEM, and 1-way ANOVA was used for statistical analysis. ****P < 0.0001.

Numerous studies have investigated the mechanisms of glucocorticoids’ effects through GR, and recent research has suggested GR-independent roles of glucocorticoids in regulating biological processes including adipogenesis. GR loss delays rather than prevents preadipocyte differentiation in vitro (8) and does not impair the development of adipose tissue in E18 mice embryos (9). Similar to GR loss, when we induced differentiation of cells treated with GR antagonist RU486 with the differentiation induction cocktail MDI, Oil Red O staining showed that inhibition of GR’s activity did not completely prevent preadipocyte differentiation (Figure 1A). The inhibitory effect by RU486 treatment was demonstrated by the total inhibition of the upregulation of GR’s downstream genes *Sgk1* and *Rasd1* by glucocorticoid under both normal culture medium and steroid-free charcoal-stripped medium (Figure 1A and Supplemental Figure 1A; supplemental material available online with this article; https://doi.org/10.1172/JCI186534DS1). These results suggest other mechanisms beyond GR’s function that promote adipogenesis by glucocorticoids.

To further investigate the mechanisms of glucocorticoid’s function beyond GR’s activity, we treated primary SVF cells isolated from murine inguinal WAT (iWAT) with DEX for a short time and found a set of proteins were phosphorylated on their serine and threonine sites after DEX treatment in an hour (Figure 1B). KO of GR (*Nr3c1*) reduced but did not eliminate DEX-induced phosphorylation at serine and threonine sites, suggesting that certain protein kinases are responsive to DEX-induced protein phosphorylation independent of GR (Figure 1C). To uncover fast responses from cells when exposed to glucocorticoid, we employed 4D-DIA proteomics and phosphoproteomics to measure total protein and phosphorylated peptides in primary SVF cells with or without DEX treatment (Figure 1D). A total of 5,747 proteins were identified, with only 92 proteins showing changes in abundance (Supplemental Figure 1B), suggesting that short-term treatment with glucocorticoid does not alter global protein levels. However, among approximately 15,000 quantified phospho-sites, we observed 652 upregulated phospho-sites and 649 downregulated phospho-sites (Figure 1E), indicating that glucocorticoid induces rapid and widespread changes in protein phosphorylation status. Gene Ontology (GO) analysis revealed a strong correlation with protein phosphorylation–associated terms and translation-associated terms, such as chromatin organization, regulation of RNA pol II, and mRNA transport (Supplemental Figure 1C). Furthermore, Kyoto Encyclopedia of Genes and Genomes (KEGG) pathway analysis indicated enrichment of glucocorticoid-induced phospho-proteins in autophagy, cell junctions, AMPK, and insulin signaling pathways, suggesting an interaction with energy metabolism in preadipocytes (Supplemental Figure 1D). Motif analysis of enhanced phospho-sites revealed enrichment in RxxS/T, S/TP, and SDxE classes (Figure 1F), implicating activation of various protein kinase families, including SGKs, AKTs, AMPK, MAPKs, and CDKs. To further validate DEX-mediated phosphorylation of RxxS/T motif–containing proteins, we conducted dose-response and time-course experiments in SVF cells. Our data revealed a clear dose- and time-dependent phosphorylation response, with RxxS/T motif phosphorylation intensity showing progressive enhancement under both elevated DEX concentrations and extended exposure durations during the first hour of treatment (Supplemental Figure 2, A and B). Moreover, other glucocorticoids, including prednisolone, corticosterone, and hydrocortisone, similarly promoted RxxS/T motif phosphorylation, mirroring the effects observed with DEX (Supplemental Figure 2C). This consistent response across multiple glucocorticoids suggests a class-wide capacity to induce rapid protein phosphorylation events.

Next, to determine whether glucocorticoid-induced protein phosphorylation requires GR signaling, we examined tamoxifen-inducible GR-KO cells after DEX treatment. Strikingly, we detected sustained phosphorylation of RxxS/T motifs in specific target proteins despite complete GR ablation (Supplemental Figure 2D). These findings demonstrate the existence of functional GR-independent mechanisms mediating glucocorticoid signaling. Through Gene Set Enrichment Analysis (GSEA), we identified SGK as the top enriched kinase family (Figure 1G). Given the shared substrates among SGK family members, this finding implies the potential involvement of the 3 SGK family kinases in orchestrating glucocorticoid-induced phosphoproteomic alterations. Glucocorticoids stimulate *Sgk1* mRNA transcription through GR, and SGK3, unlike SGK1 and SGK2, has been observed to display immediate increased kinase activity upon glucocorticoid treatment (27). This prompts the hypothesis that SGK3 mediates a response to glucocorticoids independent of GR to facilitate the rapid phosphorylation of RxxS/T motifs. Indeed, the absence of SGK3 by treating the cells with the specific SGK3 protein degrader SGK3 PROTAC suppressed DEX-induced RxxS/T phosphorylation and the phosphorylation of NDRG1 on serine 330 (Figure 1H) without affecting GR protein level, suggesting SGK3 rapidly phosphorylates its substrates under glucocorticoid treatment. Under classical conditions, SGK3 is synergistically activated by mTORC2 and the PI3K pathway in response to insulin; here, our data demonstrated that DEX also activated SGK3. To determine whether distinct upstream mechanisms mediate this activation, we compared SGK3 activity in cells treated with either DEX or insulin in the presence or absence of an SGK3 PROTAC degrader. We observed differential phosphorylation patterns of RxxS/T motif–containing proteins in response to DEX versus insulin. Notably, SGK3 PROTAC selectively abolished phosphorylation of specific bands (indicated by the letter c) in DEX-treated cells but not in insulin-treated cells (Supplemental Figure 2E). These findings suggest that glucocorticoids and insulin engage divergent pathways to activate SGK3. Further supporting this model, treatment with the PI3K inhibitor LY294002 only partially suppressed DEX-induced phosphorylation of RxxS/T motif–containing proteins (Supplemental Figure 2F), indicating that glucocorticoids activate SGK3 through both PI3K-dependent and PI3K-independent mechanisms.

### SGK3 deficiency protected mice from DEX- and HFD-induced obesity.

**Figure 2:**
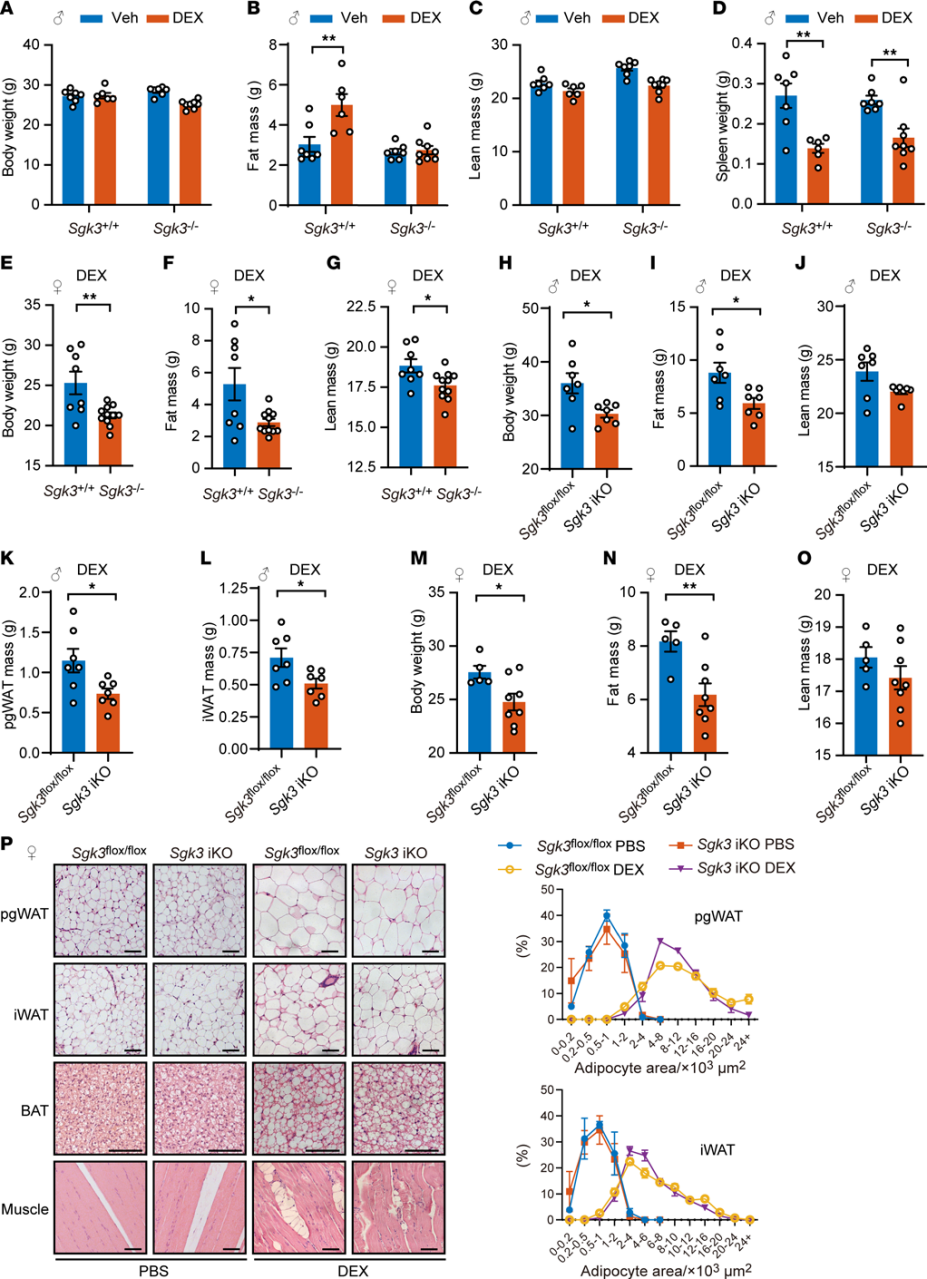
*Sgk3*-deficient mice are protected from glucocorticoid-induced obesity. (**A**–**D**) Body weight (**A**), fat mass (**B**), lean mass (**C**), and spleen weight (**D**) of vehicle or DEX-treated *Sgk3*^+/+^ and *Sgk3*^–/–^ male mice over 28 days. *n* = 7 for *Sgk3*^+/+^ (Veh), *n* = 6 for *Sgk3*^+/+^ (DEX), *n* = 7 for *Sgk3*^–/–^ (Veh), *n* = 8 for *Sgk3*^–/–^ (DEX). (**E**–**G**) Body weight (**E**), fat mass (**F**), and lean mass (**G**) of DEX-treated *Sgk3*^+/+^ and *Sgk3*^–/–^ female mice over 28 days. *n* = 8 for *Sgk3*^+/+^, *n* = 11 for *Sgk3*^–/–^. (**H–L**) Body weight (**H**), fat mass (**I**), lean mass (**J**), pgWAT weight (**K**), and iWAT weight (**L**) of DEX-treated *Sgk3^fl/fl^* and *Sgk3*-iKO (inducible KO by tamoxifen) male mice over 32 days. *n* = 7 for each group. (**M**–**O**) Body weight (**M**), fat mass (**N**), and lean mass (**O**) of DEX-treated *Sgk3^fl/fl^* and *Sgk3*-iKO female mice over 28 days. *n* = 5 for *Sgk3^fl/fl^* mice, *n* = 8 for *Sgk3*-iKO mice. (**P**) Representative images of H&E staining of pgWAT, iWAT, brown adipose tissue, and muscle of PBS- or DEX-treated *Sgk3^fl/fl^* and *Sgk3*-iKO female mice over 28 days. Scale bar: 100 μm. Related quantifications of adipocyte area of pgWAT and iWAT. *n* = 5 mice per group (>200 adipocytes are quantified for each mouse). Data in **A**–**D** are represented as mean ± SEM, and 2-way ANOVA was used for statistical analysis. Data in **E**–**O** are represented as mean ± SEM, and an unpaired 2-tailed *t* test was used for statistical analysis. Data in **P** are represented as mean ± SEM. *P < 0.05, **P < 0. 01.

Because one of the metabolic side effects of glucocorticoids is to promote obesity, we then evaluate the function of SGK3 in regulating adipose tissue under glucocorticoid treatment in vivo. We intraperitoneally injected 8-week-old *Sgk3*^–/–^ and control *Sgk3^+/+^* male mice with DEX daily, and found *Sgk3*^–/–^ mice were protected from DEX-induced fat mass gain compared with control *Sgk3^+/+^* mice (Figure 2, A–C), and there was no change in splenic weight loss (Figure 2D). Similarly, female *Sgk3*^–/–^ mice were also protected from DEX-induced fat mass increase (Figure 2, E–G). Thus, *Sgk3*-KO mice exhibited protection from glucocorticoid-induced obesity while remaining sensitive to its immunosuppressive effects, as shown by decreased spleen weight, suggesting SGK3 as a potential important target for mitigating glucocorticoid-induced metabolic side effects without affecting glucocorticoid’s immune suppression effects.

We and others found *Sgk3*-KO mice exhibit mild developmental defects, including developmental delays during infancy and adolescence (28). To mitigate the impact of developmental deficiencies observed in *Sgk3*^–/–^ mice, we next employed tamoxifen-induced KO mice by crossing *CAG*-Cre^esr1^ mice with *Sgk3^fl/fl^* mice, termed *Sgk3-*iKO mice. In accordance with the *Sgk3*^–/–^ mice, we also observed lower body weight, fat mass, perigonadal white adipose tissue (pgWAT) weight, and iWAT weight in both male and female *Sgk3*-iKO mice compared with *Sgk3^fl/fl^* mice, with no significant changes in lean mass and spleen weight (Figure 2, H–O, and Supplemental Figure 3, A–G). Moreover, while DEX treatment significantly induced brown fat “whitening” and increased lipid accumulation in the muscle of control animals, these adverse effects were greatly attenuated in both male and female *Sgk3*-iKO mice, further indicating *Sgk3*-iKO mice were protected from DEX-induced metabolic side effects (Figure 2P and Supplemental Figure 3H). However, no significant differences in adipocyte size were observed in iWAT and pgWAT tissues between *Sgk3*-iKO mice and *Sgk3^fl/fl^* mice (Figure 2P and Supplemental Figure 3H). These observations suggest that the distinctions in WAT tissues between *Sgk3*-iKO and *Sgk3^fl/fl^* mice are not attributed to reduced adipocyte size but rather to a decreased number of adipocytes, implying that SGK3 regulates glucocorticoid-induced adipogenesis in preadipocytes rather than mature adipocytes in vivo.

A more widespread cause of obesity is overnutrition, which induces adipogenesis in adipose tissue. A recent study also showed that mice fed an HFD exhibit higher plasma corticosterone (29). Given the role of SGK3 in adipogenesis and DEX-induced obesity, we questioned whether SGK3 deficiency could improve overnutrition-induced adipogenesis and obesity. To this end, *Sgk3^fl/fl^* and *Sgk3*-iKO mice were fed an HFD. As expected, SGK3 deficiency protected mice from HFD-induced body weight gain (Supplemental Figure 3I), and the mice displayed lower fat mass, pgWAT weight, and iWAT weight (Supplemental Figure 3, J, L, and M) as well as no change in lean mass (Supplemental Figure 3K). Thus, SGK3 inactivation protected mice from DEX- and HFD-induced obesity.

### SGK3 deficiency does not compromise normal hepatic or renal function.

Given SGK3’s established roles in regulating ion channels and its critical functions in cell survival and proliferation, we investigated whether *Sgk3* KO affects normal hepatic and renal function, particularly during chronic DEX administration. For liver histology analysis, we measured lipid content (via Oil Red O staining), fibrosis (via α-SMA staining), and overall macroscopic changes. For kidney histopathology, we examined kidney sections for signs of fibrosis and structural abnormalities. We also measured serum liver damage markers (ALT, AST, albumin, bilirubin) and serum kidney function markers (uric acid and urea). Our results showed that DEX treatment induced hepatic lipid accumulation and a slight increase in albumin levels without altering serum markers of liver or kidney function (Supplemental Figure 4, A–F). Additionally, SGK3 deletion did not significantly affect baseline liver or kidney function, suggesting that SGK3 is dispensable for maintaining homeostasis in these organs (Supplemental Figure 4, G–L). Notably, Oil Red O staining showed DEX-induced hepatic lipid accumulation was attenuated in *Sgk3*-iKO mice, with no differences in fibrosis or serum markers of liver and kidney function between WT and *Sgk3*-iKO mice (Supplemental Figure 4, M–S). These results confirmed that SGK3 deletion did not impair liver or kidney function, even after prolonged DEX treatment.

### The absence of SGK3 prevents preadipocyte differentiation.

**Figure 3:**
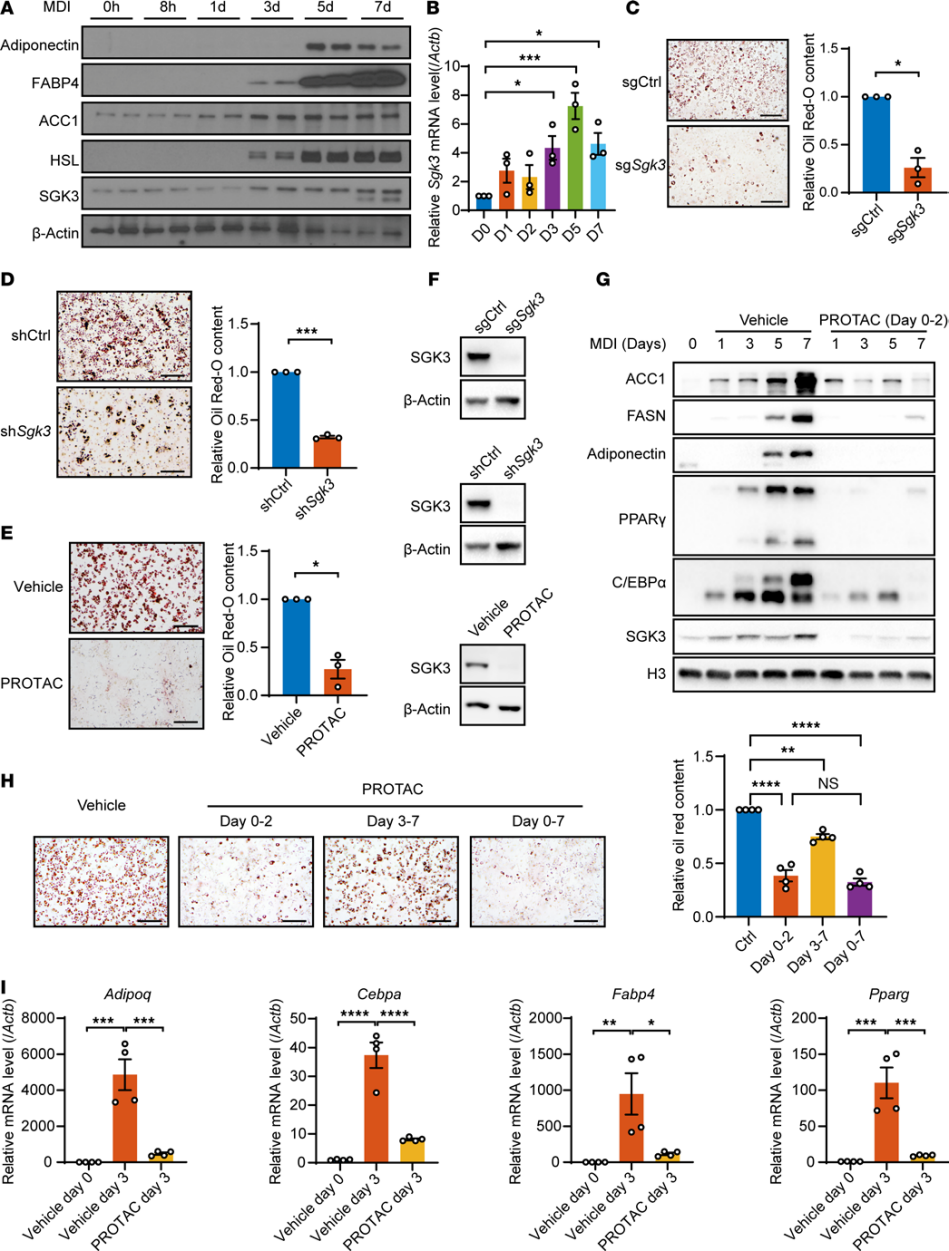
SGK3 regulates adipogenesis. (**A**) Protein level of SGK3, adiponectin, FABP4, ACC1, and HSL in 3T3-L1 preadipocytes during adipogenic differentiation. (**B**) mRNA level of *Sgk3* in 3T3-L1 preadipocytes during adipogenic differentiation. *n* = 3 independent assays. (**C**–**E**) Representative images of Oil Red O staining and related quantifications of differentiated *Sgk3* KO (**C**), *Sgk3* KD (**D**), and vehicle or SGK3 PROTAC-treated (**E**) preadipocytes on day 7. Scale bar: 200 μm. *n* = 3 independent assays. (**F**) Protein level of SGK3 in *Sgk3* KO, KD, and SGK3 PROTAC-treated preadipocytes. (**G**) Protein level of ACC1, FASN, adiponectin, PPARγ, C/EBPα, and SGK3 during preadipocyte differentiation with or without SGK3 PROTAC treatment during the first 2 days. (**H**) Representative images of Oil Red O staining (left) and related quantifications (right) of differentiated preadipocytes with or without SGK3 PROTAC treatment during indicated times on day 7. Scale bar: 200 μm. *n* = 4 independent assays. (**I**) mRNA level of *Adipoq*, *Cebpa*, *Fabp4*, and *Pparg* during preadipocyte differentiation with or without SGK3 PROTAC treatment during the first 2 days. *n* = 4 samples from different culture wells. Data in **B** and **H**–**I** are represented as mean ± SEM, and 1-way ANOVA was used for statistical analysis. Data in **C**–**E** are represented as mean ± SEM, and a paired 2-tailed *t* test was used for statistical analysis. ns, no significance; *P < 0.05, **P < 0.01, ***P < 0.001, ****P < 0.0001.

Next, we investigated whether SGK3 regulates adipogenesis in vitro. The upregulation of SGK3 expression during 3T3-L1 preadipocyte differentiation hints at its potential role in this process (Figure 3, A and B). To test this hypothesis, we generated immortalized SVF cell lines with both *Sgk3* KO and KD, and Oil Red O staining showed that a lack of SGK3 impaired adipogenesis (Figure 3, C, D, and F). Utilizing SGK3 protein degraders, we observed impaired adipogenesis upon SGK3 depletion in preadipocytes, corroborated by decreased lipid accumulation and reduced induction of adipogenesis markers (Figure 3, E and F). Consistent with these findings, the SGK3 kinase inhibitor GSK650394 (5 μM) potently suppressed adipogenesis, exhibiting comparable efficacy to SGK3 PROTAC at 0.3 μM, demonstrating that SGK3 kinase activity is required for its regulation on adipogenesis (Supplemental Figure 5A). This effect was further validated in primary SVF cells from *Sgk3*-KO mice, suggesting a cell-autonomous role for SGK3 in adipogenesis (Supplemental Figure 5B). This cell-autonomous effect of SGK3 on preadipocyte differentiation was further validated in FACS-sorted Lin^–^CD29^+^CD34^+^Sca1^+^CD24^+^ cells isolated from WT and *Sgk3*^–/–^ mice, with reduced adipogenic ability observed in *Sgk3*^–/–^ primary cells (Supplemental Figure 5C). Additionally, taking advantage of SGK3 PROTAC, we treated SVF cells with PROTAC at different stages during induced adipogenesis. Early-stage SGK3 PROTAC treatment significantly impaired adipogenesis, as evidenced by Oil Red O staining and the downregulation of adipogenic marker genes including *Adipoq*, *Cebpa*, *Fabp4*, and *Pparg* (Figure 3, G–I). In contrast, late-stage treatment had only a modest effect on adipogenesis, suggesting that SGK3 regulates adipogenesis primarily during the early induction stage. Similarly, SGK3 clearance in mature adipocytes showed no differences in Oil Red O staining (Supplemental Figure 5D). Together, these results demonstrate that SGK3 is crucial for early-stage adipogenesis.

### SGK3 regulates preadipocyte differentiation independent of GR.

**Figure 4:**
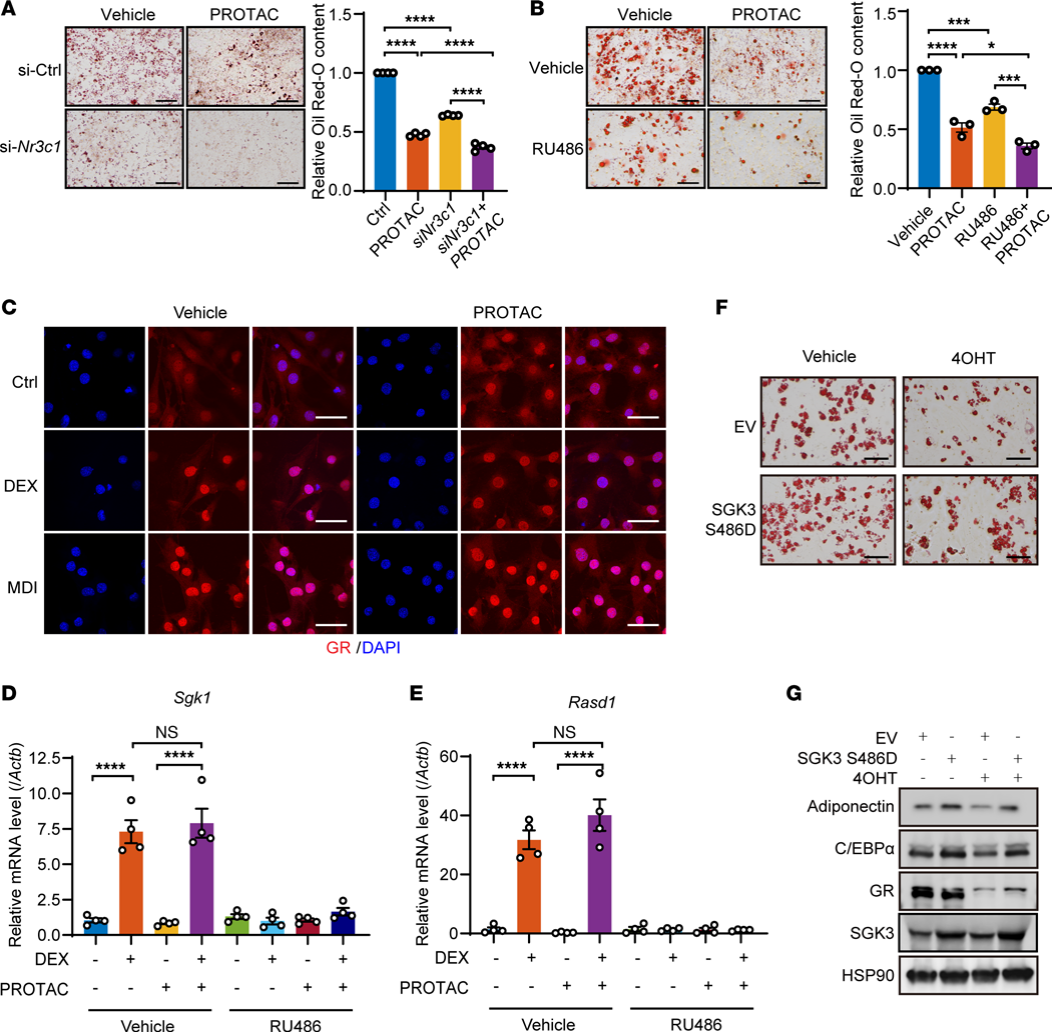
SGK3 regulates adipogenesis independently of GR signaling. (**A**) Representative images of Oil Red O staining and related quantifications of differentiated preadipocytes transfected with Ctrl or *Nr3c1* siRNA and/or treated with SGK3 PROTAC on day 7. Scale bar: 200 μm. *n* = 4 independent assays. (**B**) Representative images of Oil Red O staining and related quantifications of differentiated preadipocytes treated with RU486 and/or SGK3 PROTAC on day 21. Scale bar: 200 μm. *n* = 3 independent assays. (**C**) Representative immunofluorescence images of GR in SVF cells treated as indicated. Scale bar: 50 μm. (**D** and **E**) mRNA level of *Sgk1* (**D**) and *Rasd1* (**E**) in preadipocytes treated with DEX, SGK3 PROTAC, and RU486 as indicated. *n* = 4 samples from different culture wells. (**F**) Representative images of Oil Red O staining of differentiated preadipocytes with or without SGK3 S486D overexpression in control and GR-deficient cells on day 7. Scale bar: 200 μm. (**G**) SGK3 S486D overexpression effects on protein levels of adiponectin and C/EBPα on day 3 of differentiation in control and GR-deficient cells. Identical sample aliquots were loaded on separate gels for immunoblotting analysis in Figure **G**. Data in **A**, **B**, **D**, and **E** are represented as mean ± SEM, and 1-way ANOVA was used for statistical analysis. ns, no significance; *P < 0.05, ***P < 0.001, ****P < 0.0001.

Given GR’s known involvement in adipogenesis and SGK3’s rapid activation by glucocorticoids, we explored the interplay between SGK3 and the GR signaling pathway (8). To delve into the relationship between SGK3 and the GR signaling pathway, we treated control (si-Ctrl) and GR RNAi (si-*Nr3c1*) cells with or without SGK3 PROTAC. Oil Red O staining showed decreased differentiation efficiency with GR RNAi, further reduced by SGK3 PROTAC (Figure 4A). Correspondingly, SGK3 PROTAC and RU486, a GR antagonist, had an additive effect on suppressing preadipocyte differentiation, as evidenced by Oil Red O staining (Figure 4B). We then investigated the molecular changes in the GR signaling pathway in SGK3-deficient cells. The protein level of GR remained unchanged in the absence of SGK3 (Figure 1H), as did the nuclear translocation of GR in DEX or MDI treatment (Figure 4C); *Sgk1* and *Rasd1*, known GR downstream targets, were induced to comparable levels by DEX treatment in control and SGK3-deficient cells (Figure 4, D and E), suggesting SGK3 does not affect GR’s transcriptional regulation activity. To investigate potential additive effects of SGK3 and GR in adipogenesis regulation, we established stable cell lines constitutively expressing either empty vector or active SGK3 (S486D), combined with tamoxifen-inducible GR (*Nr3c1*) KO. We found that active SGK3 significantly enhanced adipogenesis in control cells, confirming its regulatory role in this process. While GR deficiency delayed adipogenesis in both control and SGK3-expressing cells, the SGK3-expressing lines maintained significantly higher differentiation rates than their respective controls in both WT and GR-deficient cells, as revealed by increased lipid droplet formation and an elevated protein level of adipogenic markers adiponectin and C/EBPα (Figure 4, F and G). The persistence of enhanced differentiation in SGK3-expressing GR-KO cells demonstrated that these regulators act through distinct mechanisms, with their combined effects being additive rather than epistatic. Taken together, these findings underscore the necessity of SGK3 in adipocyte differentiation and suggest SGK3 promotes adipocyte differentiation alongside the GR signaling pathway.

### SGK3 regulates adipocyte differentiation independently of cell cycle–associated functions.

To investigate how SGK3 regulates adipogenesis, we first examined its relationship with cell cycle regulation, a critical factor in adipocyte differentiation. Proliferation of adipocyte progenitors and subsequent differentiation sustain the pool of adipocytes in vivo. Cells undergo a specific cell cycle reentry during adipocyte differentiation in vivo when an HFD is administered, as well as induced adipocyte differentiation in cell lines in vitro (30). Since SGK3 regulates cell cycle in cancers (31), we questioned the impact of SGK3 absence on preadipocyte cell cycle–associated functions and performed EdU labeling assays in vitro. EdU labeling and staining revealed no changes in *Sgk3*-KO cells in proliferation-associated (Supplemental Figure 5E) or differentiation-associated (Supplemental Figure 5F) cell cycle reentry, as well as no changes in primary SVF cells isolated from WT and *Sgk3*-iKO mice (Supplemental Figure 5, G and H). An MTT assay also showed similar results in SVF cell proliferation (Supplemental Figure 5I). Thus, we concluded SGK3 regulates adipocyte differentiation independently of cell cycle–associated functions. Emerging evidence highlights SGK3’s crucial role in modulating arachidonic acid (AA) metabolism and catalase activity, which are key regulators of chromatin remodeling and oxidative stress that influence adipogenesis (32, 33). To investigate whether SGK3 regulates adipogenesis through these pathways, we quantified AA levels across multiple model systems. Interestingly, although we observed no significant changes in AA content in pgWAT of *Sgk3*-iKO mice, we detected elevated AA levels in both iWAT of *Sgk3*-iKO mice and SGK3 PROTAC-treated SVF cells (Supplemental Figure 6, A–C). This tissue-specific pattern contrasts with a previous report that SGK3 promotes AA synthesis in colorectal cancer cells (32). We also measured catalase mRNA and protein levels in our model systems and found no significant differences between control and SGK3 PROTAC-treated cells, or in the pgWAT tissue of WT and *Sgk3*-iKO mice (Supplemental Figure 6, D–H). Together, these data suggest that SGK3 does not regulate adipogenesis through regulating AA metabolism or catalase activity.

### SGK3 translocates to the nucleus during early adipocyte differentiation.

**Figure 5:**
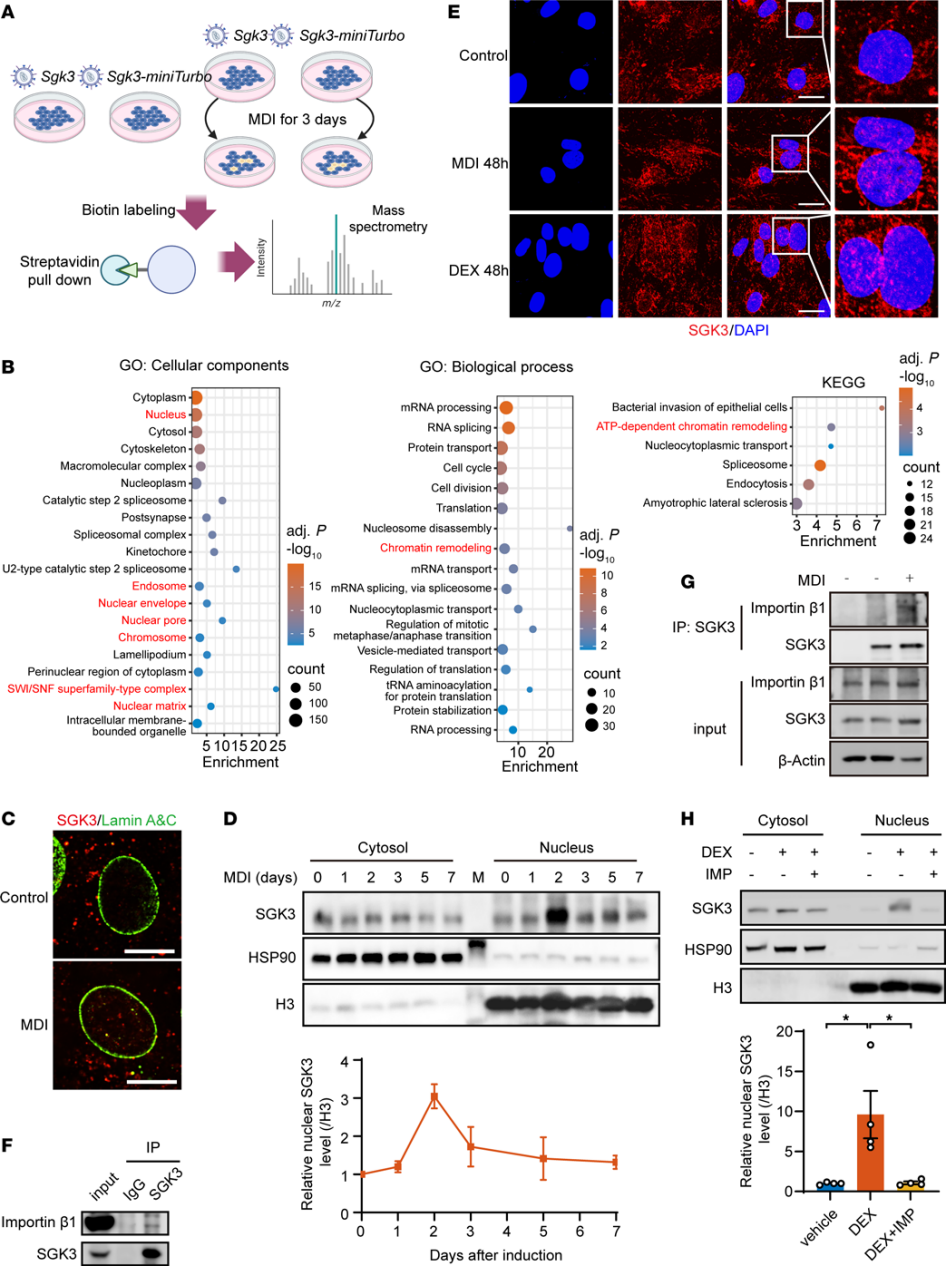
Glucocorticoid-induced nuclear SGK3 translocation during early stage of preadipocyte differentiation. (**A**) Strategy for identifying SGK3-interacting proteins. (**B**) GO enrichment analysis of SGK3-interacting proteins. (**C**) Representative immunofluorescence images of SGK3 and lamin A&C in differentiating primary SVF cells on day 2. Scale bar: 20 μm. (**D**) Localization of SGK3 in the cytosol fraction and nucleus at indicated time points during preadipocyte differentiation and related quantifications (normalized to H3). *n* = 3 independent assays. (**E**) Representative immunofluorescence images of SGK3 in preadipocytes treated with MDI or DEX for 2 days. Scale bar: 20 μm. (**F**) Co-IP shows SGK3 and importin β1 interaction in cells treated with MDI for 3 days. (**G**) Co-IP shows SGK3 and importin β1 interaction in cells treated with or without MDI for 2 days. (**H**) DEX-induced nuclear translocation of SGK3 with or without importazole treatment and related quantifications (normalized to H3) over 2 days. *n* = 4 samples from different culture wells. Data in **D** are represented as mean ± SEM. Identical sample aliquots were loaded on separate gels for immunoblotting analysis in Figure **D**. Data in **H** are represented as mean ± SEM, and 1-way ANOVA was used for statistical analysis. *P < 0.05.

To further understand the molecular mechanism of SGK3’s function in regulating adipocyte differentiation, we employed TurboID-based proximity labeling combined with mass spectrometry (MS) to identify potential SGK3 interaction proteins during adipogenesis (Figure 5A). We compared biotin-labeled proteins between cells treated with MDI for 0 and 3 days. SGK3 has been previously shown to localize in the cytoplasm and early endosome; when activated, SGK3 is recruited to the outer membrane of early endosomes by PI(3)P via its N-terminal phox domain, phosphorylated by PDK1 and mTORC2, and fully activated (34). Consistent with its known functions, GO cellular component analysis revealed significant enrichment of proteins localized to the cytoplasm and endosomes (Figure 5B). Unexpectedly, we also observed robust enrichment of nuclear-associated components, including the nucleus, nucleoplasm, and nuclear pore complex proteins (Figure 5B). These findings suggest potential nuclear localization of SGK3, a feature that has not been functionally characterized.

To confirm SGK3’s nuclear localization, we separated nuclear and cytosolic fractions of differentiating SVF cells. The nuclear localization of SGK3 was confirmed via immunofluorescence assays on day 2 after adipocyte differentiation induction (Figure 5C) and further validated through Western blotting (Figure 5D). We postulated that specific components within the MDI might trigger SGK3 translocation. Consequently, we treated SVF cells with different components of the MDI for 2 days and found DEX alone sufficiently induced SGK3 nuclear translocation (Figure 5E). Although synthetic glucocorticoids like DEX primarily signal through GR (35), endogenous glucocorticoids activate both GR and mineralocorticoid receptor (MR) nuclear translocation. We therefore examined whether these receptors facilitate SGK3 nuclear import. First, in a tamoxifen-induced GR-KO SVF cell line that retained MR expression, we observed that MDI-induced SGK3 translocation was independent of GR (Supplemental Figure 7A). On the other hand, treatment with the MR inhibitor finerenone demonstrated that SGK3 nuclear translocation was also MR independent (Supplemental Figure 7B). These results are consistent with our hypothesis that other undefined mechanisms mediate the activation of SGK3 by glucocorticoids in a receptor-independent pathway.

In pursuit of understanding the mechanism driving SGK3’s nuclear translocation, we examined nuclear-cytoplasmic transporter proteins among SGK3 interacting proteins (Supplemental Figure 7C). Importin β1 and exportin-1 emerged as the most enriched transporters, facilitating cargo movement from cytosol to nucleus and vice versa, respectively. Thus, we speculated that SGK3 translocation might rely on the importin β complex. Co-IP assays revealed a physical interaction between SGK3 and importin β1 (Figure 5F). Notably, MDI-induced adipogenic differentiation markedly enhanced this SGK3–importin β1 interaction (Figure 5G). Moreover, treatment with importazole, an inhibitor of the importin β complex, effectively blocked DEX-induced SGK3 translocation (Figure 5H). Hence, glucocorticoids induce SGK3’s nuclear translocation in an importin β–dependent manner.

### SGK3 is crucial for facilitating chromatin remodeling throughout the differentiation of adipocytes.

**Figure 6:**
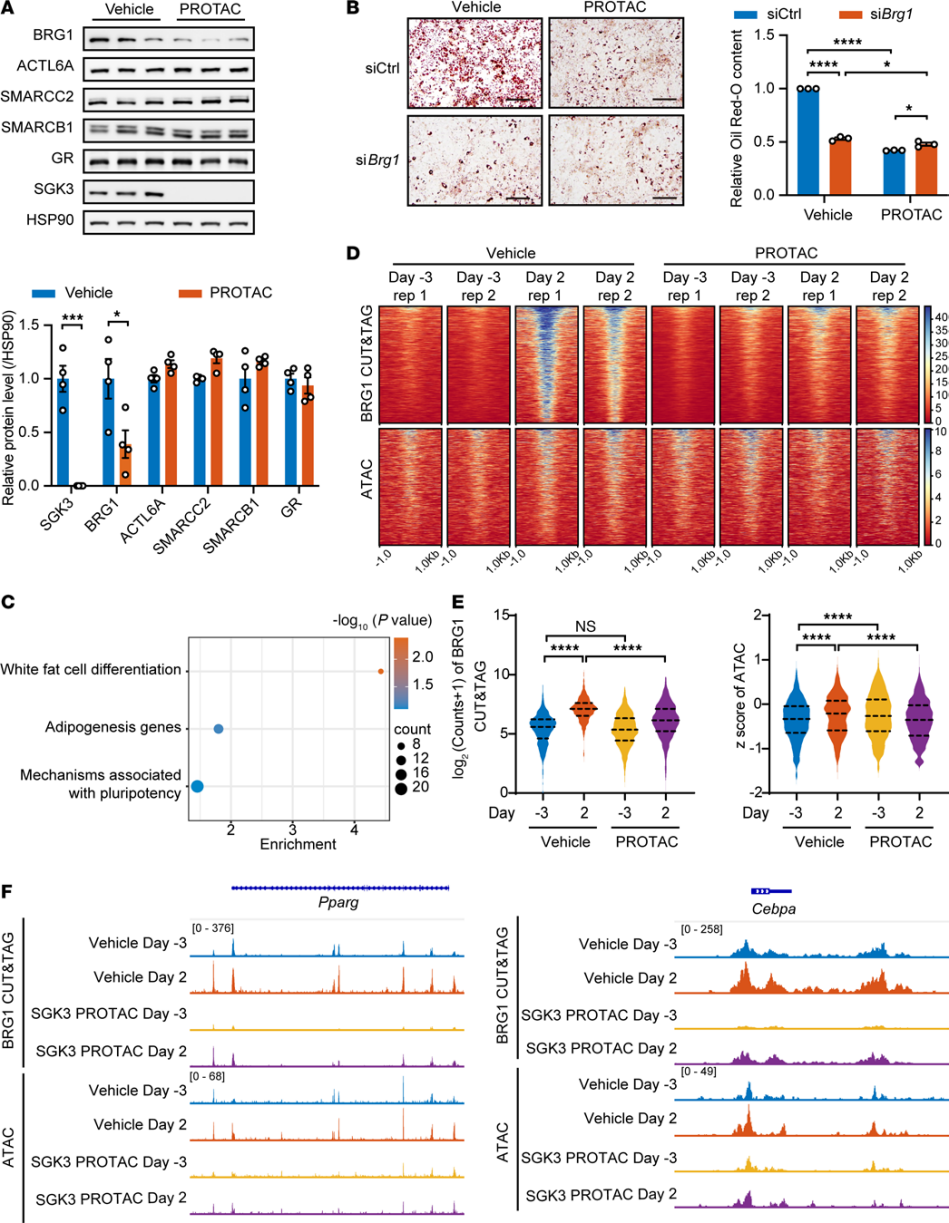
Deficiency of SGK3 impairs chromatin remodeling during preadipocyte differentiation. (**A**) Representative Western blot (upper) and related quantifications (lower) of BRG1, ACTL6A, SMARCC2, SMARCB1, GR, and SGK3 in SGK3 PROTAC-treated preadipocytes. *n* = 4 samples from different culture wells. (**B**) Representative images of Oil Red O staining (left) and related quantifications (right) of differentiated preadipocytes transfected with Ctrl or *Brg1* siRNA and/or treated with SGK3 PROTAC on day 7. Scale bar: 200 μm. *n* =3 independent assays. (**C**) Enrichment analysis of genes near the regions of induced BRG1 occupation during preadipocyte differentiation. (**D**) Heatmap of BRG1 CUT&TAG (upper) and ATAC-Seq (lower) signals with or without SGK3 PROTAC treatment during preadipocyte differentiation. (**E**) Quantification of BRG1 CUT&TAG signals and ATAC-Seq signals in regions of induced BRG1 occupation during preadipocyte differentiation. *n* = 1,158 regions. (**F**) BRG1 CUT&TAG and ATAC-Seq signals in the *Pparg* and *Cebpa* gene locus. Data in **A** are represented as mean ± SEM, and unpaired 2-tailed *t* test was used for statistical analysis. Data in **B** are represented as mean ± SEM, and 2-way ANOVA was used for statistical analysis. Identical sample aliquots were loaded on separate gels for immunoblotting analysis in **A**. Data in **E** are represented as mean and 25th to 75th percentile, and 1-way ANOVA was used for statistical analysis. ns, no significance; *P < 0.05, ***P < 0.001, ****P < 0.0001.

Adipocyte differentiation is intricately regulated by a network of transcription factors operating within a delicately balanced chromatin landscape (13). GO biological process analysis of the SGK3 interaction proteins showed that the proteins that mediate ATP-dependent chromatin remodeling were enriched among SGK3 interaction proteins (Figure 5B). The SGK3 interaction proteins were notably enriched with components of the SWI/SNF and BAF complexes, critical for chromatin remodeling (Figure 5B). Most components of the BAF complex were identified via SGK3 TurboID proximity labeling (Supplemental Figure 7D), suggesting SGK3’s involvement in differentiation-associated chromatin remodeling through the BAF complex. Moreover, the protein level of the key BAF complex component SMARCA4 (BRG1) was downregulated in PROTAC-treated SVF cells (Figure 6A). Notably, DEX-treated *Sgk3*-iKO mice showed reduced expression of BAF complex components (BRG1, ACTL6A) and adipogenic markers (PPARγ, C/EBPα) in pgWAT compared with controls, without changes in GR levels (Supplemental Figure 8, A and B). While ACTL6A remained unchanged, the BRG1 protein level was also decreased in pgWAT of untreated *Sgk3*-iKO mice (Supplemental Figure 8C). Tissue analysis revealed this BRG1 reduction was specific to pgWAT, not occurring in the liver or muscle in DEX-treated *Sgk3*-iKO mice (Supplemental Figure 8, D and E), demonstrating SGK3’s tissue-specific control of BRG1. These results further indicate that SGK3 regulates the function of the BAF complex independently of GR protein levels.

To establish the relationship between SGK3 and the BAF complex, we used RNAi to knockdown *Brg1* in SVF cells and treated cells with or without SGK3 PROTAC. Expectedly, BRG1-deficient cells exhibited reduced differentiation capability, which remained unaffected by SGK3 PROTAC treatment. In contrast, WT cells showed decreased differentiation capability upon SGK3 PROTAC treatment (Figure 6B). These findings suggest the reliance of SGK3 function on the BAF complex.

Next, to unravels the role of SGK3 on BRG1 chromatin occupation and subsequently the chromatin remodeling landscape during the early stage of preadipocyte differentiation, we performed BRG1 CUT&Tag and ATAC-Seq in primary SVF cells under vehicle or SGK3 PROTAC treatment, both before differentiation induction (day –3) and on day 2 post-induction. BRG1 CUT&TAG analysis identified 4,109 differential BRG1 occupied regions, of which 1,158 regions were upregulated, and 2,951 regions were downregulated in vehicle-treated cells (Supplemental Figure 8F), as well as a smaller number in SGK3 PROTAC-treated cells (1,550 differential occupied regions), and only 117 differential occupied regions overlapped between the 2 conditions (Supplemental Figure 8G). These results indicate that loss of SGK3 disrupts BRG1 chromatin localization during preadipocyte differentiation.

To obtain insights into the role of SGK3 on chromatin remodeling through BRG1, we further analyzed the chromatin accessibility in regions with differential BRG1 occupancy during preadipocyte differentiation. Adipogenesis is known to involve the activation of numerous genes that facilitate preadipocyte differentiation and metabolic remodeling. The binding of the BAF complex to regulatory elements of these genes aids in their activation. Accordingly, pathway analysis of genes near regions with enhanced BRG1 occupancy revealed enrichment in terms related to white fat cell differentiation and adipogenesis (Figure 6C). We focused on these loci with enhanced BRG1 binding and observed that the loss of SGK3 diminished BRG1 occupancy at these sites, indicating that SGK3 is necessary for BRG1 chromatin localization during adipogenesis (Figure 6, D and E). Consequently, the chromatin accessibility of these loci was also reduced by loss of BRG1 binding caused by the absence of SGK3 (Figure 6, D and E). These results suggest that the transcriptional program of preadipocyte differentiation is disrupted by the loss of SGK3 through the disruption of BRG1 chromatin localization on adipogenesis-associated genes. Consistent with these observations, similar changes in BRG1 occupation and chromatin accessibility were observed at *Cebpa* and *Pparg* gene loci, which are master transcription factors of adipogenesis (Figure 6F). Together, these results underscore the dependency of SGK3 function on chromatin remodeling during preadipocyte differentiation via BRG1.

### SGK3 interacts with BRG1 and regulates its protein level via the Ub-proteasome system.

**Figure 7:**
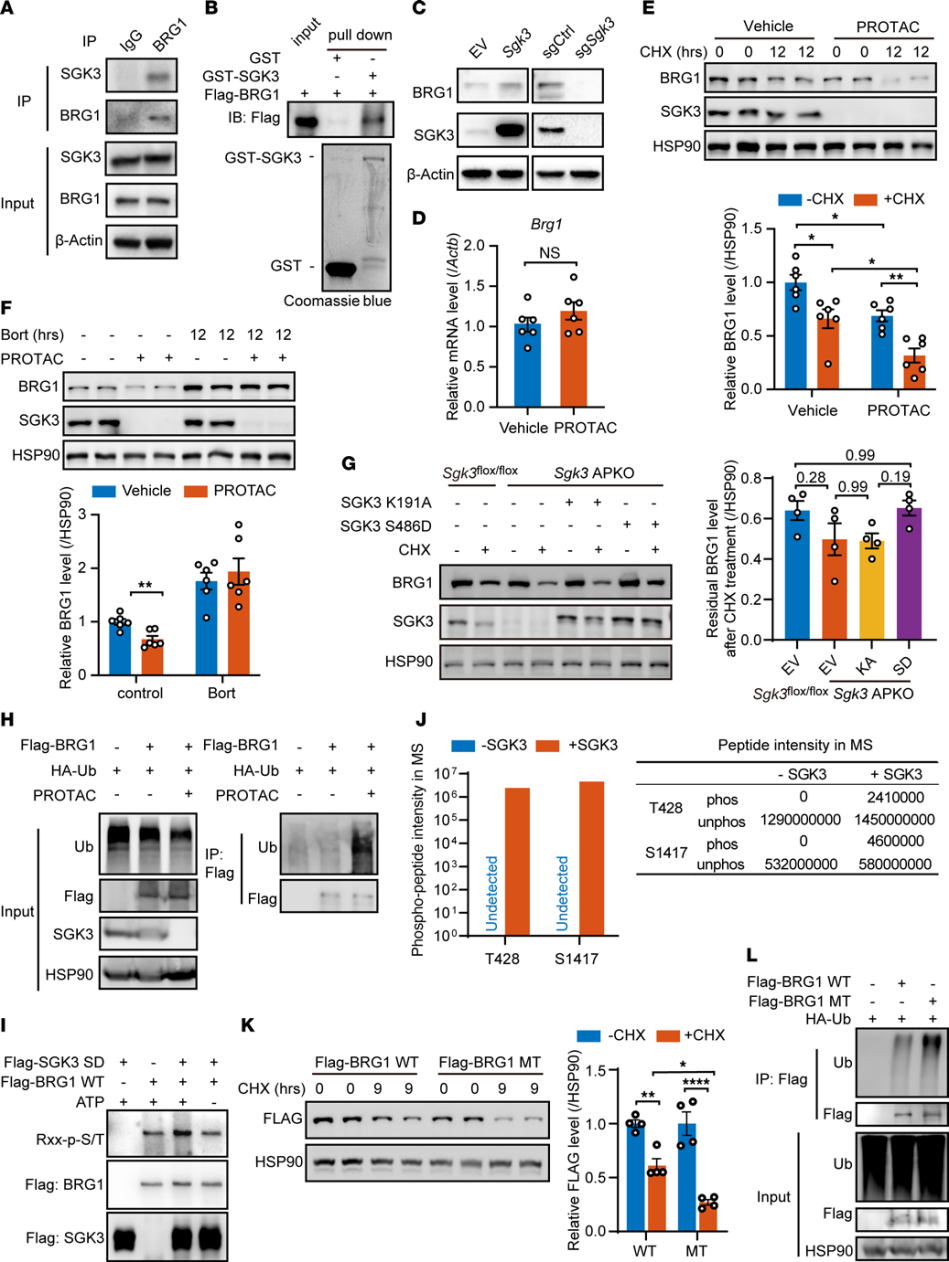
SGK3 regulates differentiation-associated chromatin remodeling via BRG1. (**A**) Co-IP assay indicates BRG1 interaction with SGK3. (**B**) Direct interaction between SGK3 and BRG1 detected by GST pulldown assay. (**C**) Protein level of BRG1 in *Sgk3* overexpression (left) or KO (right) cells. (**D**) mRNA level of *Brg1* in SGK3 PROTAC-treated cells. *n* = 6 samples from different culture wells. (**E** and **F**) Representative Western blot of BRG1 protein levels (upper) and related quantifications (lower) in vehicle or SGK3 PROTAC-treated preadipocytes measured under cycloheximide (**E**) or bortezomib (**F**) treatment, *n* = 6 samples from different culture wells. (**G**) Representative Western blot of BRG1 protein levels (left) and related quantifications (right) in *Sgk3^fl/fl^* and *Sgk3* APKO SVF cells expressing either kinase-dead (K191A) or constitutively active (S486D) SGK3 mutants treated with cycloheximide for 8 hours. *n* = 4 samples from different culture wells. (**H**) Ubiquitination level of Flag-BRG1 protein in HEK293T cells with or without SGK3 PROTAC treatment. (**I**) In vitro kinase assay shows SGK3 directly phosphorylates BRG1. (**J**) Intensity of 428-threonine and 1417-serine phosphorylated peptides of human BRG1 protein quantified by mass spectrometry (MS). (**K**) Representative Western blot of WT and T428A/S1417A mutant human BRG1 protein levels (left) and related quantifications (right) measured under cycloheximide treatment in HEK293T cells. *n* = 4 samples from different culture wells. (**L**) Ubiquitination level of WT and T428A/S1417A mutant human BRG1 proteins in HEK293T cells treated with or without SGK3 PROTAC. Data in **D**, **E**, **F**, **G,** and **K** represented as mean ± SEM. Unpaired 2-tailed *t* test used for statistical analysis in **D**. Two-way ANOVA used for statistical analysis in **E** and **K**. Multiple 2-tailed *t* tests used for statistical analysis in **F**. One-way ANOVA used for statistical analysis in **G**. Identical sample aliquots were loaded on separate gels for immunoblotting analysis in **H** and **L**. Data in **J** represented as value. *P < 0.05, **P < 0.01, ***P < 0.001, ****P < 0.0001.

Our subsequent objective was to uncover SGK3’s influence on chromatin remodeling through the BAF complex. We conjectured that the regulation of the BAF complex by SGK3 might depend on particular components within the complex. Given BRG1’s prominence in SGK3 interactions with the BAF complex, we speculated that SGK3 governs the BAF complex via BRG1 (Supplemental Figure 7D). To verify this, we conducted co-IP and GST pulldown assays. The co-IP assay confirmed the interaction between SGK3 and BRG1 in differentiating SVF cells (Figure 7A). Additionally, the GST pulldown assay utilizing purified GST or GST-SGK3 proteins as baits distinctly revealed the presence of the BRG1 protein band in the GST-SGK3 pulldown assay, affirming a direct interaction between SGK3 and BRG1 (Figure 7B).

We further confirmed that BRG1 protein levels were upregulated in SGK3-overexpressing cells while downregulated in SGK3-deficient cells (Figure 7C). *Brg1* mRNA levels remained unaffected in SGK3-deficient cells (Figure 7D). These results suggested that SGK3 regulates BRG1 expression in a posttranscriptional manner; thus, we examined BRG1 protein stability. Indeed, the stability of BRG1 protein was reduced in SGK3-deficient cells compared with vehicle-treated cells (Figure 7E) when translation was inhibited by cycloheximide treatment, and this change could be restored by bortezomib treatment, an inhibitor of proteosome (Figure 7F). To investigate whether SGK3 kinase activity regulates BRG1 stability, we reconstituted *Sgk3*-KO SVF cells with either constitutively active SGK3 (SGK3 S486D) or a kinase-dead mutant (SGK3 K191A) (28, 36). Under cycloheximide treatment, we observed that BRG1 degradation was significantly accelerated in *Sgk3*-KO cells compared with controls, and overexpression of active SGK3 rescued BRG1 stability while the kinase-dead mutant failed to prevent BRG1 degradation (Figure 7G). These results demonstrate that SGK3’s kinase activity is essential for maintaining BRG1 protein stability. Together with the observation of elevated polyubiquitination of BRG1 in SGK3-deficient cells (Figure 7H), we speculate that SGK3 modulates BRG1 stability via a ubiquitination-proteosome pathway.

### SGK3 directly phosphorylates BRG1.

Given the kinase nation of SGK3, we aimed to explore whether SGK3 regulates BRG1 stability through phosphorylation. With several RxxS/T substrate motifs present in the BRG1 protein sequence, we assessed BRG1 phosphorylation levels using an antibody against Rxx-p-S/T in an in vitro kinase assay. The result displayed an increased phosphorylation level of BRG1 protein upon incubation with SGK3 S486D (Figure 7I). Subsequent mass spectrometry experiments revealed that the threonine-428 site (matching the RxxS/T motif) and the serine-1417 site (matching the KxxS/T motif) of human BRG1 were phosphorylated by SGK3 in the in vitro assay (Figure 7J). These two sites were also conserved in mouse BRG1 protein as 428-threonine and 1384-serine. Next, we sought to determine whether the ubiquitination and protein stability of BRG1 are controlled by phosphorylation of T428/S1417. Indeed, the T428A/S1417A mutant of BRG1 displayed increased ubiquitination level and instability (Figure 7, K and L). Collectively, these findings provide evidence of a GR-independent glucocorticoid/SGK3/BRG1 axis governing chromatin remodeling crucial for adipocyte differentiation in vitro.

### SGK3 deficiency in adipocyte progenitors improves DEX-induced obesity.

To investigate the potential role of SGK3 in protecting against DEX-induced obesity within mature adipocytes, we created mature adipocyte–specific *Sgk3*-KO mice by breeding *Adipoq*-Cre mice with *Sgk3^fl/fl^* mice (Supplemental Figure 9A). These mice, termed *Sgk3-*AKO mice, were created to explore SGK3’s function beyond adipocyte differentiation. Analysis of body weight, fat mass, lean mass, iWAT weight, pgWAT weight, and liver weight revealed no differences between *Sgk3-*AKO mice and *Sgk3^fl/fl^* mice under both DEX-treated and HFD conditions (Supplemental Figure 9, B–O). These findings suggested that the beneficial effect of SGK3 deficiency in mitigating glucocorticoid-induced obesity is independent of mature adipocytes.

Having demonstrated impaired adipocyte differentiation in SGK3-deficient cells in vitro, we further investigated SGK3’s functional role in vivo by generating adipocyte progenitor–specific-KO (APKO) mice through the crossbreeding of *Pdgfrα*-Cre mice with *Sgk3^fl/fl^* mice, termed *Sgk3-*APKO mice. The KO efficiency was demonstrated by Western blotting of SGK3 in both pgWAT and iWAT (Supplemental Figure 10A). First, 8-week-old *Sgk3*-APKO mice and *Sgk3^fl/fl^* mice were intraperitoneally injected with DEX. After 35 days, *Sgk3*-APKO mice also displayed lower body weight, reduced fat mass, and decreased pgWAT weight compared with *Sgk3^fl/fl^* mice (Supplemental Figure 10, B–F). These observations supported the function of SGK3 in adipocyte progenitors in vivo. Moreover, we examined adipose tissue development in *Sgk3*-APKO mice. *Sgk3*-APKO neonates (postnatal day 5) exhibited reduced iWAT mass compared with controls (Supplemental Figure 10G), indicating that SGK3 plays a regulatory role in early iWAT adipogenesis in vivo. The formation of adipose tissue, albeit at reduced efficiency, demonstrates that SGK3 is not essential for adipogenesis. Together, these findings position SGK3 as a facilitator of adipogenesis.

Given the above, our study proved the requirement of SGK3 in glucocorticoid-induced obesity in mice. The protective function observed in *Sgk3*-KO mice was partially reliant on adipocyte progenitors, as previously mentioned, rather than on mature adipocytes.

### SGK3 deficiency inhibits adipogenesis in vivo.

**Figure 8:**
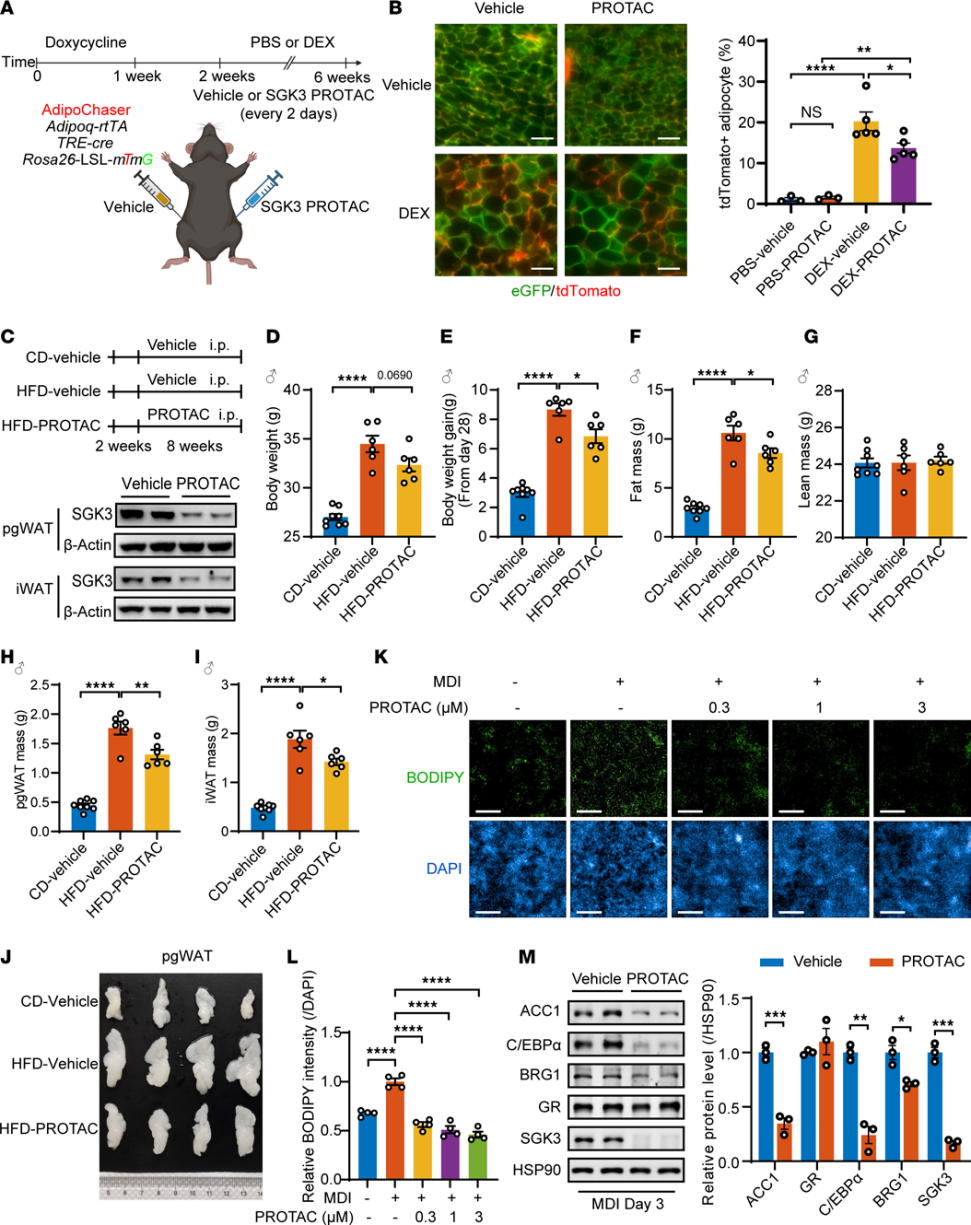
Pharmaceutical targeting of SGK3 attenuates HFD-induced obesity in mice and adipogenesis in vivo and in human cells. (**A**) Experimental workflow to examine the function of SGK3 in adipogenesis in vivo using AdipoChaser-*mT/mG* mice. (**B**) Effects of SGK3 PROTAC on adipogenesis in iWAT with or without DEX treatment. Left: representative images of iWAT sections showing preexisting (green) and newborn (red) adipocytes. Right: quantification of newborn adipocytes per field. Scale bar: 100 μm. *n* = 3 for PBS-vehicle and PBS-PROTAC groups, *n* = 5 for DEX-vehicle and DEX-PROTAC groups. (**C**) Experimental workflow (upper) to examine the function of SGK3 PROTAC in the treatment of HFD-induced obesity. Western blot (lower) was used for detecting SGK3 in pgWAT and iWAT of control and SGK3 PROTAC-treated mice. (**D**–**I**) Body weight (**D**), body weight change (**E**), fat mass (**F**), lean mass (**G**), pgWAT weight (**H**), and iWAT weight (**I**) of mice fed with chow diet and HFD fed with or without SGK3 PROTAC treatment. *n* = 8 for CD-vehicle, *n* = 6 for HFD-vehicle and HFD-PROTAC. (**J**) Representative images of pgWAT of mice fed with chow diet and HFD fed with or without SGK3 PROTAC treatment. (**K** and **L**) Representative images of BODIPY staining (**K**) and related quantifications (**L**) of differentiated human preadipocytes treated with SGK3 PROTAC at indicated concentrations. Scale bar: 500 μm. *n* = 4 samples from different culture wells. (**M**) Representative Western blot and related quantifications of ACC1, C/EBPα, BRG1, GR, and SGK3 in differentiating human preadipocytes with or without SGK3 PROTAC treatment on day 3. *n* = 3 samples from different culture wells. Identical sample aliquots were loaded on separate gels for Western blotting analysis in **M**. Data in **B**, **D**–**I**, **L**, and **M** are represented as mean ± SEM, and 1-way ANOVA was used for statistical analysis. *P < 0.05, **P < 0. 01, ***P < 0. 001, ****P < 0. 0001.

Finally, to further substantiate SGK3’s role in regulating adipogenesis in vivo, we employed AdipoChaser mice to investigate whether SGK3 deficiency indeed inhibits adipocyte differentiation in vivo. AdipoChaser mice were generated by crossing *Adipoq*-*rtTA*, *TRE*-*Cre*, *Rosa26*-STOP-*lacZ*, wherein mature adipocytes express LacZ upon doxycycline administration, but the newly developed adipocytes are negative for LacZ (37). This model allowed us to label mature adipocytes and to identify newly developed adipocytes after 1 month of DEX administration, with or without SGK3 PROTAC treatment. We induced Cre activity of WT AdipoChaser mice by 1 week doxycycline administration and started DEX administration a week later. Concurrently with the DEX regimen, we injected SGK3 PROTAC into the right part of iWAT to induce SGK3 degradation every 2 days, using vehicle as a control in the left part (Supplemental Figure 10, H and I). After 1 month of treatment, iWAT tissues were collected and stained. Sections revealed a greater number of blue-stained adipocytes in the right part compared with the left part of the iWAT tissues (Supplemental Figure 10J), indicating impaired adipogenesis in SGK3-deficient iWAT tissues. To further confirm this, we similarly performed experiments using the AdipoChaser*-mT/mG* mouse model, in which doxycycline induction labels preexisting adipocytes with GFP (green) and newly generated adipocytes with tdTomato (red), enabling clearer quantification (Figure 8A). Quantitative analysis showed comparable ratios of green (mature) to red (new) adipocytes in iWAT between control and SGK3 PROTAC-treated parts under basal conditions. However, DEX treatment induced a significant increase in adipogenesis in control iWAT, while this response was substantially blunted in SGK3 PROTAC-treated iWAT (Figure 8B). Furthermore, immunohistochemical analysis of cleaved caspase-3 in adipose tissue revealed no differences in apoptotic activity between DEX-treated and control groups, nor between vehicle and SGK3 PROTAC treatment (Supplemental Figure 10K), suggesting the change of adipocytes is not because of cell apoptosis. Together, these findings demonstrate that SGK3 regulates WAT adipogenesis in vivo.

### Pharmaceutical targeting of SGK3 attenuates HFD-induced obesity in mice.

Next, we studied whether pharmaceutical inhibition of SGK3 could improve obesity. To monitor the development of obesity, we fed mice an HFD for 2 weeks, followed by vehicle or SGK3 PROTAC injections every 2 days. A control group of mice was fed a chow diet and received vehicle injections (Figure 8C). After 8 weeks of treatment, the HFD-PROTAC group showed lower body weight, fat mass, pgWAT, and iWAT mass compared with the HFD-vehicle group, with no change in lean mass (Figure 8, D–J). We also performed glucose and insulin tolerance tests in HFD-fed mice treated with SGK3 PROTAC to assess their metabolic effects. Our results demonstrated that SGK3 PROTAC markedly improved both parameters compared with untreated controls (Supplemental Figure 11, A and B). Overall, these results suggest that pharmaceutical targeting of SGK3 can attenuate HFD-induced obesity.

To test the therapeutic potential for treating already obese animals, we treated *ob/ob* mice with SGK3 PROTAC for 6 weeks and checked the phenotypes. Although SGK3 PROTAC did not reduce fat mass in *ob/ob* mice, it significantly improved glucose and insulin tolerance (Supplemental Figure 11, C–J). This suggests that targeting SGK3 degradation may have therapeutic potential for metabolic dysfunction in obesity, independent of changes in adiposity. The absence of fat mass reduction implies that the metabolic benefits of SGK3 PROTAC are mediated through SGK3’s function in nonadipocyte cell types, which needs further exploration.

### SGK3 regulates human preadipocyte differentiation.

To further examine whether the function of SGK3 in preadipocyte differentiation is conserved between mice and humans, we utilized an immortalized human fibroblast cell line derived from the orbital adipose tissue of a patient with thyroid eye disease. We then induced differentiation of these cells with or without SGK3 PROTAC treatment. Consistently, SGK3 PROTAC treatment inhibited preadipocyte differentiation and lipid accumulation, as indicated by significantly reduced BODIPY intensities (Figure 8, K and L). Additionally, Western blot analysis showed that adipogenesis markers ACC1 and C/EBPα, as well as BRG1, were significantly downregulated in differentiating human preadipocytes (Figure 8M).

In summary, our study unveiled glucocorticoid-induced SGK3 activation in adipocyte progenitors. SGK3 deficiency impaired adipocyte differentiation both in vitro and in vivo, and consequently ameliorated glucocorticoid-induced obesity in a mouse model. More importantly, pharmaceutical targeting of SGK3 attenuated HFD-induced obesity in mice, and the role of SGK3 in regulating adipogenesis was conserved between humans and mice. Mechanistically, we uncovered the translocation of SGK3 to the nucleus upon glucocorticoid exposure, a process reliant on the importin β complex. Nuclear SGK3 phosphorylated BRG1, the core subunit of the BAF complex, and maintained BRG1 protein stability. In the absence of SGK3, BRG1 expression decreased, leading to compromised chromatin remodeling required for adipocyte differentiation (Supplemental Figure 11L).

## Discussion

Glucocorticoids primarily exert their function through the GR, but other pathways beyond the GR pathway can also be activated by glucocorticoids. For instance, glucocorticoids have been shown to directly bind and activate GPR97, although its downstream signaling remains elusive (11). Several studies have demonstrated that GR accelerates but is dispensable for glucocorticoid-induced adipogenesis (8–10), suggesting the involvement of other pathways. In this context, we proposed a noncanonical pathway through which glucocorticoids regulate adipogenesis in parallel to GR. Our findings reveal that glucocorticoids rapidly induce global changes in protein phosphorylation, partially mediated by the nutrient- and growth factor–responsive kinase SGK3. We demonstrated a mechanism by which SGK3 contributes to glucocorticoid-induced adipogenesis through the phosphorylation of the core subunit BRG1 within the chromatin remodeling SWI/SNF complex. Moreover, our results from *Sgk3*-KO mice showed protection against glucocorticoid-induced obesity while retaining sensitivity to glucocorticoids’ immune suppression effects, highlighting SGK3 as a potential therapeutic target for addressing the metabolic side effects of glucocorticoids.

Numerous studies have demonstrated that glucocorticoids induce epigenetic alterations by activating their receptor, GR. For instance, GR recruits CBP and HDAC1 to target promoter sites by interacting with the RelB/p52 transcription factor, leading to dynamic acetylation and deacetylation of H3K9 (38). Additionally, glucocorticoids can promote chromatin remodeling, altering the accessibility of GR-binding sites to the transcriptional machinery (39). Our findings here reveal a potentially novel mechanism by which glucocorticoids regulate chromatin accessibility through modulating the stability of the core SWI/SNF complex subunit, BRG1. Chronic glucocorticoid treatment not only activates SGK3 but also induces its nuclear translocation, where it binds to BRG1 and directly phosphorylates it. This phosphorylation of BRG1 by SGK3 during adipocyte differentiation confers resistance to ubiquitination-mediated protein degradation, thereby promoting chromatin remodeling for the expression of key adipogenesis genes. Further investigations are necessary to explore whether these phosphorylation sites also regulate other components of the SWI/SNF complex and their interactions with chromatin.

Both class 1 and class 3 PI3Ks act as positive regulators of adipogenesis (40, 41). SGK3 kinase activity is activated by both class 1 and class 3 PI3Ks (22) and is also induced by glucocorticoid treatment (27), highlighting SGK3’s pivotal role in adipogenesis regulation. Indeed, our findings demonstrated that KO of *Sgk3* or degradation of SGK3 using SGK3 PROTAC significantly impairs adipocyte differentiation. NDRG1, a substrate of SGK family kinases (including SGK1, SGK2, and SGK3), has been reported to activate adipogenesis by promoting the transcription of key adipogenic genes (42). Our results indicate that, in addition to NDRG1, SGK3 directly alters chromatin status by phosphorylating the core subunit BRG1 of the chromatin remodeling SWI/SNF complex. Therefore, under adipogenic stimuli, SGK3 likely coordinates multiple pathways, including class 1 and class 3 PI3Ks, mTORC2, and glucocorticoid-activated pathways, leading to global gene expression changes necessary for adipocyte differentiation. We observed SGK3’s nuclear localization after chronic glucocorticoid treatment, despite its typical endosomal localization. Although importin β1 is known to mediate SGK3 translocation into the nucleus, the detailed mechanism of SGK3 shuttling between the cytoplasm and nucleus requires further exploration.

Specifically knocking out *Sgk3* in mature adipocytes using *Adipoq*-Cre had no effect on DEX-induced fat accumulation. Our in vitro data also showed that downregulation of SGK3 by SGK3 PROTAC treatment did not inhibit adipogenesis after the induction stage under adipogenic MDI treatment, Together, these findings suggest that SGK3 primarily regulates the early phase of adipogenesis. However, adipocyte progenitors specific to *Sgk3*-KO mice using *Pdgfrα*-Cre do not fully phenocopy the global *Sgk3*-KO mice under chronic glucocorticoid treatment, implying the involvement of SGK3 in other cell types as well. Given SGK3’s role in regulating insulin secretion in islet beta-cells (43), further investigation into SGK3’s function in other tissues under glucocorticoid treatment would be valuable.

Our data reveal that *Sgk3*-KO mice exhibit substantial protection against chronic glucocorticoid-induced obesity while remaining sensitive to glucocorticoid’s immunosuppressive effects. Since glucocorticoids are commonly used to treat inflammatory diseases, addressing their metabolic side effects is critical for patient management. Our findings suggest that SGK3 could serve as a potential therapeutic target to mitigate these metabolic complications without compromising the antiinflammatory efficacy of glucocorticoids. Additionally, SGK3 PROTAC shows promise as a tool for targeting SGK3, with our experiments demonstrating improved outcomes in HFD-fed mice, including reduced body weight, fat mass, and decreased weight of pgWAT and iWAT. These results underscore SGK3 as a viable pharmacological target for managing glucocorticoid- or overnutrition-induced obesity. Further investigation is worth doing to clarify the treatment benefits in the context of chronic glucocorticoid application. Notably, SGK3 PROTAC treatment in *ob/ob* mice significantly improved glucose homeostasis and insulin sensitivity (Supplemental Figure 11, C–J), despite unchanged adiposity. These findings demonstrate that SGK3 inhibition can ameliorate obesity-associated metabolic dysfunction independent of fat mass reduction.

The marked metabolic benefits conferred by SGK3 inhibition under both DEX and HFD challenge establish it as a promising therapeutic target for glucocorticoid-induced and obesity-related metabolic disorders. Given its widespread expression and involvement in diverse physiological processes, the safety profile of SGK3 inhibition requires thorough evaluation. In metabolism, SGK3 contributes to glucose homeostasis, as *Sgk3*/*Akt2* double-null mice exhibit impaired glucose tolerance, reduced plasma insulin, and diminished β-cell proliferation, highlighting its role in pancreatic islet function (43). SGK3 also modulates intestinal glucose absorption via the SGLT1 transporter (44). However, *Sgk3*-KO mice retain normal glucose homeostasis under baseline conditions, although these mice display wavy fur and curly vibrissae due to keratinocyte defects (28, 43), suggesting functional redundancy in metabolic regulation. Beyond metabolism, SGK3 governs ion transport systems, including calcium-phosphorus metabolism through phosphorylation of TRPV5/6 channels (45, 46). Phenotypic studies of *Sgk3*-KO mice reveal mild skeletal effects (subtly reduced bone density and phosphaturia) without major renal or calcium excretion abnormalities. The therapeutic potential of SGK3 inhibition has been particularly promising in oncology, where it overcomes drug resistance in HER2+ breast cancer and counteracts rapamycin resistance via mTORC1 reactivation (26, 47). These beneficial effects, combined with the mild phenotypes observed in KO models, suggest that systemic SGK3 inhibition may be well tolerated. Our finding that SGK1 protein levels are elevated in the adipose tissue of *Sgk3*-iKO mice suggests functional compensation by other SGK family members, which may mitigate potential toxicity risks. Together with observations from our and other labs showing that *Sgk3*-KO mice maintain normal growth, development, and adult metabolic parameters, with only transient developmental delays (28), these findings suggest SGK3 as a promising therapeutic target with a favorable safety profile.

In summary, our results highlight the critical role of SGK3 in glucocorticoid-induced chromatin remodeling and adipogenesis. SGK3 appears to function as a central hub integrating signals from glucocorticoids and growth factors like insulin, thereby promoting adipocyte differentiation through the regulation of epigenetic alterations. The retained immune suppression function of glucocorticoids in *Sgk3*-KO mice suggests that modulating SGK3 expression could be a relevant target for mitigating the metabolic side effects of glucocorticoids.

## Methods

### Sex as a biological variable.

Our study examined male and female animals, and similar findings are reported for both sexes.

### Animals.

*Sgk3* global KO mice (*Sgk3*^–/–^) were obtained from C57BL/6 zygotic injection of small guide RNAs targeting *Sgk3* and S.p.Cas9 mRNA at the Genome Modification Facility, Harvard University (48). *Sgk3^fl/fl^* mice (stock S-CKO-03505) were obtained from Cyagen. *Nr3c1^fl/fl^* mice (stock NM-CKO-200201) were obtained from Shanghai Model Organisms. The *CAG*-Cre^esr1^ (stock 004682) and *Pdgfrα*-Cre (stock 013148) mice were obtained from The Jackson Laboratory. *Adipoq*-Cre mice were provided in-house. Inducible *Sgk3* global KO mice (*Sgk3*-iKO), adipocyte progenitor–specific *Sgk3*-KO mice (*Sgk3*-APKO), and mature adipocyte–specific *Sgk3*-KO mice (*Sgk3*-AKO) were obtained by breeding *Sgk3^fl/fl^* mice with *CAG*-Cre^esr1^, *Pdgfrα*-Cre, and *Adipoq*-Cre mice, respectively. To induce Cre activity of *Sgk3*-iKO mice, tamoxifen dissolved in corn oil was intraperitoneally injected at a dose of 75 mg/kg for 5 consecutive days. The *Adipoq*-*rtTA* *TRE*-*Cre* *Rosa26*-STOP-*lacZ* AdipoChaser mice were provided in-house. The *Adipoq-rtTA TRE-Cre ROSA26-mT/mG* AdipoChaser mice were provided in-house. To induce Cre activity of AdipoChaser mice, drinking water was supplemented with doxycycline hyclate at a dose of 1 mg/mL for 7 days. The SGK3 PROTAC was dissolved in 3.5% DMSO/2.1% Tween 80/PBS. For injection into the iWAT, 50 μL of vehicle or SGK3 PROTAC emulsion was injected into the left or right side every 2 days, starting 1 week after Cre induction. Water-soluble DEX at a concentration of 1 mg/mL in PBS was intraperitoneally injected at a dosage of 5 mg/kg/day for 8-week-old mice or as otherwise specified. An HFD was administered to 6-week-old mice or as otherwise indicated. For SGK3 PROTAC-treated HFD-fed mice, 6-week-old mice were first fed HFD for 2 weeks, followed by intraperitoneal injections of SGK3 PROTAC at a dose of 15 mg/kg every 2 days for 8 weeks unless otherwise specified.

All mouse strains were maintained on a C57BL/6J background. Sibling or age-matched mice were used for all the experiments. All mice experiments were approved by the IACUC of the Shanghai Institute for Nutrition and Health, Chinese Academy of Sciences. All mice were housed in a temperature-controlled, specific pathogen–free barrier facility under a 12-hour light/12-hour dark cycle.

### Cell culture and treatments.

The HEK293T cell line was obtained from the Cell Bank, Type Culture Collection Committee, Chinese Academy of Sciences and cultured in DMEM supplemented with 10% FBS and 1% penicillin/streptomycin in a humidified incubator with 5% CO_2_ at 37°C. Immortalized WAT SVF cells were provided in-house and cultured in DMEM supplemented with 20% FBS and 1% penicillin/streptomycin in a humidified incubator with 5% CO_2_ at 37°C. To induce differentiation of immortalized WAT SVF cells into adipocytes, cells were cultured in DMEM supplemented with 10% FBS until they reached confluence. They were then treated with the adipogenic cocktail MDI (0.5 mM IBMX, 1 μM DEX, 1 μM rosiglitazone, 5 μg/mL insulin) for 48 hours, followed by maintenance in 5 μg/mL insulin for 4–6 days. Primary SVF cells were isolated from iWAT of WT, *Nr3c1^fl/fl^*, or *CAG*-Cre^esr1^ *Nr3c1^fl/fl^* mice. Briefly, mice were euthanized, and iWAT tissues were collected, washed with PBS, minced into small pieces, and digested with 300 U/mL collagenase I at 37°C for 30 minutes. The resulting cell suspension was filtered through a 70 μm strainer, centrifuged, and the pellet was collected and seeded. The medium was changed after 4 hours, and cells were maintained in DMEM/F12 supplemented with 10% FBS. SVF cells derived from *CAG*-Cre^esr1^ *Nr3c1^fl/fl^* mice were immortalized through lentiviral transduction of the SV40 large T antigen. To induce differentiation into adipocytes, cells were cultured in DMEM/F12 supplemented with 10% FBS until they reached confluence. They were then treated with the adipogenic cocktail MDI (0.5 mM IBMX, 1 μM DEX, 5 μg/mL insulin, 2.5 μM rosiglitazone) for 72 hours, followed by maintenance in 5 μg/mL insulin for 4–6 days. The SGK3 PROTAC was dissolved in DMSO and added to the culture medium at a final concentration of 0.3 μM at the indicated time points. For glucocorticoid treatment, cells were cultured with 1% FBS overnight or cultured with 5% charcoal-stripped FBS (CS-FBS) as indicated in figure legends. CS-FBS was prepared by incubating FBS with dextran-coated charcoal overnight, followed by filtration through a 0.22 μm membrane. To inhibit GR activity, RU486 was added to the culture medium at a final concentration of 10 μM, either 30 minutes before DEX treatment or throughout preadipocyte differentiation. The human immortalized preadipocyte cell line was provided in-house and was cultured and induced to differentiate as previously reported (49). The cells were cultured in DMEM/F12 supplemented with 10% FBS and 1% penicillin/streptomycin. To induce differentiation into adipocytes, cells were cultured in DMEM/F12 supplemented with 10% FBS until they reached confluence. They were then treated with the adipogenic cocktail MDI (0.5 μM IBMX, 1 μM DEX, 5 μg/mL insulin, 2.5 μM rosiglitazone) for 72 hours, followed by maintenance in 5 mg/mL insulin and 2.5 mM rosiglitazone for another 10 days.

### Statistics.

All quantifications of adipocyte area were conducted using ImageJ (NIH). Statistical analyses were performed using GraphPad Prism. The statistical differences between control and experimental groups were determined by 2-tailed *t* test (2 groups), 1-way ANOVA (for more than 2 groups), or 2-way ANOVA (for 2 independent experimental variables), with corrected *P* values of less than 0.05 considered significant. The statistical tests performed and definition of *n* in this study are indicated in the figure legends. EdgeR was used for statistical differences of BRG1-occupied regions. For Figure 2, A–P; Figure 8, B–G; Supplemental Figure 3, A–E; Supplemental Figure 7, B–I; and Supplemental Figure 8, B–F, *n* represents the number of mice. For Figure 1A; Figure 3I; Figure 4, D and E; Figure 5G; Figure 6A; Figure 7, D–F, and J; Figure 8, I and J; and Supplemental Figure 3H, *n* represents the number of cells that were analyzed. For Figure 3, B–E, and H; Figure 4, A and B; Figure 5D; Figure 6B; and Supplemental Figure 4C, *n* represents biological replicates. For Supplemental Figure 4, D–G, *n* represents analyzed microscopic fields. For Figure 6E, n represents the number of analyzed genomic foci. For cell culture Western blotting, each sample within each biological replicate corresponds to 1 well from a tissue culture plate. For mouse adipose tissue Western blotting, each sample corresponds to protein extract from 1 mouse.

### Study approval.

All animal studies were approved by the IACUC of Shanghai Institute of Nutrition and Health (SINH-2022-ZB-2).

### Data availability.

Values for all data points in graphs are reported in the Supporting Data Values file. The BRG1 CUT&TAG and ATAC-Seq data generated in this study are available in NCBI’s Gene Expression Omnibus (GEO) database under accession number GSE268382.

## Author contributions

QC, FZ, AAS, and BZ conceived the project. QC and BZ wrote the manuscript. QC, JG, YL, TD, JL, YZ, YD, Q Zhang, ZZ, MZ, CW, and BZ performed experiments and analyzed results. QD, Q Zhai, J Qin, J Qiu, MS, FZ, and AAS contributed to the discussion and edited the manuscript.

## Supplementary Material

Supplemental data

Unedited blot and gel images

Supporting data values

## Version 1 08/06/2025

In-Press Preview

## Version 2 10/01/2025

Electronic publication
