# Supplementary material for: Serum- and glucocorticoid-induced kinase 3 orchestrates glucocorticoid signaling to facilitate chromatin remodeling during murine adipogenesis
Source: J Clin Invest. 2025 Jul 24;135(19):e186534. doi: 10.1172/JCI186534 (PMC12483569; doi:10.1172/JCI186534)
Supplement: Supplemental data [file jci-135-186534-s037.pdf]

Supplemental methods

Plasmids, sgRNA and shRNA

The full-length and truncated human *Brg1*, *Sgk3*, *Sgk3-miniTurbo* cDNA were cloned into the pCDH-CMV-MSC-EF1-puro vector. The full-length murine *Sgk3* cDNA was cloned into the pGEX-4T-1 vector. The sgRNAs were cloned into the lentiCRISPRv2 vector. The shRNAs were cloned into the pLKO.1-puro vector.

Lentiviral packaging

A total of 24μg of plasmids, comprising either lentiCRISPRv2 or pLKO.1, were co-transfected with VSVG and Δ8.9 into HEK293T cells at a ratio of 12:7.5:4.5. The culture medium was collected and filtered over the subsequent 72 hours.

RNA isolation and real-time PCR

Total RNA was extracted using Trizol (15596026, Thermo) following the manufacturer's instructions. Subsequently, cDNA was synthesized utilizing HiScript III RT SuperMix (R323, Vazyme), with 1 μg of total RNA employed in each cDNA synthesis reaction according to the manufacturer's protocol. For RT-qPCR, SYBR green master mix (Q711, Vazyme) was utilized.

Immunoblotting

Cells and tissues were lysed in RIPA buffer (50 mM Tris-HCl pH 7.4, 150 mM NaCl, 1% Triton X-100, 1% deoxycholate, 0.1% SDS) supplemented with

protease and phosphatase inhibitors (P1006, P1082, P1092, Beyotime). Lysates were centrifuged at 12,000g for 15 min, and the resulting supernatants were collected and subjected to protein concentration measurement using the BCA assay (P0012, Beyotime). Subsequently, the proteins were separated by SDS-PAGE and transferred onto nitrocellulose membranes (HATF00010, Millipore). The membranes were incubated with primary antibodies (SGK3, 8573, BRG1, 49360, RXX-p-S/T, 9614, p-NDRG1 S330, 11899, p-NDRG1 T346, 5482, NDRG1, 9485, SMARCC2, 12760, PPARγ, 2435, C/EBPα, 8178, Adiponectin, 2789, FABP4, 2120, ACC1, 3676, HSL, 4107, Ub, 3936, SGK1, 12103, p-AKT T308, 13038 and AKT, 4691 are obtained from Cell Signaling Technology; GR, 24050-1-AP, FLAG, 80010-1-RR, 66008-4-Ig, HSP90, 60318-1-Ig, β-Actin, 66009-1-Ig, H3, 17168-1-AP, GST, 66001-2-Ig, ACTL6A, 10341-1-AP and DPF1, 21769-1-AP are obtained from Proteintech; Pan Phospho-Serine/Threonine, AP0893, SMARCB1, A23466, ARID1A, A19570, Catalase, A11780 and MR, A3308 from abclonal, Pan Phospho-Serine, 612546 from BD, importin β1, AB2811 are obtained from Abcam) overnight at 4°C, followed by incubation with HRP-conjugated secondary antibodies for 1 hour at room temperature. Finally, protein bands were visualized using the ECL detection system (WBKLS0500, Millipore or SQ202, Epizyme).

Quantitative proteomics and phosphoproteomics

Cells were subjected to overnight serum starvation followed by treatment with vehicle or dexamethasone for 1 hour. Subsequently, cells were harvested and

frozen, and then provided to 4D-DIA MS by PTM Biolabs Inc. Hangzhou, Zhejiang. Briefly, samples were extracted using lysis buffer (8 M urea, 1% protease inhibitor cocktail, 1% phosphatase inhibitor) and sonicated. Cell debris were removed by centrifugation, and the supernatants were precipitated by 20% (m/v) TCA. The precipitated proteins were washed and redissolved in 200 mM TEAB and digested by trypsin at 1:50 trypsin-to-protein mass ratio. The resulting peptides were reduced with 5 mM dithiothreitol, alkylated with 11 mM iodoacetamide, and desalted by Strata X SPE column. For phosphorylated peptides enrichment, IMAC microspheres were used additionally. The tryptic peptides were separated via HPLC and subsequently analyzed using Orbitrap Exploris 480 with a nano-electrospray ion source.

To prevent N/A values, the intensities of each phosphor-site and proteins were adjusted by adding 1. The intensities of each phosphor-site were then corrected by corresponding protein intensity and used for furtherer analysis. Fold changes and coefficients of variation (CV) of fold changes were calculated, with phosphor-sites exhibiting fold change ≥1.5 or ≤0.67 with CV ≤ 0.4 were defined as differential site. Motif analysis was performed by MoMo Motif-x (https://meme-suite.org/meme/index.html). GSEA analysis was performed in webgestalt (https://www.webgestalt.org/).

Proximal labeling-MS

Immortalized iWAT SVF cells stably expressing either SGK3 or SGK3-

miniTurbo were used for proximal labeling in each experimental condition. Briefly, Cells were incubated with 0.5 mM biotin overnight and then harvested in lysis buffer (0.5% NP-40, 150 mM NaCl, 50 mM Tris-HCl pH 7.5, 0.5mM EDTA) supplemented with protease and phosphatase inhibitors (P1006, P1082, P1092, Beyotime). Cell debris were removed by centrifuging at 4 °C, 12000g for 15 min, and the lysates were incubated with streptavidin beads overnight at 4°C. Biotinylated proteins were eluted in boiling 1× SDS loading buffer and subjected to SDS-PAGE followed by Coomassie staining. Gels were precisely cut and submitted to MS system by NCPSS, shanghai, CAS.

Net unique peptide numbers were calculated as the unique peptide numbers of miniTurbo group minus those of the corresponding control group. The criteria for selecting the candidate proteins for further study are as follows: (1) net unique peptide number in Day 3 sample ≥4, (2) net unique peptide number should exhibit a ≥1.5-fold change when comparing Day 3 sample with Day 0 sample. Candidate proteins meeting these criteria were submitted to David (https://david.ncifcrf.gov/home.jsp) for enrichment analysis. Enriched terms were sorted and selected on their *P* value.

Co-Immunoprecipitation (Co-IP)

Cells were lysed in lysis buffer (0.5% NP-40, 150 mM NaCl, 50 mM Tris-HCl pH7.5, 0.5 mM EDTA) supplemented with protease and phosphatase inhibitors (P1006, P1082, P1092, Beyotime). Cell debris were removed by centrifuging at

4 °C, 12000g for 15 min, and lysates were incubated with antibodies at 4°C overnight followed by adding protein A/G magnetic beads (P2012, Beyotime). The immunoprecipitants were washed 5 times with lysis buffer before being boiled and analyzed by western blotting using standard methods.

Ubiquitination Assay

HEK293T cells expressing Flag tagged BRG1 were lysed in RIPA buffer (50 mM Tris-HCl pH 7.4, 150 mM NaCl, 1% Triton X-100, 1% deoxycholate, 0.1% SDS) supplemented with protease, phosphatase inhibitors (P1006, P1082, P1092, Beyotime) and 10 mM N-Ethylmaleimide (E1271, Sigma). Cell debris were removed by centrifuging at 4 °C, 12000g for 15 min, and lysates were incubated with M2-Flag magnetic beads (A36797, Thermo) at 4°C overnight. The immunoprecipitated complexes were washed 5 times with lysis buffer before being boiled and analyzed by western blotting using standard methods.

Protein purification, GST pull-down and *In vitro* Kinase Assay

HEK293T cells were transiently transfected with SGK3 or BRG1 expression plasmids, and harvested 48h later using lysis buffer (1% Triton X-100, 150 mM NaCl, 20 mM HEPES pH7.5, 0.5 mM EDTA) supplemented with protease inhibitors (P1006, Beyotime). Cell debris were removed by centrifuging at 4 °C, 12000g for 15 min, and lysates were incubated with M2-Flag magnetic beads (A36797, Thermo) at 4°C for 2 hours. The immunoprecipitants complexes were washed 3 times with lysis buffer and 2 times with lysis buffer containing an

additional 100 mM NaCl. The immunoprecipitated proteins were eluted by incubating 3×Flag peptides at 4°C for 30 min.

GST tagged SGK3 protein or GST protein were purified from *E. coli.* BL21 transformed with pGEX-4T-1-*Sgk3* or empty vector. Briefly, *E. coli.* were induced by 0.1 mM IPTG (ST098, Beyotime) at room temperature overnight with slow shacking and then were lysed in lysis buffer (1% Triton X-100, 150 mM NaCl, 20 mM HEPES pH7.5, 0.5mM EDTA) supplemented with protease and phosphatase inhibitors (P1005, P1082, P1092, Beyotime) and ultrasonicated. Cell debris were removed by centrifuging at 4 °C, 12000g for 15 min, and lysates were incubated with GST beads at 4°C overnight. The immunoprecipitated complexes were washed 5 times with lysis buffer.

For GST pull down assay, Flag tagged BRG1 was incubated with GST beads loaded with GST or GST tagged SGK3 in PBST (1× PBS with 0.5% Triton x-100) overnight.

For *in vitro* kinase assays, Flag tagged BRG1 and SGK3 proteins were incubated in kinase assay buffer (10 mM $MgCl_2$, 50 mM Tris-HCl, 0.1 mM EDTA, 1 mM DTT, 0.5 mM ATP) at 30°C for 1h. For phosphorylation sites identification, proteins were eluted by boiling them in 1× SDS loading buffer and applied to SDS-PAGE followed by Coomassie staining. Gels were precisely cut and submitted to MS system by NCPSS, shanghai, CAS.

Oil Red O staining

Differentiated adipocytes were fixed with 4% paraformaldehyde (PFA) in PBS for 10 min, followed by washing with PBS and 60% isopropanol. Subsequently, the cells were stained with Oil Red O dye for 30 minutes and then rinsed with 60% isopropanol for 1 minute, followed by a water rinse. Oil Red O-stained cells were directly imaged using stereo microscope. Quantification of Oil Red O content was performed after extraction with isopropanol by measuring light absorbance at 450 nm.

BODIPY staining

Differentiated adipocytes were fixed with 4% PFA and washed three times with PBS. Samples were then incubated with 5 μg/mL BODIPY and 10 μg/mL DAPI in PBS. After incubating in the dark for 30 minutes, the samples were washed 4 to 5 times with PBS, and images were captured using Operetta CLS (PerkinElmer, USA). ImageJ software was used to quantify the BODIPY signal in each image.

Nuclear and Cytoplasmic Protein Extraction

Cells were harvested and subjected to centrifugation to obtain whole cell lysates, which were subsequently collected. Debris were re-suspended using a hypotonic lysis buffer (10 mM HEPES pH 7.9, 1.5 mM $MgCl_2$, 10 mM KCl, 0.5% NP-40, 1 mM EDTA) supplemented with protease and phosphatase inhibitors (P1006, P1082, P1092, Beyotime) and then incubated on ice for 15 min with gentle pipetting. Lysate was then centrifuged at 3,000 × g for 5 min, and the

supernatants were collected as the cytosol fraction. The remaining debris were washed 3 times and collected as the nuclear fraction.

BRG1 CUT&TAG

100,000 SVF cells during early differentiation for each condition were used for BRG1 CUT&TAG assays. Libraries were constructed according to the user manual (TD903, Vazyme) with additional spike-in supplementary (NS101, Vazyme). NGS were performed by Genewiz Inc. Suzhou China. Data analysis was performed in the Galaxy platform. Briefly, adaptors were trimmed by Cutadapt (4.6 + galaxy 1), pair-end reads were aligned to mm10 using Bowtie2 (2.5.0 + galaxy 0), improperly paired reads and reads with mapQuality < 30 or mapping to the mitochondria were filtered by filter BAM (2.5.2 + galaxy 1). After filtering, reads were subjected to MACS2 (2.2.9.1+ galaxy 0) for peak calling with an FDR *q*-value ≤0.0000001. Peeks were combined by Bedtools (2.30.0) across samples, and corresponding counts were quantified and corrected by spike-in counts. Differentially BRG1 occupied regions were identified by edgeR (3.36.0 + galaxy 4) with P value ≤ 0.01 and log2FC ≤-1 or ≥1. Peek annotation was performed by ChIPseeker (1.28.3 + galaxy 0). Visualization was performed by bamCoverage (3.5.4 + galaxy 0) with correction by spike-in counts and IGV. Heatmap were generated by computeMatrix (3.5.4 + galaxy 0) and plotHeatmap (3.5.4 + galaxy 0).

ATAC-seq

50,000 SVF cells during early differentiation for each condition were used to analyze chromatin accessibility. Libraries were constructed according to the user manual (N248-01A, Novoprotein). NGS were performed by Genewiz Inc. Suzhou China. ATAC-seq data analysis was performed in the Galaxy platform. Briefly, adaptors were trimmed by Cutadapt (4.6 + galaxy 1), pair-end reads were aligned to mm10 using Bowtie2 (2.5.0 + galaxy 0), improperly paired reads and reads with mapQuality < 30 or mapping to the mitochondria were filtered by filter BAM (2.5.2 + galaxy 1), duplicate reads were removed by MarkDuplicates (3.1.1.0). After filtering, reads were subjected to MACS2 (2.2.9.1+ galaxy 0) for peak calling with an FDR *q*-value ≤0.0000001. Visualization was performed by bamCoverage (3.5.4 + galaxy 0) and IGV. Heatmap were generated by computeMatrix (3.5.4 + galaxy 0) and plotHeatmap (3.5.4 + galaxy 0).

Histological analysis.

Tissues were fixed, embedded in paraffin, and then sectioned at 7 μm and stained with hematoxylin and eosin (H&E) following the manufacturer's instructions (G1120, Solarbio). The histopathological images were captured using a microscope and adipocyte area were analyzed using Image J software.

Immunohistochemistry

Paraffin-embedded sections were deparaffinized and rehydrated using standard protocols, while cryosections were rehydrated directly. Endogenous

peroxidase activity was quenched with 3% H_2O_2 for 10 min. Antigen retrieval was performed using citrate buffer (Beyotime, P0081), followed by blocking with 5% goat serum for 1 h at room temperature. Sections were incubated overnight at 4°C with primary antibodies (α-SMA, 1:1000, 80008-1-RR, Proteintech; SGK3, 1:100, 12699-1-AP, Proteintech) followed by secondary antibodies (RGAR011, Proteintech) incubation for 1 hour at room temperature. Signal detection was performed using a DAB kit (Proteintech, PR30018), followed by counterstaining with hematoxylin.

Immunofluorescence

Cells grown on glass coverslips were fixed in 4% paraformaldehyde (PFA) for 10 min and blocked with 5% goat serum. Primary antibodies (SGK3, 1:50, 12699-1-AP, Proteintech, Lamin A/C, 1:50, 4777, Cell Signaling Technology) were applied overnight at 4°C followed by secondary antibodies (RGAR004, RGAM002, Proteintech) incubation for 1 hour at room temperature. Images were captured by confocal microscopy (FV1200 or SpinSR, Olympus).

New-born adipocyte analysis of AdipoChaser mice

For *AdipoChaser-mTmG* mice, iWAT tissues were fixed in 4% PFA, de-hydrated in 30% sucrose, and embedded in OCT. 20 μm cryosections were re-hydrated, and images were captured using a microscope. For *AdipoChaser-LacZ* mice, iWAT tissue samples were subjected to staining using the *in situ* β-galactosidase staining kit (RG0039, Beyotime) following the manufacturer's

instructions, and then fixed in 4% PFA, de-hydrated in 30% sucrose, embedded in OCT, and sectioned into 20 μm slices, and images were captured using a microscope.

ITT and GTT

Mice were fasted for 6 hours followed by i.p. injection of insulin (0.75U/kg for HFD fed mice, 1U/kg for *ob/ob* mice) or glucose (1g/kg for HFD fed mice and *ob/ob* mice). Blood glucose concentrations were measured at the indicated time points using a glucometer.

ALT, AST, Albumin, TBil, uric acid and urea assay

Mice blood was collected before mice sacrificing. Serum was collected by centrifuging at 3000 rpm for 10 min after coagulation, and stored at -80 °C. ALT (20152400366, SSUF), AST (20152400367, SSUF), albumin (E2062, applygen), TBil (BC5185, solarbio), uric acid (BC1365, solarbio) and urea (BC1535, solarbio) were detected according to manufacture.

Arachidonic acid detection

Cells or tissues were homogenized in 1 mL 15% MeOH, centrifuged at 2500 *rpm* for 10 min. Supernates were collected and mixed with 1M AA-NH_4Ac pH 3.0, 6 mL Hexane/MTBE, and centrifuged at 2500 *rpm* for 8 min. the upper layer was collected, and repeated the extraction step for 2 times. The mixture evaporated, and reconstituted in 50 μL ddH_2O/acetonitrile/formic acid, and detected using a LC-MS/MS system with an Agilent 1200 high performance LC

system (Agilent Technologies, USA), coupled to a 4000Q TRAP triple quadrupole (QQQ) mass spectrometer (AB Sciex, USA) equipped with a Turbo Ion Spray source was operated in the negative-ion multiple-reaction monitoring (MRM) mode.

MTT

1000 cells were seeded into 96-well plates and let them reach attachment for 12h. 10 μL MTS (G9683, Promega) were added into cells and incubated for 2h at day 0 and day 3. A490 was detected and calculated for cell viability.

EdU incorporation and detection

To determine cell proliferation, cells in 70% confluence were incubated with 50μM EdU (S1661, Selleck) for 2 hours. To determine differentiation-associated cell cycle re-entry, cells were induced with MDI for 24h and then incubated with 50μM EdU for 16 hours. For EdU detection, cells were washed with PBS and incubated with reaction buffer of EdU-488 staining kit (CX002, Epizyme) for 2 hours.

Supplemental Figures

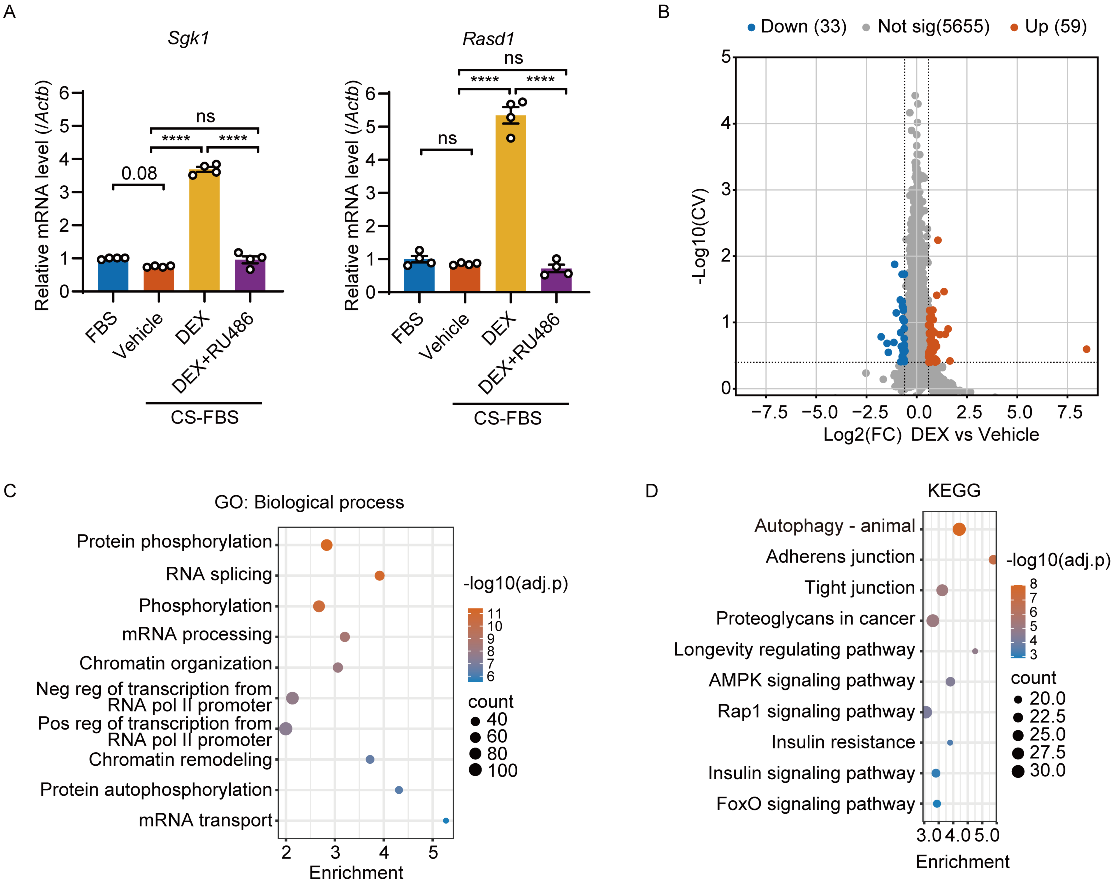

Supplemental Figure 1. Enrichment analysis of DEX-induced phosphorylated proteins.

(**A**) mRNA level of GR targets, *Sgk1* (left) and *Rasd1* (right), in preadipocytes cultured with FBS or charcoal-stripped FBS (CS-FBS) with DEX or DEX and RU486 treatment for 24h. *n* = 4 from different culture wells. (**B**) Volcano plot depicting DEX-induced protein changes. (**C**) Gene Ontology (GO) analysis of biological processes associated with phosphorylated peptides following DEX treatment. (**D**) KEGG pathway enrichment analysis of phosphorylated peptides following DEX treatment. Data in **A** were represented as mean ± SEM, and

One-way ANOVA was used for statistical analysis. "ns" indicates no significance, ****P < 0. 0001.

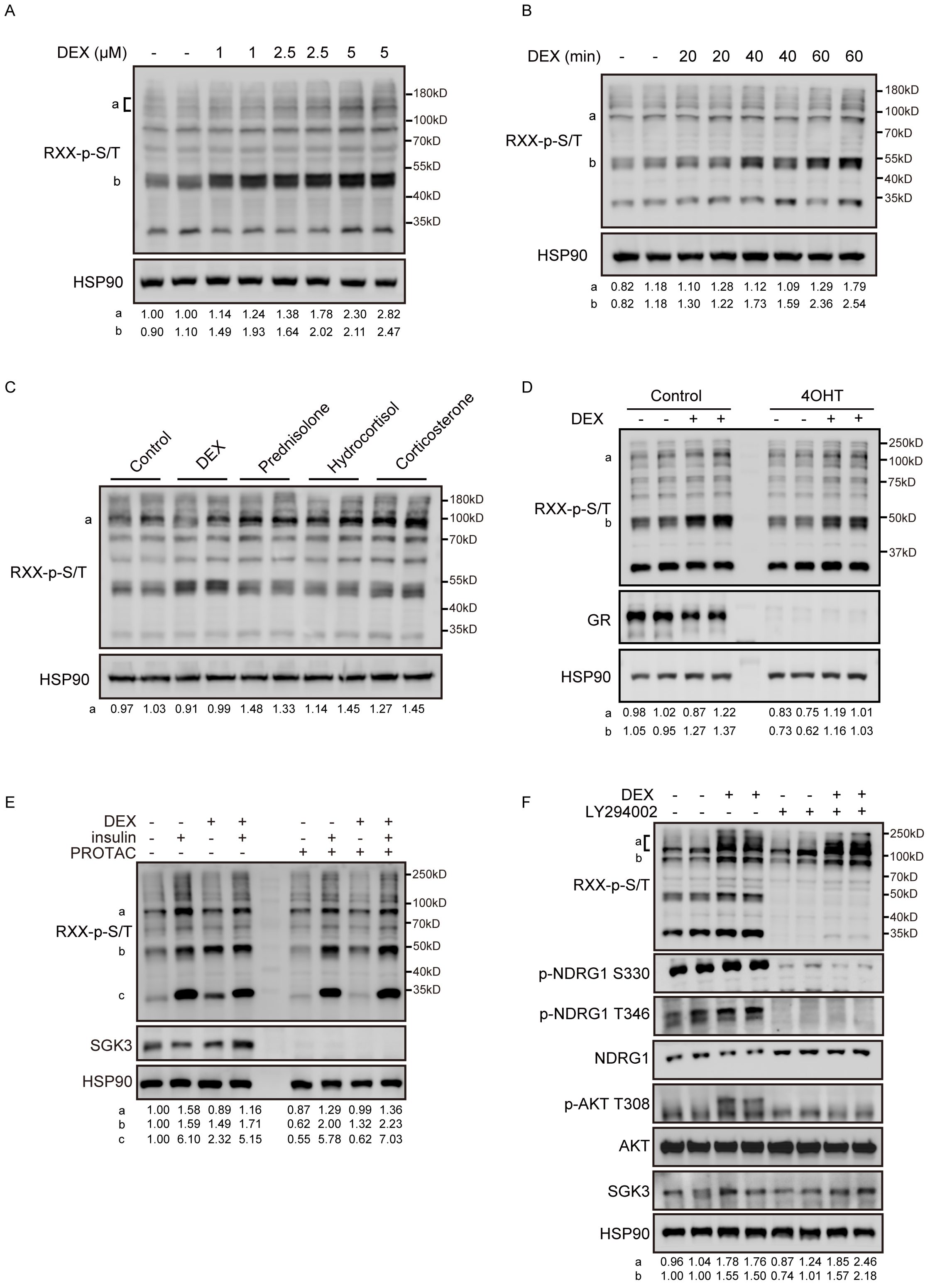

Supplemental Figure 2. Glucocorticoids induce protein phosphorylation.

(**A**) DEX increases RxxS/T phosphorylation in SVF cells cultured with CS-FBS

in a dose-dependent manner. Relative band intensities are quantified below (normalized to HSP90). (**B**) Time course of DEX-induced RxxS/T phosphorylation in SVF cells cultured with CS-FBS. Relative band intensities are shown below (normalized to HSP90). (**C**) Phosphorylation of RxxS/T peptides induced by different glucocorticoids in SVF cells cultured with CS-FBS. Relative band intensities are shown below (normalized to HSP90). **(D)** DEX-induced RxxS/T phosphorylation in control and 4OHT induced GR-deficient cells. Relative band intensities are shown below (normalized to HSP90). **(E)** DEX or insulin-induced phosphorylation of RxxS/T peptides in SGK3 deficient cells cultured with CS-FBS. The relative quantitative of letter labeled bands were indicated below the gel (normalized to HSP90). **(F)** DEX-induced phosphorylation of RxxS/T peptides, phosphorylation of NDRG1 at serine 330 or threonine 346 and phosphorylation of AKT at threonine 308 in SVF cells. The relative quantitative of letter labeled bands were indicated below the gel (normalized to HSP90). Identical sample aliquots were loaded on separate gels for Western blotting analysis in Figures A-F.

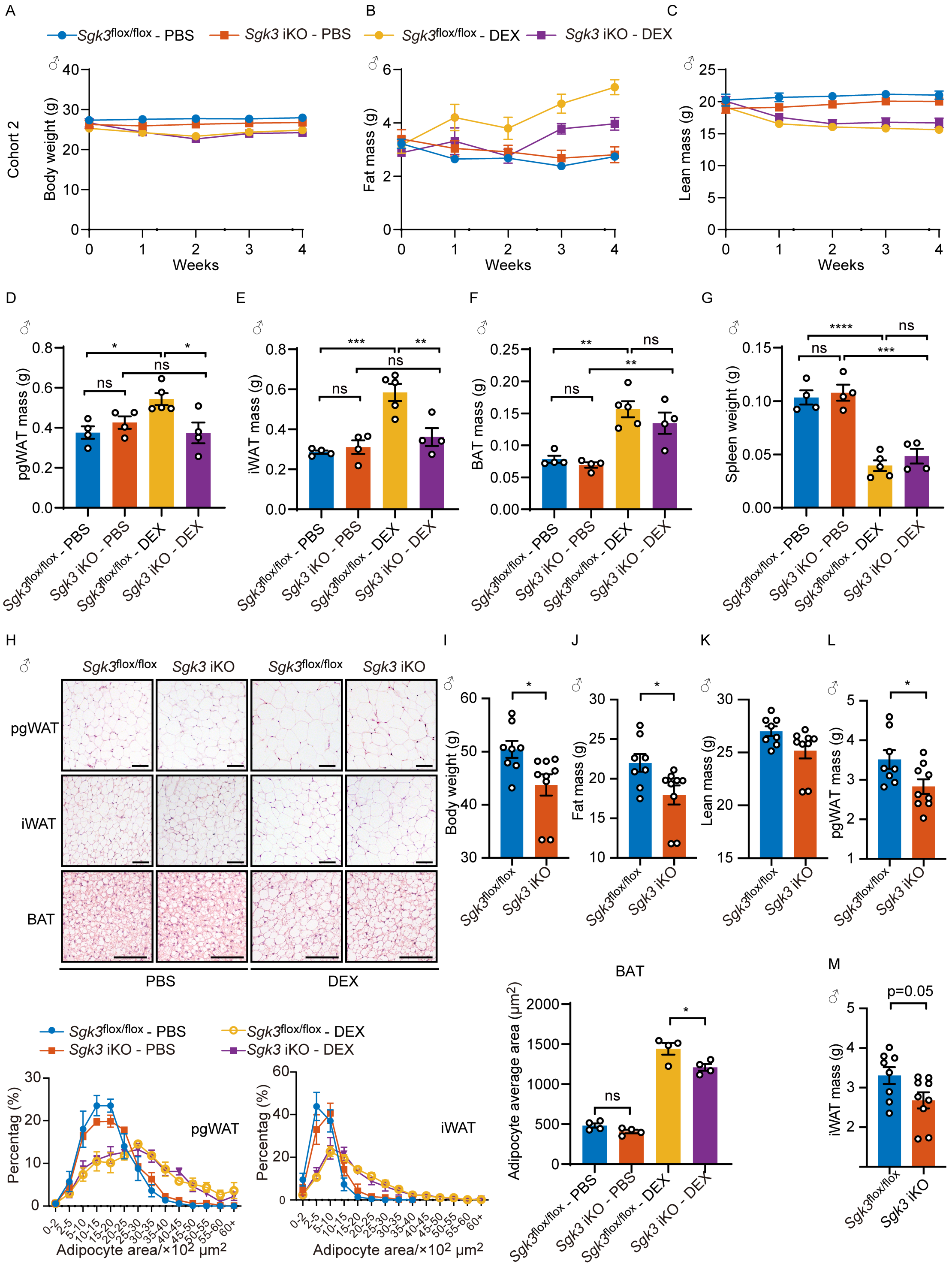

Supplemental Figure 3. *Sgk3*-deficient mice are protected from DEX- or HFD-induced obesity.

(**A**-**G**) Body weight (**A**),fat mass (**B**), lean mass (**C**) and tissue weights (**D**-**G**) in

PBS- or DEX-treated *Sgk3*$^{flox/flox}$ and *Sgk3* iKO male mice (cohort 2) after 4 weeks of treatment. (**D**) pgWAT, (**E**) iWAT, (**F**) BAT and (**G**) spleen weights are shown. *n* = 4 for *Sgk3*$^{flox/flox}$ mice (Veh), *Sgk3* iKO mice (Veh) and *Sgk3* iKO (DEX) mice, *n* = 5 for *Sgk3*$^{flox/flox}$ mice (DEX). (**H**) Representative images of H&E staining of pgWAT, iWAT and BAT of PBS- or DEX-treated *Sgk3*$^{flox/flox}$ and *Sgk3* iKO male mice over 28 days. Scale bar: 100 μm. Related quantifications of adipocyte area of pgWAT and iWAT. *n* = 4 mice per group (>200 adipocytes are quantified for each mouse). Related quantification of adipocyte area of BAT. *n* = 4 mice per group. Relative adipocyte size in BAT was calculated by dividing the adipose tissue area by the number of nuclei within the same region, providing a normalized measure of adipocyte area. (**I**-**M**) Body weight (**I**), fat mass (**J**), lean mass (**K**), pgWAT weight (**L**) and iWAT weight (**M**) of HFD-fed *Sgk3*$^{flox/flox}$ and *Sgk3* iKO male mice over 10 weeks. *n* = 8 for *Sgk3*$^{flox/flox}$, n=9 for *Sgk3* iKO. Data in **A**-**C**, were represented as mean ± SEM. Data in **D**-**H** were represented as mean ± SEM, and One-way ANOVA was used for statistical analysis. Data in **I**-**M** were represented as mean ± SEM, and unpaired two-tailed T-test was used for statistical analysis. "ns" indicates no significance, **P* < 0.05, ***P* < 0.01, ****P* < 0. 001, *****P* < 0. 0001.

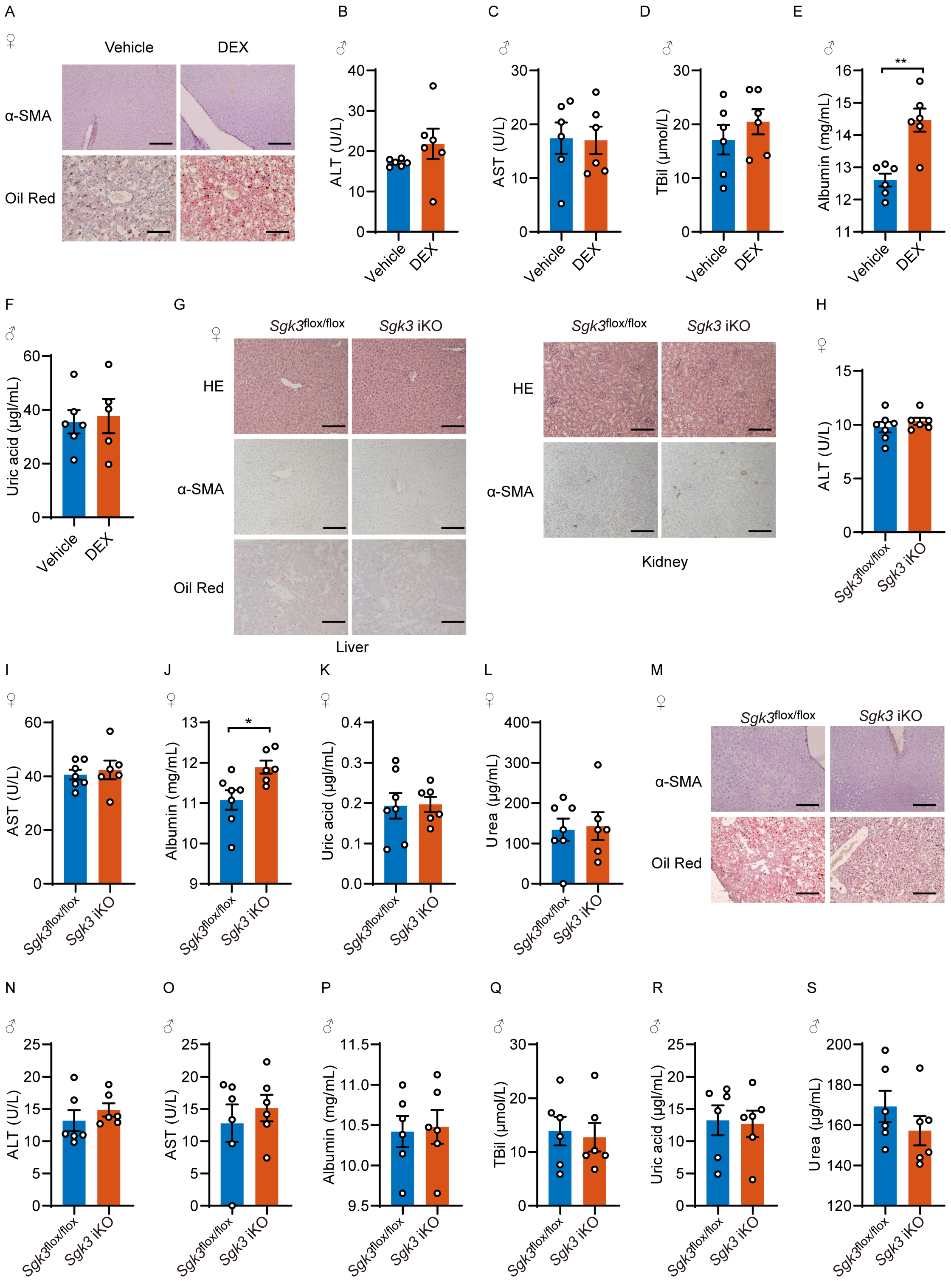

Supplemental Figure 4. The absence of SGK3 does not compromise normal hepatic or renal function, regardless of DEX treatment status.

(**A**) Representative images of liver α-SMA IHC staining and Oil red O staining

of DEX treated female mice over 28 days. (**B-F**) Serum ALT (**B**), AST (**C**), TBiL (**D**), albumin (**E**), and uric acid (**F**) of PBS- or DEX-treated male mice over 28 days. *n* = 6 for each group. (**G**) Representative images of liver H&E, α-SMA IHC, Oil red O staining and kidney H&E, α-SMA IHC of PBS-treated 10-week-old *Sgk3* iKO female mice. (**H-L**) Serum ALT (**H**), AST (**I**), albumin (**J**), uric acid (**K**), and urea (**L**) of PBS-treated 10-week-old *Sgk3* iKO female mice. *n* = 7 for *Sgk3*$^{flox/flox}$ mice, and *n* = 6 for *Sgk3* iKO mice. (**M**) Representative images of liver α-SMA IHC staining and liver Oil red O staining of DEX-treated *Sgk3* iKO female mice over 28 days. (**N-S**) Serum ALT (**N**), AST (**O**), albumin (**P**), TBil (**Q**), uric acid (**R**), and urea (**S**) of DEX treated *Sgk3* iKO male mice over 28 days. *n* = 6 for each group. Data in **B**-**F, H**-**L,** and **N**-**S** were represented as mean ± SEM, and unpaired two-tailed T-test was used for statistical analysis. *P < 0.05, **P < 0. 01.

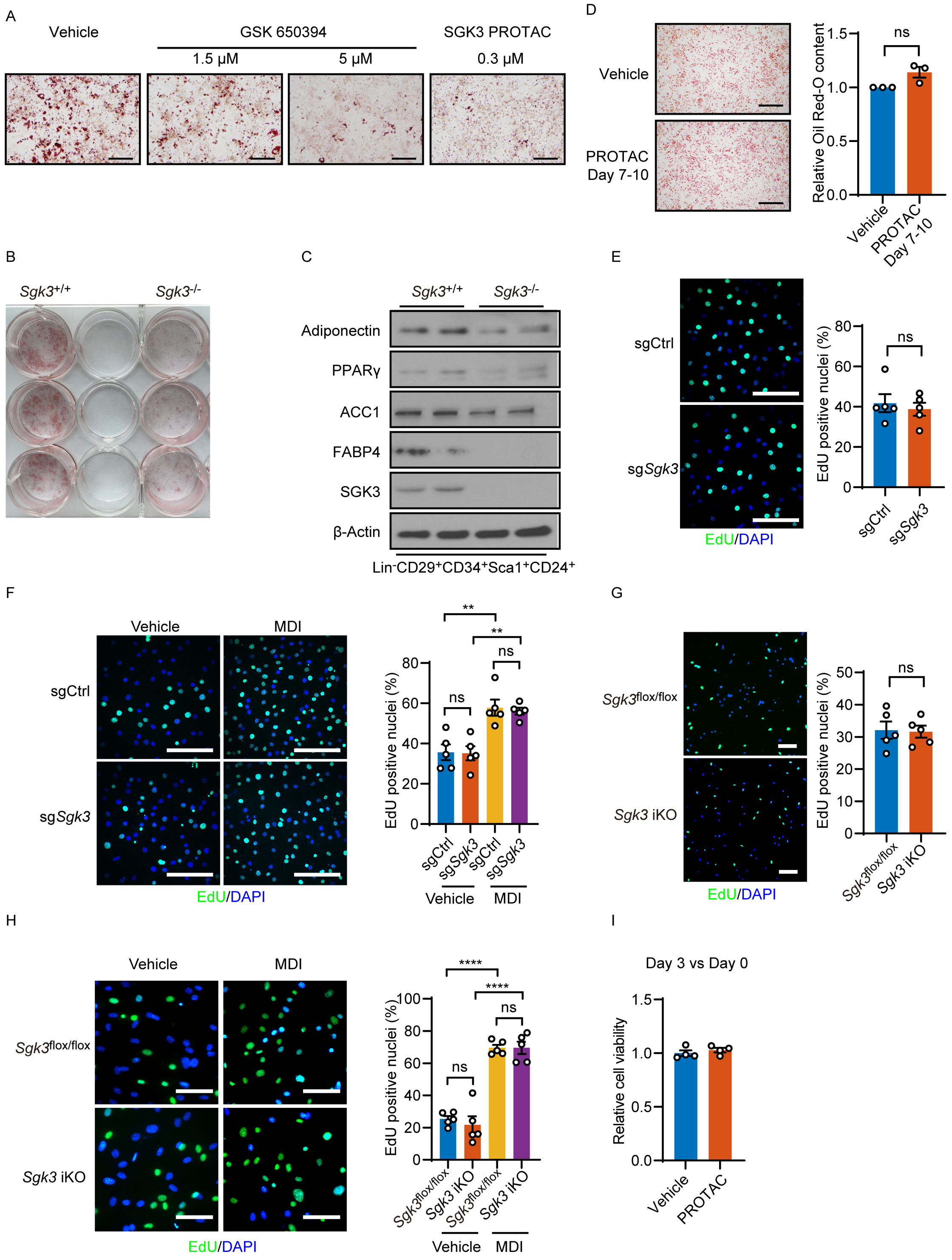

Supplemental Figure 5. SGK3 regulates adipogenesis independently of cell cycle regulation.

(**A**) Representative Oil Red O staining images of differentiated SVF cells treated

with GSK 650394 or SGK3-PROTAC at day 7 post-induction, Scale bar: 200 μm. (**B**) Representative images of Oil red O staining of differentiated primary SVF cells from *Sgk3*$^{+/+}$ and *Sgk3*$^{-/-}$ on day 7. (**C**) The expression of Adiponectin, PPARγ, ACC1, FABP4 and SGK3 in differentiated primary preadipocytes from *Sgk3*$^{+/+}$ and *Sgk3*$^{-/-}$ mice on day 7. (**D**) Representative images of Oil red O staining and related quantifications of mature adipocyte treated with SGK3 PROTAC from day 7 to day 10. Scale bar: 4 mm. *n* = 3 independent assays. (**E**) Representative images of EdU staining and related quantifications of proliferating *Sgk3* KO preadipocytes. Scale bar: 50 μm. *n* = 5 different microscopic fields. (**F**) Representative images of EdU staining and related quantifications of differentiating *Sgk3* KO preadipocytes. Scale bar: 50 μm. *n* = 5 different microscopic fields. (**G**) Representative images of EdU staining and related quantifications of proliferating *Sgk3* KO primary SVF cells. Scale bar: 50 μm. *n* = 5 different microscopic fields. (**H**) Representative images of EdU staining and related quantifications of differentiating *Sgk3* KO primary SVF cells. Scale bar: 50 μm. *n* = 5 different microscopic fields. (**I**) MTT assay of SGK3 PROTAC treated preadipocytes. *n* = 4 samples from different culture wells. Data in **D** were represented as mean ± SEM, and paired two tailed T-test was used for statistical analysis. Data in **E**, **G** and **I** were represented as mean ± SEM, and unpaired two tailed T-test was used for statistical analysis. Data in **F** and **H** were represented as mean ± SEM, and One-way ANOVA was used for statistical analysis. "ns" indicates no significance, $^{**}P < 0.01$, $^{****}P < 0.0001$.

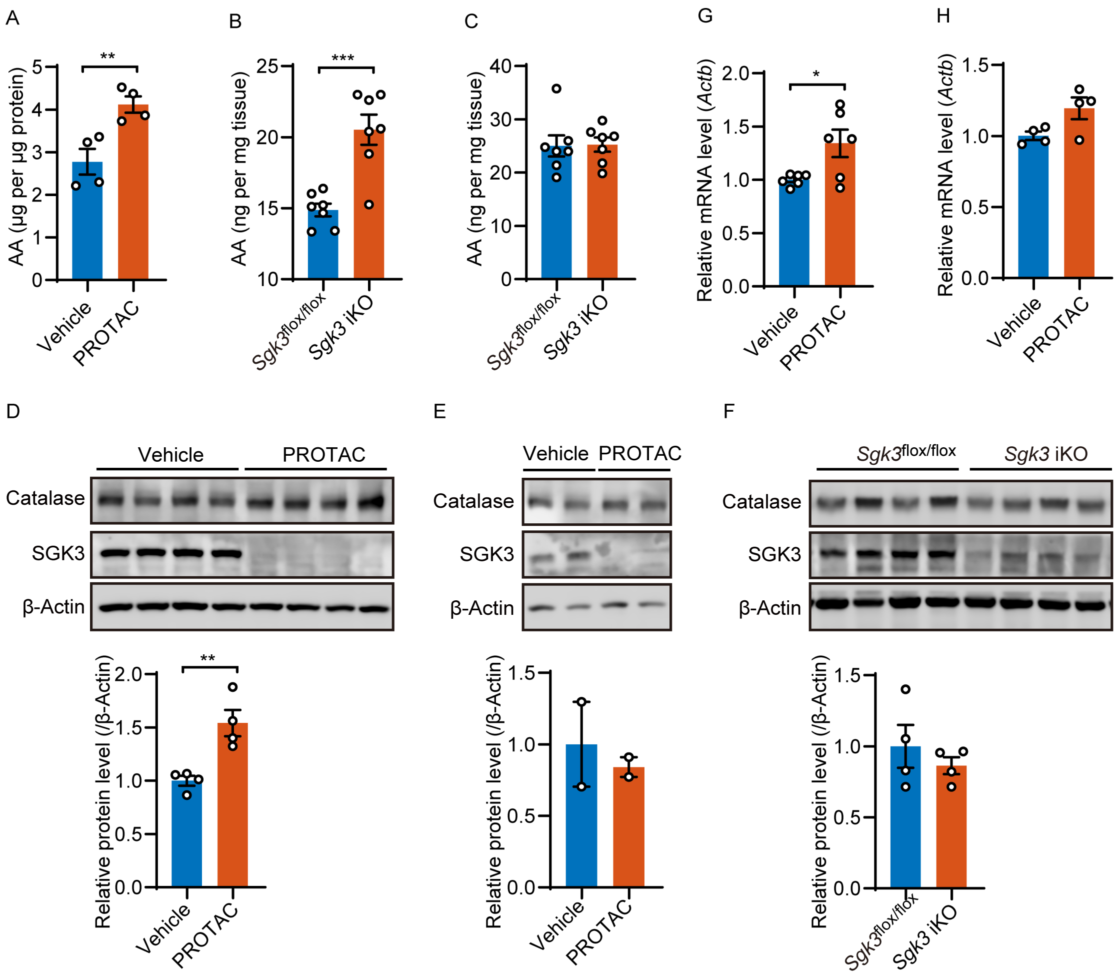

Supplemental Figure 6. SGK3 deficiency does not alter catalase activity or arachidonic acid levels in either SVF cells or adipose tissue.

(**A-C**) Arachidonic acid content in SGK3 PROTAC treated SVF cells (**A**), iWAT (**B**) and pgWAT (**C**) of DEX treated *Sgk3* iKO female mice. *n* = 4 for different SVF culture wells, *n* = 7 for *Sgk3*$^{flox/flox}$ and *Sgk3* iKO mice. (**D-F**) Western blot and related quantifications of catalase in SGK3 PROTAC treated immortalized SVF (**D**), primary SVF (**E**), and pgWAT of *Sgk3* iKO mice (**F**). *n* = 2 for immortalized SVF, *n* = 4 for primary SVF samples from different culture wells, and *n* = 4 for pgWAT of *Sgk3* iKO mice. (**G-H**) mRNA level of *Catalase* in SGK3 PROTAC treated immortalized SVF (**G**) and primary SVF (**H**). *n* = 6 for immortalized SVF and *n* = 4 for primary SVF samples from different culture wells. Identical sample aliquots were loaded on separate gels for Western

blotting analysis in Figures D-F. Data in **A**-**H** were represented as mean ± SEM, and unpaired two tailed T-test was used for statistical analysis. **P* < 0. 05, ***P* < 0. 01, ****P*- < 0.001.

A

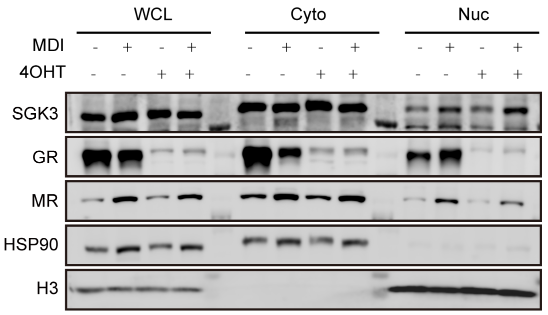

B

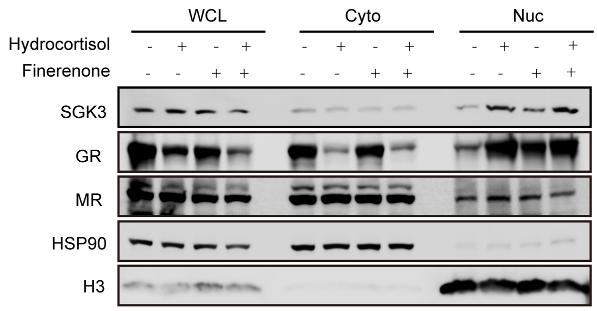

C

MS identified proteins of nuclear import relative protein

Accession	Protein name	net unique peptides Day 0	net unique peptides Day 3
Q8BFY9	Transportin-1	1	-1
Q6P2B1	Transportin-3	2	0
Q8VI75	Importin-4	0	1
Q8BKC5	Importin-5	1	1
Q91YE6	Importin-9	1	-1
Q8K0C1	Importin-13	0	1
P52293	Importin subunit alpha-1	0	0
O35343	Importin subunit alpha-3	0	2
O35344	Importin subunit alpha-4	0	1
Q60960	Importin subunit alpha-5	2	2
O35345	Importin subunit alpha-7	0	0
P70168	Importin subunit beta-1	5	2
Q6P5F9	Exportin-1	4	10
Q9ERK4	Exportin-2	1	2
Q924C1	Exportin-5	0	2
Q9CRT8	Exportin-T	0	3

D

MS-identified proteins of SWI/SNF complex

Accession	Protein name	net unique peptides Day 0	net unique peptides Day 3
P97496	SMARCC1	1	5
Q99JR8	SMARCD2	2	6
A2BH40	ARID1A	1	2
O54941	SMARCE1	3	3
Q3TKT4	SMARCA4(BRG1)	7	16
Q6P9Z1	SMARCD3	2	4
Q6PDG5	SMARCC2	5	9
Q9Z0H3	SMARCB1	1	4
Q9Z2N8	ACTL6A	2	3
E9Q4N7	ARID1B	0	0
Q61103	SMARCD1	0	2
Q61466	SMARCA2	0	1
Q6DIC0	DPF2	0	0

Supplemental Figure 7. SGK3 translocates to the nucleus independent of GR and MR and interacts with the BAF complex.

(**A**) Subcellular localization of SGK3 in cytosolic and nuclear fractions of control

and GR-deficient SVF cells treated with MDI during preadipocyte differentiation (day 2). (**B**) SGK3 localization in cytosolic and nuclear fractions of SVF cells treated with hydrocortisol and/or finerenone during preadipocyte differentiation (day 2). (**C**) Unique peptides number of proteins relative to cytosol-nuclear transporter identified by SGK3-miniTurbo labeling. (**D**) Unique peptides number of proteins components of BAF complex by SGK3-miniTurbo labeling. Identical sample aliquots were loaded on separate gels for Western blotting analysis in Figures A and B.

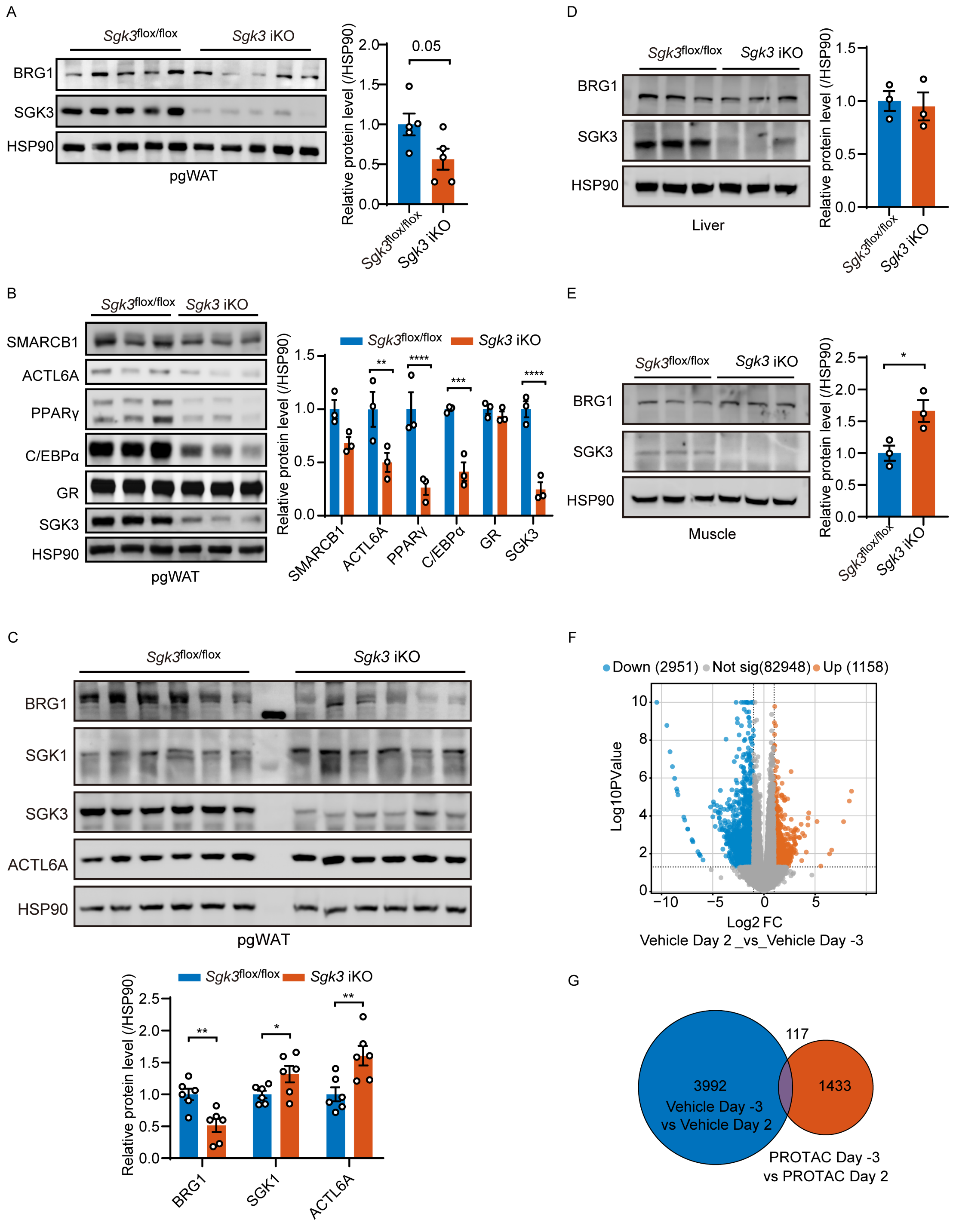

Supplemental Figure 8. Deficiency of SGK3 downregulates BRG1 protein level in white adipose tissue and impairs chromatin remodeling during preadipocyte differentiation.

(**A** and **B**) Western blot and related quantifications of SGK3 and BRG1 (**A**), SMARCB1, ACTL6A, PPARγ, C/EBPα and GR (**B**) in pgWAT of DEX-treated *Sgk3*$^{flox/flox}$ and *Sgk3* iKO male mice for 32 days. Each line represents 1 mouse. *n* = 5 for A, and *n* = 3 for B. (**C**) Western blot and related quantifications of BRG1, SGK1, and ACTL6A in pgWAT of PBS-treated *Sgk3*$^{flox/flox}$ and *Sgk3* iKO male mice for 32 days. Each line represents 1 mouse. *n* = 6. (**D** and **E**) Western blot and related quantifications of BRG1 in liver (**D**) and muscle (**E**) of DEX-treated *Sgk3*$^{flox/flox}$ and *Sgk3* iKO male mice for 32 days. Each line represents 1 mouse. *n* = 3. (**F**) Volcano plot of BRG1 differential occupied regions in Day 2 vs Day -3. (**G**) Venn plot of BRG1 differential occupied regions in vehicle or SGK3 PROTAC treated cells. Identical sample aliquots were loaded on separate gels for Western blotting analysis in Figures B and C.

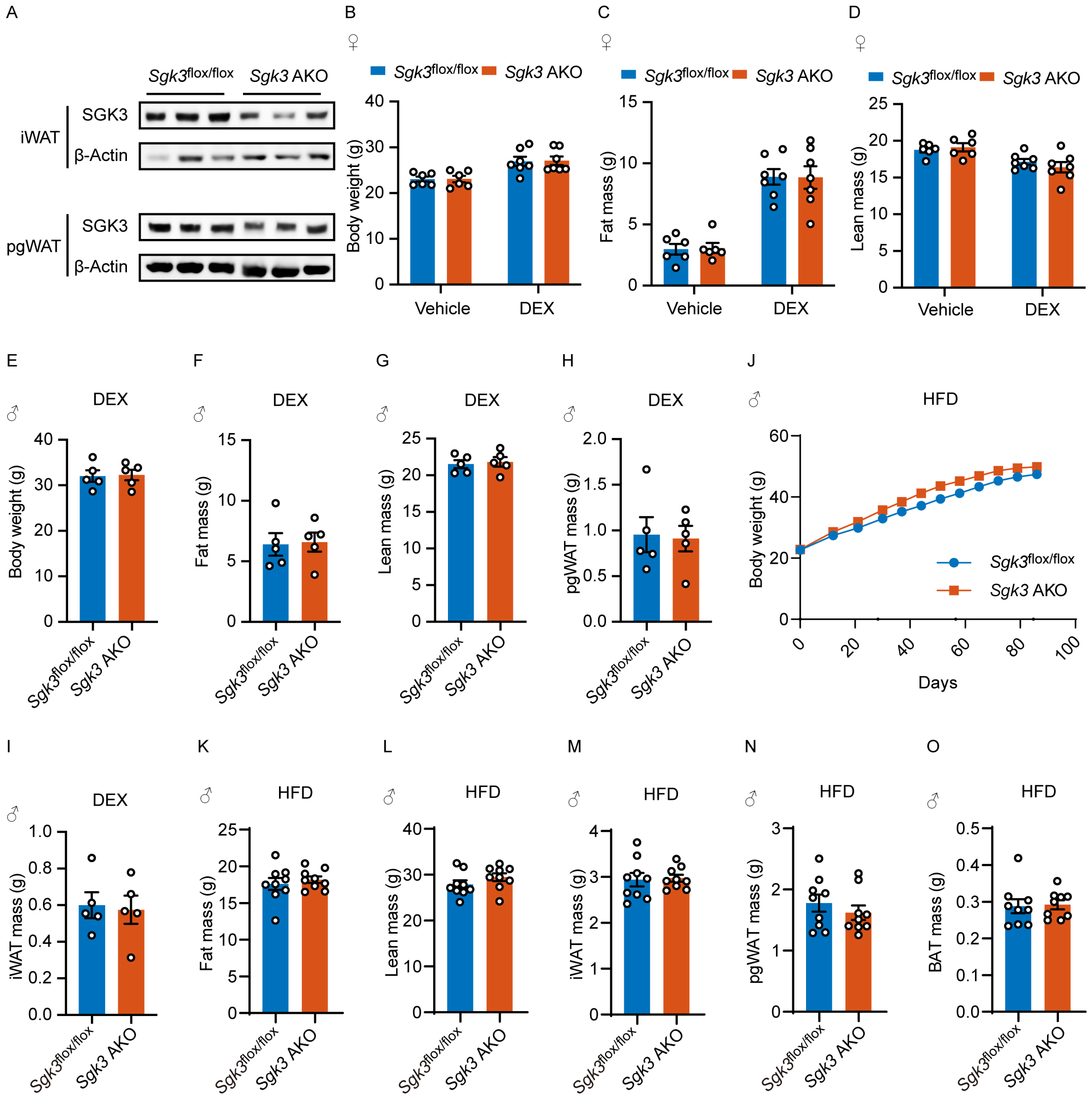

Supplemental Figure 9. Knock out of *Sgk3* in mature adipocytes does not protect from DEX- or HFD-induced obesity.

(**A**) Representative western blot of SGK3 of pgWAT and iWAT in *Sgk3*$^{flox/flox}$ and *Sgk3* AKO male mice. (**B**-**D**) Body weight (**B**), fat mass (**C**), and lean mass (**D**) of vehicle- or DEX-treated *Sgk3*$^{flox/flox}$ and *Sgk3* AKO female mice over 28 days. *n* = 6 for *Sgk3*$^{flox/flox}$ (Veh), *n* = 6 for *Sgk3* AKO (Veh), *n* = 7 for *Sgk3*$^{flox/flox}$ (DEX),

n = 7 for *Sgk3* AKO (DEX). (**E**-**I**) Body weight (**E**), fat mass (**F**), lean mass (**G**), pgWAT (**H**) and iWAT (**I**) of DEX-treated *Sgk3*$^{flox/flox}$ and *Sgk3* AKO male mice over 28 days. *n* = 5 per group. (**J**) Body weight of HFD-fed *Sgk3*$^{flox/flox}$ and *Sgk3* AKO male mice over 3 months. *n* = 12 for each group. (**K**-**O**) Fat mass (**K**), lean mass (**L**), iWAT mass (**M**), pgWAT mass (**N**) and BAT mass (**O**) of HFD-fed *Sgk3*$^{flox/flox}$ and *Sgk3* AKO male mice over 3.5 months. *n* = 9 for each group. Data in **B**-**D** were represented as mean ± SEM, and One-way ANOVA was used for statistical analysis. Data in **E**-**I**, **K**-**O** were represented as mean ± SEM, and unpaired two tailed T-test was used for statistical analysis. Data in **J** were represented as mean ± SEM.

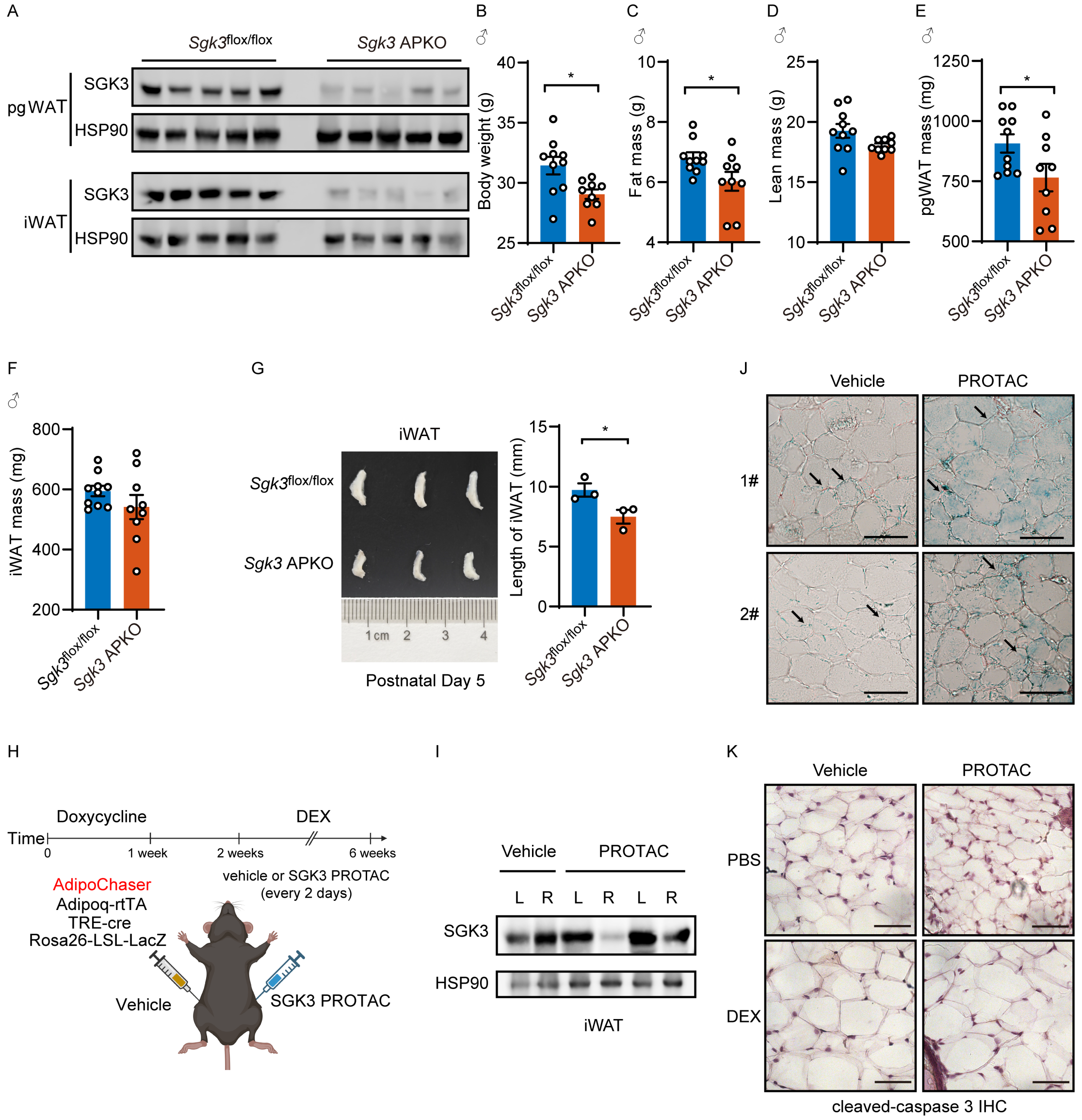

Supplemental Figure 10. Knock out of *Sgk3* in preadipocytes protects from DEX-induced obesity.

(**A**) Western blot of SGK3 of pgWAT and iWAT of *Sgk3*$^{flox/flox}$ and *Sgk3* APKO (adipogenic progenitor cell-specific knockout) male mice. Each line represents 1 mouse. (**B**-**F**) Body weight (**B**), Fat mass (**C**), Lean mass (**D**), pgWAT weight (**E**) and iWAT weight (**F**) of DEX-treated *Sgk3*$^{flox/flox}$ and *Sgk3* APKO male mice

over 35 days. *n* = 10 for *Sgk3*$^{flox/flox}$ mice, *n* = 9 for *Sgk3* APKO mice. (**G**) Images of iWAT of *Sgk3*$^{flox/flox}$ and *Sgk3* APKO male mice at postnatal day 5. The length of iWAT in each group was measured for statistical analysis. *n* = 3 for each group. (**H**) Experimental workflow to examine the function of SGK3 in adipogenesis *in vivo* using AdipoChaser-*LacZ* mice. (**I**) Representative western blot of SGK3 in PROTAC-treated iWAT tissue. (**J**) Representative images of *in situ* β-gal staining of iWAT sections. Scale bar: 200 μm. Blue adipocytes represent old LacZ-positive cells, indicated by arrowheads. (**K**) Immunohistochemical analysis of Cleaved caspase-3 in iWAT from the indicated groups of AdipoChaser-*mTmG* mice. Scale bar: 50 μm. Data in **B**-**F** and **G** are represented as mean ± SEM, and unpaired two-tailed T-test was used for statistical analysis. *$P < 0.05$.

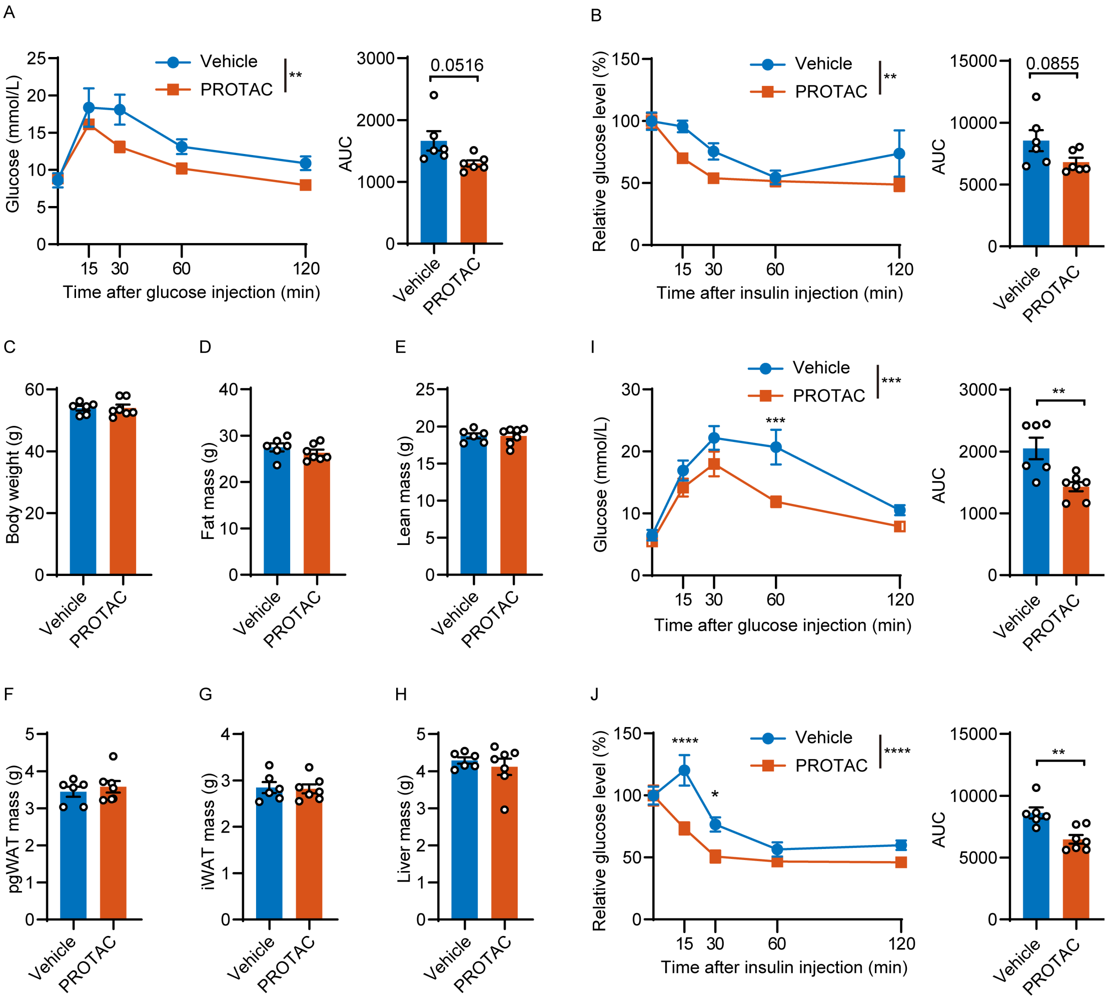

K

Genotype	Specificity	Phenotype (glucocorticoid administration)
Sgk3-/-	Whole body	Ameliorate excessive adipose tissue accumulation.
CAG-Cre^{esr1}, *Sgk3*$^{flox/flox}$	Induced whole body	Ameliorate excessive adipose tissue accumulation.
Adipoq-Cre, *Sgk3*$^{flox/flox}$	Mature adipocyte	Failed to ameliorate excessive adipose tissue accumulation.
Pdgfra-Cre, *Sgk3*$^{flox/flox}$	Adipocyte progenitor and thereafter differentiated adipocyte	Ameliorate excessive adipose tissue accumulation.

L

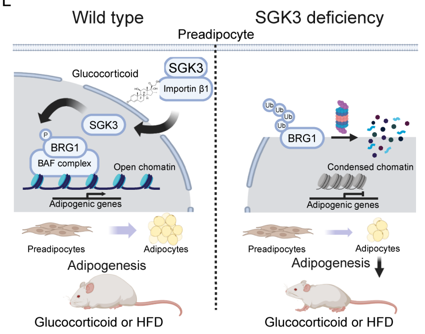

Supplemental Figure 11. SGK3 PROTAC ameliorated glucose homeostasis in obese mice but not obesity.

(**A-B**) Glucose tolerance tests (**A**) and insulin tolerance tests (**B**) were

performed on mice fed a HFD for 8 weeks and then treated with either vehicle or SGK3 PROTAC for 6 weeks. *n* = 6 for each group. (**C**-**J**) 10-week-old male *ob/ob* mice were treated with vehicle or SGK3 PROTAC for 6 weeks. Body weight (**C**), fat mass (**D**), lean mass (**E**), pgWAT weight (**F**), iWAT weight (**G**), liver weight (**H**), glucose tolerance (**I**), and insulin tolerance (**J**) were measured in mice from each treatment group. *n* = 6 for vehicle treated mice, and *n* = 7 for SGK3-PROTAC treated mice. (**K**) Description of SGK3 function in glucocorticoid induced obesity *in vivo*. (**L**) Proposed mechanism of glucocorticoid-activated SGK3 in regulating preadipocytes differentiation. Data in **A**, **B**, **I**, **J** are represented as mean ± SEM, and Two-way ANOVA was used for GTT and ITT statistical analysis, unpaired two-tailed T-test was used for AUC statistical analysis **C**-**H** are represented as mean ± SEM, and unpaired two-tailed T-test was used for statistical analysis. $^{**}P < 0.01$, $^{***}P < 0.001$, $^{****}P < 0.0001$.

Supplemental Table 1. Top 100 upregulated phosphorylation sites in DEX induced preadipocyte.

Protein accession	Position	Amino acid	Protein name	Modified sequence
Q80XQ2	578	S	TBC1D5	_EFTGS[Phospho (STY)]PPPSATK_
Q8BKX1	456	T	BAIAP2	_SSST[Phospho (STY)]GNLLDKDDLALPPPDYGTSSR_
Q6P8I4	119	S	PCNP	_TLSVAAAFNEDEDS[Phospho (STY)]EPEEMPPEAK_
E9Q7G0	270	S	NUMA1	_QAASS[Phospho (STY)]QEPSELEELR_
Q61183	545	S	PAPOLA	_TS[Phospho (STY)]PLNSSGSSQGR_
Q63918	25	S	CAVIN2	_FQHPNTDMLQEKPSS[Phospho (STY)]PSPMPSSTPSPSLNLGSTEEAIR_
Q7TQI3	16	S	OTUB1	_QEPLGS[Phospho (STY)]DSEGVNC[Carbamidomethyl (C)]LAYDEAIMAQQDR_
Q76KJ5	160	T	POLR1G	_FSAFGGSPPVT[Phospho (STY)]GPGSASALRSPTSGK_
Q8BTI8	1066	S	SRRM2	_S[Phospho (STY)]SS[Phospho (STY)]PVTELTAR_
P70398	1600	S	USP9X	_NGILAIEGTGSDVDDDMS[Phospho (STY)]GDEKQDNESNVDPR_
Q3TKT4	3	T	SMARCA4	_[Acetyl (Protein N-term)]ST[Phospho (STY)]PDPPLGGTPR_
Q8C080	109	T	SNX16	_EAEEQHPEAVNWEDRPST[Phospho (STY)]PTILGYEVMEER_
Q7TQE6	332	S	MACO1	_NASGVVNSS[Phospho (STY)]PR_
Q0VGY8	1461	T	TANC1	_DHFPIEEAEEEDT[Phospho (STY)]SS[Phospho (STY)]QEESISPTPR_
Q8BHN5	199	S	RBM45	_VSGS[Phospho (STY)]PEQDDYSSGR_
Q8BQZ4	710	S	RALGAPB	_TNS[Phospho (STY)]GISSASGGSTEPTTPDSERPAQALLR_
Q3U9G9	71	S	LBR	_SGSISSS[Phospho (STY)]PSR_
Q9WTQ5	1059	S	AKAP12	_VEEVEEDS[Phospho (STY)]EVLATEK_
P97452	112	S	BOP1	_TEEAGALAQDEYEEDS[Phospho (STY)]S[Phospho (STY)]DEEDIR_
P97452	113	S	BOP1	_TEEAGALAQDEYEEDS[Phospho (STY)]S[Phospho (STY)]DEEDIR_
P14873	1321	S	MAP1B	_TLEVVSPSQS[Phospho (STY)]VTGSAGHTPYYQSPTDEK_
Q8VCW4	541	Y	UNC93B1	_Y[Phospho (STY)]LEEDNSDES[Phospho (STY)]DMEGEQGQGDC[Carbamidomethyl (C)]AEDEAPQAGPLGAEPAGPC[Carbamidomethyl (C)]R_
Q6ZPZ3	1115	S	ZC3H4	_AAKPC[Carbamidomethyl (C)]PTEAS[Phospho (STY)]PPAASPSGDSS[Phospho (STY)]PPATAPYDPR_
Q6NZR2	32	S	MSANTD2	_MEVLSPASPGDLS[Phospho (STY)]DGNPSLSDPSTPR_
Q8VC03	285	Y	EML3	_SGEVVYFIAC[Carbamidomethyl (C)]VVVLY[Phospho (STY)]RPGGGPGGPGGGGQR_
Q8K0L9	225	S	ZBTB20	_GTPESGTSGQSS[Phospho (STY)]DTESGYLQSHPQHSVDR_
Q3U214	157	S	MAST3	_SS[Phospho (STY)]ESVVDEDGGR_
O35691	407	T	PNN	_HVIAEQEVMET[Phospho (STY)]NQVESIEPSENETSK_
Q8K3X4	315	S	IRF2BPL	_RPGS[Phospho (STY)]VS[Phospho (STY)]STDQER_
E9Q137	262	Y	TEX264	_SEHSY[Phospho (STY)]SESGASGSSFEELDLEGEGPLGEPR_
Q7TPS5	284	S	C2CD5	_EIPFNEDPNPNTHSSGPS[Phospho (STY)]TPLK_
Q8BG67	222	S	EFR3A	_LGPPSSPS[Phospho (STY)]AADKEENPAVLAESC[Carbamidomethyl (C)]FR_

Q8C2B3	131	S	HDAC7	_TVHPS[Phospho (STY)]SPSIPYR_
Q8C8U0	346	S	PPFIBP1	_S[Phospho (STY)]TSS[Phospho (STY)]TPGMGSPSR_
A2AAE1	2727	S	BLTP1	_SS[Phospho (STY)]ETFGPAGVR_
O08750	285	S	NFIL3	_S[Phospho (STY)]SDGEDEQQVPK_
Q9WTQ5	272	T	AKAP12	_EPTKPLESPT[Phospho (STY)]SPVSNETTSSFK_
Q69Z38	403	T	PEAK1	_DIAETESNFESPPGNNEEKDESLT[Phospho (STY)]SK_
Q62059	2586	S	VCAN	_LESHGS[Phospho (STY)]S[Phospho (STY)]EESLQVQEK_
P70280	168	S	VAMP7	_TENLVDSS[Phospho (STY)]VTFK_
P14873	1384	S	MAP1B	_DENERAS[Phospho (STY)]LS[Phospho (STY)]PMDEPVPDSES[Phospho (STY)]PVEK_
P23198	173	T	CBX3	_LT[Phospho (STY)]WHSC[Carbamidomethyl (C)]PEDEAQ_
Q6ZQF7	122	T	JADE2	_GPPTQMSPDSPT[Phospho (STY)]LGEGAHPDWPGGSR_
Q05A36	225	T	MEX3C	_SVNT[Phospho (STY)]TEC[Carbamidomethyl (C)]VPVPSSEHVAEIVGR_
E9Q309	1930	S	CEP350	_GIASEEGSPMPSYS[Phospho (STY)]PR_
Q9Z1Q9	524	S	VARS1	_VQGS[Phospho (STY)]DSDEEVVVATTR_
Q6DFV3	63	S	ARHGAP21	_TS[Phospho (STY)]QGFGFTLR_
Q80V24	58	S	VGLL4	_TGPPPIS[Phospho (STY)]PSK_
Q8BVK9	177	S	SP110	_ATAQPIIEILDEQPSPS[Phospho (STY)]PR_
Q02956	395	Y	PRKCZ	_LTDY[Phospho (STY)]GMC[Carbamidomethyl (C)]KEGLGPGDTTSTFC[Carbamidomethyl (C)]GTPNYIAPEILR_
Q60875	940	S	ARHGEF2	_LQDSS[Phospho (STY)]DPDTGSEEEVSSRLS[Phospho (STY)]PPHS[Phospho (STY)]PR_
Q3UMF0	300	S	COBLL1	_EQTAS[Phospho (STY)]APATPLVSK_
Q9D0S9	52	S	HINT2	_AAPGGAS[Phospho (STY)]PTIFSR_
Q91ZX7	4525	T	LRP1	_HSLAST[Phospho (STY)]DEK_
O54988	354	S	SLK	_ASSDLSIASS[Phospho (STY)]EEDKLS[Phospho (STY)]QNAC[Carbamidomethyl (C)]ILESVSER_
Q8R2M2	352	S	DNTTIP2	_KPTFQLS[Phospho (STY)]SPDRK_
O88895	414	Y	HDAC3	_GPEENYSRPEAPNEFY[Phospho (STY)]DGDHDNDKESDVEI_
Q00560	757	Y	IL6ST	_PSISSNEENESAQSTASTVQY[Phospho (STY)]STVVHSGYR_
Q9WTQ5	704	S	AKAP12	_AEEAS[Phospho (STY)]KDKEADALPASTQEQDQAHGSSSPEPAGSPSEGEGVSTWESFK_
Q924A2	2200	T	CIC	_GEPPTPPSPAPAT[Phospho (STY)]GPSGSSSGSSEGSSGR_
Q8BHB4	249	S	WDR3	_EC[Carbamidomethyl (C)]PGIQDTPES[Phospho (STY)]EDST[Phospho (STY)]LEADDEKSEDR_
Q8VH51	100	S	RBM39	_YRS[Phospho (STY)]PYS[Phospho (STY)]GPK_
D0QMC3	176	T	MNDAL	_TEEDC[Carbamidomethyl (C)]LQNSPKPPPT[Phospho (STY)]SPSSSSNK_
Q3UYC0	113	T	PPM1H	_AGTITST[Phospho (STY)]PNR_
Q9ERV1	139	S	MKRN2	_VNNPGGC[Carbamidomethyl (C)]SDPQTS[Phospho (STY)]PEMKPHSYLDAIR_
Q9R020	280	S	ZRANB2	_SYSSSSSS[Phospho (STY)]PER_
Q80X90	2497	S	FLNB	_SS[Phospho (STY)]TETC[Carbamidomethyl (C)]YSAIPK_
P51949	279	S	MNAT1	_AAS[Phospho (STY)]PQDLAGGYTSSLAC[Carbamidomethyl (C)]HR_

Q3TAA7	456	T	STK11IP	_TPALST[Phospho (STY)]PPLDVQNLETVC[Carbamidomethyl (C)]SPPAIEDDTK_
Q91W98	291	S	SLC15A4	_SGEGLGVFQQS[Phospho (STY)]SK_
O54834	758	S	ARHGAP6	_DPGMTGS[Phospho (STY)]YGDIFESSSLRPR_
P10854	39	S	H2BC14	_KESYS[Phospho (STY)]VYVYK_
Q64475	39	S	H2BC3	_KESYS[Phospho (STY)]VYVYK_
Q64478	39	S	H2BC9	_KESYS[Phospho (STY)]VYVYK_
Q64525	39	S	HIST2H2BB	_KESYS[Phospho (STY)]VYVYK_
Q6ZWY9	39	S	H2BC8	_KESYS[Phospho (STY)]VYVYK_
Q8CGP1	39	S	H2BC12	_KESYS[Phospho (STY)]VYVYK_
Q8CGP2	39	S	HIST1H2BP	_KESYS[Phospho (STY)]VYVYK_
Q80TI0	56	T	GRAMD1B	_SPST[Phospho (STY)]PEQGVQR_
P10853	39	S	H2BC15	_KESYS[Phospho (STY)]VYVYK_
Q91VT8	79	S	SMIM14	_GSS[Phospho (STY)]LPGKPSSPHSGQDPPAPPVD_
Q8K031	234	S	STARD8	_SLS[Phospho (STY)]IESLC[Carbamidomethyl (C)]PDEGR_
Q3B7Z2	196	S	OSBP	_MLAESDDSGDEES[Phospho (STY)]VSQTDKTELQSTLR_
Q8BFX3	712	S	KCTD3	_RS[Phospho (STY)]PGTEVR_
Q922T2	331	S	MFAP3	_SIGTDSQDSSHFS[Phospho (STY)]PPSDPASAEGSIHHR_
Q61026	217	S	NCOA2	_PLPDSEEEGHDS[Phospho (STY)]QEAHQK_
P07356	30	Y	ANXA2	_LSLEGDHSTPPSAYGSVKPY[Phospho (STY)]TNFDAER_
Q8VCZ8	273	S	RPUSD1	_TDPDPDPMSGGPRPC[Carbamidomethyl (C)]SPS[Phospho (STY)]TPQPR_
Q8R3B7	660	S	BRD8	_TEASPESMLSPS[Phospho (STY)]HGSNLIEDPLEAETQHK_
Q9R0Z9	861	Y	DLC1	_NSY[Phospho (STY)]TEQELKPLTLEALGHLNSDQPADYR_
Q9Z148	285	S	EHMT2	_KLNS[Phospho (STY)]GSLSEDLGSAGGSGDIILEK_
Q8C4Q9	115	S	TMEM154	_QEPSSQGSQS[Phospho (STY)]ALQTHELGGETLK_
Q99LJ0	559	S	CTTNBP2NL	_VSSPLS[Phospho (STY)]PLSPGIK_
Q8VC52	18	S	RBPMS2	_[Acetyl (Protein N-term)]SNLKPDVEHC[Carbamidomethyl (C)]TGAGTGS[Phospho (STY)]PLEEEVR_
P97393	1091	Y	ARHGAP5	_VPLAHPEDMDSSDNY[Phospho (STY)]VEPLDTIFK_
Q8CH09	732	S	SUGP2	_DSSDAAQDC[Carbamidomethyl (C)]LSEPAKPC[Carbamidomethyl (C)]PQPS[Phospho (STY)]SPGALGPSPR_
Q8BH43	139	Y	WASF2	_NSLPVPVLETYNSC[Carbamidomethyl (C)]DAPPPLNNLSPY[Phospho (STY)]R_
Q8CGF1	1042	T	ARHGAP29	_LLLLASSPT[Phospho (STY)]ER_
Q8K3A9	268	T	MEPCE	_GPHHQQQQASGGNDSNAAVLPTDPLT[Phospho (STY)]PSLHGEGATQQQQNR_
Q9D8M7	66	S	PHF10	_SC[Carbamidomethyl (C)]ETSSQDLSFS[Phospho (STY)]YYPAENLIEYK_

Supplemental Table 2. Top 100 downregulated phosphorylation sites in DEX induced preadipocyte.

Protein accession	Position	Amino acid	Protein name	Modified sequence
Q61183	544	T	PAPOLA	_T[Phospho (STY)]SPLNSSGSSQGR_
P14873	888	S	MAP1B	_GSAES[Phospho (STY)]PDEGITTTEGEGEC[Carbamidomethyl (C)]EQTPEELEPVEK_
Q7TQI3	18	S	OTUB1	_QEPLGSDS[Phospho (STY)]EGVNC[Carbamidomethyl (C)]LAYDEAIMAQQDR_
Q3UE17	4	S	MEX3D	_PGS[Phospho (STY)]TGQPDAGGAGTGTTAGDPGHPHPALAGAEDAAPRPPPEPDDAAAALR_
Q8C0T5	1624	S	SIPA1L1	_SLHGEFS[Phospho (STY)]ASDSSLTDIQETR_
Q640L3	185	T	CCPG1	_T[Phospho (STY)]VSIS[Phospho (STY)]ESEEPPLAEPEDEPSKEPSKR_
Q3UIA2	669	S	ARHGAP17	_S[Phospho (STY)]PSPPQQQQQQQQQQQQQQQQTPGMR_
A2A8U2	614	T	TMEM201	_KEDESS[Phospho (STY)]QS[Phospho (STY)]S[Phospho (STY)]T[Phospho (STY)]C[Carbamidomethyl (C)]VVDTTTK_
Q8CH77	1824	S	NAV1	_LYHLPPPSVGPHSTAS[Phospho (STY)]PPEDR_
Q64430	1438	S	ATP7A	_SPSEISVHVGIDDTSRNS[Phospho (STY)]PR_
A2A8U2	612	S	TMEM201	_KEDESS[Phospho (STY)]QS[Phospho (STY)]S[Phospho (STY)]T[Phospho (STY)]C[Carbamidomethyl (C)]VVDTTTK_
A2A8U2	613	S	TMEM201	_KEDESS[Phospho (STY)]QS[Phospho (STY)]S[Phospho (STY)]T[Phospho (STY)]C[Carbamidomethyl (C)]VVDTTTK_
Q8K2L8	314	S	TRAPPC12	_SPDSTS[Phospho (STY)]PSYSTR_
P16092	797	S	FGFR1	_SSTC[Carbamidomethyl (C)]SSGEDSVFS[Phospho (STY)]HEPLPEEPC[Carbamidomethyl (C)]LPR_
Q5DTU0	346	S	AFAP1L2	_KKSTS[Phospho (STY)]LEPPER_
Q8VCW4	547	S	UNC93B1	_YLEEDNS[Phospho (STY)]DES[Phospho (STY)]DMEGEQGQGDC[Carbamidomethyl (C)]AEDEAPQAGPLGAEPAGPC[Carbamidomethyl (C)]R_
Q8BSK8	250	T	RPS6KB1	_ESIHDGTVT[Phospho (STY)]HTFC[Carbamidomethyl (C)]GTIEYMAPEILMR_
O54781	491	T	SRPK2	_TVSASST[Phospho (STY)]GDLPK_
Q9WV92	601	S	EPB41L3	_RLSTS[Phospho (STY)]PVR_
Q60695	629	S	RGL1	_SVS[Phospho (STY)]VTSITSTVLPPVYNQQNEDTC[Carbamidomethyl (C)]IIR_
Q04692	92	Y	SMARCAD1	_AIQEY[Phospho (STY)]IDLSSDTEDVSPNC[Carbamidomethyl (C)]SSTVQEK_
Q924H7	534	S	WAC	_SPSPGPNHTC[Carbamidomethyl (C)]SS[Phospho (STY)]NASTATVVPQNASAR_
Q8VD12	162	S	ZNF385A	_PVSMENGLGPAPGS[Phospho (STY)]PEKQPGSPS[Phospho (STY)]PPSVPESGQGVTK_
Q8R0F5	143	S	RBMX2	_GC[Carbamidomethyl (C)]GVKT[Phospho (STY)]PPS[Phospho (STY)]SPPEVS[Phospho (STY)]EDEDAK_
Q91WG5	73	S	PRKAG2	_KVDSPFSSGSPS[Phospho (STY)]R_
Q8CJF7	1930	S	AHCTF1	_EVS[Phospho (STY)]VS[Phospho (STY)]SVTEEPK_
Q3U214	156	S	MAST3	_S[Phospho (STY)]SESVVDEDGGR_
Q9WTQ5	1064	T	AKAP12	_VEEVEEDSEVLAT[Phospho (STY)]EK_
Q68FF6	392	Y	GIT1	_SQSELDDQHDY[Phospho (STY)]DSVASDEDTDQEPLPSAGATR_
O54988	439	T	SLK	_LENLPDTQDQQT[Phospho (STY)]VDVNSVSEENENNR_
Q4V9W2	143	S	SREK1IP1	_S[Phospho (STY)]SPYHSELTK_
Q8K3X4	316	S	IRF2BPL	_RPGS[Phospho (STY)]VSS[Phospho (STY)]TDQER_

Q9QYK7	25	S	RNF11	_AS[Phospho (STY)]FGEGTEPDQEPPPPYQEQVPVPIYHPTPSQTR_
A2AAE1	2726	S	BLTP1	_S[Phospho (STY)]SETFGPAGVR_
O08750	286	S	NFIL3	_SS[Phospho (STY)]DGEDEQQVPK_
Q99JP4	41	T	CDC26	_QKEDVEGVGT[Phospho (STY)]SDGEGAAGLSSDPK_
Q99MS7	180	S	EHBP1L1	_ATDDDMQSLASLMSVKPS[Phospho (STY)]DVGNLDDFAESDEEEANGPGAPEVR_
Q9Z1Q9	526	S	VARS1	_VQGSDS[Phospho (STY)]DEEVVVATTR_
Q8R5C8	421	S	ZMYND11	_KEEPEPETEAVSSS[Phospho (STY)]QEIPTMPQPIER_
Q7TN31	12	S	AGGF1	_[Acetyl (Protein N-term)]ASEAPSPPSPS[Phospho (STY)]PPPPASPEPELAQLR_
Q8VEC3	73	S	ADGRF1	_S[Phospho (STY)]PSPSLR_
Q5HZJ5	301	T	ENTREP3	_RDSQAT[Phospho (STY)]LFDPQLHDGSC[Carbamidomethyl (C)]VC[Carbamidomethyl (C)]ER_
Q9CQE6	166	S	ASF1A	_LEDAESS[Phospho (STY)]NPNLQSLLSTDALPSASK_
P23198	176	S	CBX3	_LTWHS[Phospho (STY)]C[Carbamidomethyl (C)]PEDEAQ_
Q8BTI8	1458	S	SRRM2	_ARS[Phospho (STY)]HS[Phospho (STY)]PSS[Phospho (STY)]PER_
Q8BVK9	175	S	SP110	_ATAQPIIEILDEQPS[Phospho (STY)]PSPR_
Q920Q8	338	S	IVNS1ABP	_SLS[Phospho (STY)]FEMQPDELLEKPMSPMQYAR_
Q5SVR0	1143	S	TBC1D9B	_ALQDSHVIVEGGS[Phospho (STY)]GEGQGSPSLLLSDDETK_
P42128	243	S	FOXK1	_SLVSPIPSPTGT[Phospho (STY)]ISVPNSC[Carbamidomethyl (C)]PAS[Phospho (STY)]PR_
P35123	656	Y	USP4	_SSY[Phospho (STY)]EGDEEEEMDHQEEGK_
O55134	1103	S	PCDH12	_TVGPGPELS[Phospho (STY)]PTGTR_
E9Q309	2809	S	CEP350	_SELEDEKEEISS[Phospho (STY)]PDMC[Carbamidomethyl (C)]PRPESPVFGASGQEELAK_
Q8BZ20	273	S	PARP12	_DSSGPVS[Phospho (STY)]PGTPSQEESEQIC[Carbamidomethyl (C)]LYHIR_
Q8C886	411	S	PLEKHN1	_MKPSDSS[Phospho (STY)]PSPR_
A2A690	427	T	TANC2	_ELPLTQAPSAHSSIT[Phospho (STY)]SGSC[Carbamidomethyl (C)]PGTPEMR_
Q8K031	232	S	STARD8	_S[Phospho (STY)]LSIESLC[Carbamidomethyl (C)]PDEGR_
Q0PHV7	129	S	DACT3	_SSGFYEDPSSTGGPDS[Phospho (STY)]PPSTFC[Carbamidomethyl (C)]GDSGFSGSGSYGR_
Q3U6K5	422	Y	SPATA6	_DSAY[Phospho (STY)]DSDPEYSSFQRPR_
Q62312	239	T	TGFBR2	_SDISSTC[Carbamidomethyl (C)]ANNINHNT[Phospho (STY)]ELLPIELDTLVGK_
B9EJ86	774	T	OSBPL8	_HRT[Phospho (STY)]PMVSVPK_
Q5SXY1	355	S	SPECC1	_SSKGS[Phospho (STY)]PTGSSPNNASELSLASLTEK_
Q91Z96	646	S	BMP2K	_LGASTPS[Phospho (STY)]DK_
Q9Z0X1	523	S	AIFM1	_SATEQS[Phospho (STY)]GTGIR_
Q8C180	121	S	FRS2	_S[Phospho (STY)]SHQTELEVPR_
Q6A068	429	T	CDC5L	_SGT[Phospho (STY)]TPKPVTNATPGR_
E9Q394	2354	T	AKAP13	_DMTEC[Carbamidomethyl (C)]ST[Phospho (STY)]PLPEDC[Carbamidomethyl (C)]SPTHSPR_
Q6P3Y5	570	S	ZNF280C	_ATTTS[Phospho (STY)]PQTVATTTGKPSASKPGTGTTK_
E9Q394	2531	S	AKAP13	_SASRPSS[Phospho (STY)]LIEQEK_
Q6DFV3	62	T	ARHGAP21	_T[Phospho (STY)]SQGFGFTLR_
Q3UE17	157	S	MEX3D	_S[Phospho (STY)]VNMTEC[Carbamidomethyl (C)]VPVPSSEHVAEIVGR_
Q5PSV9	623	T	MDC1	_EGAQT[Phospho (STY)]PTGR_

Q8BTI8	456	T	SRRM2	_EISSSPT[Phospho (STY)]SK_
Q8C310	832	S	ROBO4	_APS[Phospho (STY)]PPTTYGYISIPTC[Carbamidomethyl (C)]SGLADMGR_
P97465	353	T	DOK1	_LT[Phospho (STY)]DSKEDPIYDEPEGLAPAPPR_
Q3UH68	303	S	LIMCH1	_SWSTATS[Phospho (STY)]PLGGER_
Q9QWL7	34	S	KRT17	_LSGS[Phospho (STY)]LGAGSC[Carbamidomethyl (C)]R_
B1AYB6	300	S	MBD5	_TNIPLS[Phospho (STY)]PTLTTK_
Q6VNB8	979	S	WDFY3	_S[Phospho (STY)]SVITSLEGLGSDNVFSSHEDNHYR_
B1AQJ2	899	S	USP36	_KRS[Phospho (STY)]EGLSQEATPSQDLIQHSC[Carbamidomethyl (C)]SPVDHSEPEAR_
Q5PSV9	493	S	MDC1	_RPNDADEYMDMSS[Phospho (STY)]PGSHLVVNQASFAVVGK_
E9Q6J5	896	S	BOD1L	_TKS[Phospho (STY)]LLEDK_
E9Q784	1522	Y	ZC3H13	_LDDAHSLGSGAGEGY[Phospho (STY)]EPISDDELDEILAGDAEK_
Q7TSC1	1110	T	PRRC2A	_QRGSETGSET[Phospho (STY)]HESDLAPSDK_
P55258	185	S	RAB8A	_LEGNSPQGS[Phospho (STY)]SHGVK_
Q9QZM4	330	S	TNFRSF10B	_S[Phospho (STY)]ASINHLLDALEAVEER_
Q9R020	278	S	ZRANB2	_SYSSSS[Phospho (STY)]SSPER_
P97412	2157	S	LYST	_EEAFISS[Phospho (STY)]C[Carbamidomethyl (C)]ESAK_
E9Q4F7	1808	S	ANKRD11	_QQSVPAPSSFDS[Phospho (STY)]PVQHLLEEK_
Q6DFV5	1464	S	HELZ	_PQS[Phospho (STY)]PAAEAVGPEQPPPPGLPDGHSPLR_
P70324	379	S	TBX3	_DLC[Carbamidomethyl (C)]PSEAESDAEAES[Phospho (STY)]KEEHGPEAC[Carbamidomethyl (C)]DAAK_
O54827	487	S	ATP10A	_GS[Phospho (STY)]TGS[Phospho (STY)]HQSIWMTHK_
Q9Z1M0	443	S	P2RX7	_LS[Phospho (STY)]LSLHDSPLTPGQSEEIQLLHEEVAPK_
Q3V1L4	511	S	NT5C2	_NRTS[Phospho (STY)]VDFK_
P68254	64	S	YWHAQ	_VISS[Phospho (STY)]IEQK_
P06537	152	S	NR3C1	_STS[Phospho (STY)]RPENPK_
Q9ERU9	2503	Y	RANBP2	_NRPGY[Phospho (STY)]VSEEEEDDEDYEMAVK_
Q91Z67	994	S	SRGAP2	_TSPVVAPTSEPSS[Phospho (STY)]PLHTQLLK_
Q3TUF7	1367	T	YEATS2	_QALAVGYQT[Phospho (STY)]ASPNR_
Q9WVR4	452	S	FXR2	_TGGPAYGPSSDPS[Phospho (STY)]TASETESEKR_
E9Q4F7	1689	T	ANKRD11	_TEQSRPTGVPT[Phospho (STY)]PTSVVSC[Carbamidomethyl (C)]PSYEEVMHTPR_

Supplemental Table 3. Net unique peptide number of SGK3 interaction proteins identified by proximal labeling-MS

Accession	net_unique_peptides_number_Day 0	net_unique_peptides_number_Day3	Accession	net_unique_peptides_number_Day 0	net_unique_peptides_number_Day3
Q99PV0	34	69	E9Q9R9	0	1
Q9JHU4	52	67	F4I366	0	1
Q8BTM8	55	59	F4KHH8	0	1
E9Q555	70	55	F7BJB9	0	1
Q62261	33	50	G5E897	0	1
Q9ERU9	27	46	O08740	0	1
A5YKK6	0	45	O08811	0	1
Q8R1A4	36	43	O09172	0	1
Q6P4T2	20	36	O35074	0	1
Q9QXS1	57	36	O35083	0	1
Q8BUR4	13	26	O35134	0	1
O08810	21	26	O35344	0	1
P16546	13	24	O35450	0	1
Q80TP3	8	23	O35451	0	1
Q91ZX7	21	23	O35604	0	1
Q80WQ2	16	23	O35730	0	1
P46061	7	22	O55236	0	1
P55937	13	22	O70252	0	1
Q91ZW3	9	20	O70281	0	1
Q9DAW6	8	20	O70566	0	1
Q8BX02	13	20	O82233	0	1
Q62167	1	19	O88351	0	1
Q80TL7	11	19	O88967	0	1

P97390	14	19	O89114	0	1
B2RQC6	6	18	P00397	0	1
Q8CJG0	7	18	P00848	0	1
Q3UJB9	9	18	P03334	0	1
P26039	15	18	P04117	0	1
O08553	15	18	P05064	0	1
P23116	10	17	P09528	0	1
Q9Z1Q9	7	17	P15105	0	1
Q8BMK4	6	17	P15920	0	1
O35685	12	17	P18872	0	1
O88738	2	16	P19258	0	1
A2A5R2	3	16	P22315	0	1
Q6PDQ2	5	16	P23492	0	1
Q61595	4	16	P23780	0	1
Q9EP71	11	16	P23798	0	1
Q3TKT4	7	16	P24100	0	1
Q3TMW1	18	16	P26043	0	1
P58871	5	15	P28271	0	1
Q8C129	6	15	P28798	0	1
Q8BTZ4	8	15	P30285	0	1
Q9DCL9	13	15	P31748	0	1
P04264	1	14	P35689	0	1
Q8VDM4	5	14	P35922	0	1
Q3TLI0	3	14	P45376	0	1
O55131	6	14	P47199	0	1
P29341	10	14	P47809	0	1

Q3UIR3	13	14	P48755	0	1
Q8BKT8	12	14	P49452	0	1
Q8CGC7	10	13	P49586	0	1
Q8QZX2	9	13	P50397	0	1
P70302	9	13	P50518	0	1
Q8CAS9	14	13	P51175	0	1
Q8VHX6	12	13	P56960	0	1
Q8VDP4	6	12	P57680	0	1
Q9JKF1	11	12	P58059	0	1
Q8K1R7	6	12	P61226	0	1
P17426	5	12	P62774	0	1
Q80UG5	5	12	P62823	0	1
Q9D0I9	12	12	P62843	0	1
Q8BHL5	2	12	P63328	0	1
Q8JZQ9	11	12	P68037	0	1
A2RSJ4	10	12	P92974	0	1
O88492	6	12	P97350	0	1
E9PZJ8	8	11	P97363	0	1
Q8VDD5	6	11	P97465	0	1
P42932	5	11	P97470	0	1
Q61191	5	11	P9WQ23	0	1
Q8VD65	3	11	Q01279	0	1
Q9WUM4	6	11	Q01469	0	1
Q99KJ8	5	11	Q02248	0	1
Q99KP6	6	11	Q05816	0	1
Q80X90	17	11	Q05D44	0	1

Q68FL6	2	11	Q07231	0	1
P55072	9	11	Q07235	0	1
Q64331	8	11	Q08093	0	1
P46977	0	10	Q0VGB7	0	1
P70398	0	10	Q0WVF5	0	1
Q80YV3	0	10	Q11011	0	1
P20152	1	10	Q148V8	0	1
P57780	1	10	Q3EBQ3	0	1
Q9EPU4	2	10	Q3TB82	0	1
Q6P5F9	4	10	Q3TLH4	0	1
O54692	3	10	Q3TPX4	0	1
Q61037	3	10	Q3TYA6	0	1
P47758	2	10	Q3U2P1	0	1
Q8BML9	5	10	Q3UDP0	0	1
Q9EPU0	10	10	Q3UFS0	0	1
Q8BU30	10	10	Q3UMB5	0	1
B2RXC1	4	10	Q3UN02	0	1
Q9DBC3	5	10	Q3UPH7	0	1
Q7TMQ7	7	10	Q3UVG3	0	1
E9Q7G0	0	9	Q3UVL4	0	1
O55143	1	9	Q505K2	0	1
E9PVA8	1	9	Q52KR3	0	1
Q8BTS4	3	9	Q569Z6	0	1
Q6P5D8	4	9	Q5D862	0	1
Q9WUN2	3	9	Q5NCF2	0	1
Q8C570	3	9	Q5SUR0	0	1

Q91VH2	3	9	Q5SVD0	0	1
O54774	4	9	Q5XJY4	0	1
P97434	5	9	Q60759	0	1
P43686	5	9	Q60790	0	1
Q6PDG5	5	9	Q60870	0	1
A2AWA9	3	9	Q60949	0	1
Q61881	9	9	Q61062	0	1
P83093	7	9	Q61097	0	1
Q5ND34	4	9	Q61136	0	1
Q80XQ2	5	9	Q61210	0	1
Q07113	4	9	Q61749	0	1
P47757	8	9	Q61771	0	1
Q9D786	12	9	Q62137	0	1
Q8C1B7	6	9	Q62203	0	1
Q9CQJ2	7	9	Q62388	0	1
P47754	6	9	Q683I9	0	1
Q9Z1T6	11	9	Q69ZR9	0	1
Q8CGN5	0	8	Q69ZS7	0	1
Q9JKR6	0	8	Q6EDY6	0	1
A2AN08	0	8	Q6GYP7	0	1
Q9JI10	1	8	Q6IME9	0	1
Q99P88	1	8	Q6KAR6	0	1
Q8R4U7	1	8	Q6NSU3	0	1
P28660	1	8	Q6NWV3	0	1
Q9CVB6	1	8	Q6PA06	0	1
Q6PGL7	2	8	Q6PAR5	0	1

Q8C8U0	3	8	Q6PB44	0	1
Q9R190	1	8	Q6PD26	0	1
Q80U93	2	8	Q6PDM2	0	1
P42208	3	8	Q6TEK5	0	1
Q5SNZ0	3	8	Q6VN19	0	1
O08788	4	8	Q6ZPZ3	0	1
Q6A0A9	3	8	Q6ZQ29	0	1
Q6PE01	3	8	Q6ZQI3	0	1
Q7TMB8	6	8	Q6ZWR6	0	1
Q69ZN7	8	8	Q6ZWX6	0	1
Q8BH15	2	8	Q7KZI7	0	1
Q61235	3	8	Q7M6Z4	0	1
Q99PL5	6	8	Q7TN98	0	1
P62192	5	8	Q7TPD0	0	1
Q8BGZ4	4	8	Q7TQK1	0	1
Q8BJ71	6	8	Q80TE0	0	1
Q8BVU0	6	8	Q80U70	0	1
Q9JKY0	6	8	Q80UM3	0	1
Q61696	8	8	Q80UW5	0	1
Q6NZJ6	8	8	Q80X50	0	1
O35286	5	8	Q80XC2	0	1
Q99L00	8	8	Q80XR2	0	1
Q5XJY5	3	8	Q80Y98	0	1
Q62419	3	8	Q810D6	0	1
Q60710	12	8	Q811U3	0	1
Q8R1B4	7	8	Q8BFV2	0	1

Q99MN1	4	8	Q8BG15	0	1
Q6P9Q6	8	8	Q8BHK9	0	1
P47753	5	8	Q8BHS3	0	1
Q920Q4	8	8	Q8BIW1	0	1
P02340	7	8	Q8BJW6	0	1
O08837	6	8	Q8BKT7	0	1
Q58A65	11	8	Q8BMB0	0	1
P41216	0	7	Q8BMJ3	0	1
Q9CXF4	0	7	Q8BN21	0	1
Q9D0E1	0	7	Q8BQY8	0	1
Q8VE19	1	7	Q8BRG8	0	1
P26638	1	7	Q8BTV2	0	1
Q91VR5	1	7	Q8BU14	0	1
Q8CH18	1	7	Q8BU33	0	1
Q9Z0U1	1	7	Q8BVN7	0	1
O54833	1	7	Q8BWR2	0	1
Q91XU0	1	7	Q8BWU5	0	1
A2AGT5	2	7	Q8BYA0	0	1
Q8K4B0	1	7	Q8BYL4	0	1
Q9DCD2	1	7	Q8BZ98	0	1
Q8BH74	2	7	Q8C050	0	1
P62908	2	7	Q8C0K5	0	1
Q9JLI8	3	7	Q8C407	0	1
Q8CFI7	2	7	Q8C4B4	0	1
Q3U0M1	1	7	Q8C6G1	0	1
Q9D6Z1	3	7	Q8C6L5	0	1

P46460	3	7	Q8CB65	0	1
Q68FD5	7	7	Q8CCB4	0	1
B2RY56	5	7	Q8CG76	0	1
P62334	8	7	Q8CI32	0	1
Q99NB9	9	7	Q8CI33	0	1
Q9CZX0	2	7	Q8CI71	0	1
Q6A4J8	3	7	Q8CIM8	0	1
Q4PZA2	4	7	Q8JZM0	0	1
Q8CH09	5	7	Q8K004	0	1
Q9CQS9	5	7	Q8K012	0	1
P57776	5	7	Q8K019	0	1
Q9QZD9	4	7	Q8K0C1	0	1
Q921M3	4	7	Q8K124	0	1
P17427	3	7	Q8K301	0	1
Q8CGZ0	2	7	Q8K358	0	1
P54645	4	7	Q8K4P0	0	1
Q01405	5	7	Q8L7F7	0	1
Q569Z5	4	7	Q8LPS6	0	1
P40142	4	7	Q8R054	0	1
Q9DBT5	5	7	Q8R0Z5	0	1
P47856	7	7	Q8R1V4	0	1
Q9Z1T1	12	7	Q8R326	0	1
P84091	4	7	Q8R3S6	0	1
Q60848	5	7	Q8R4F0	0	1
Q8R307	5	7	Q8R5K4	0	1
Q9DCN2	6	7	Q8RWL6	0	1

Q7TPH6	0	6	Q8RXK8	0	1
Q9Z0R4	0	6	Q8VD00	0	1
Q8BP47	0	6	Q8VD04	0	1
P08775	0	6	Q8VHH5	0	1
Q9D0F6	0	6	Q8VI63	0	1
Q8K0V4	0	6	Q91V09	0	1
Q7TPV4	0	6	Q91WC3	0	1
O55106	1	6	Q91WG2	0	1
P35550	1	6	Q91XB7	0	1
Q922U2	1	6	Q91YD9	0	1
P17156	1	6	Q91YE7	0	1
Q8CHH9	1	6	Q91YI0	0	1
Q60737	2	6	Q91YL3	0	1
P59328	2	6	Q91YM2	0	1
Q9ERG0	2	6	Q91YP3	0	1
Q8CIN4	2	6	Q922Q8	0	1
P20664	2	6	Q925N2	0	1
Q91WF7	2	6	Q93ZB1	0	1
Q8CG47	4	6	Q96KN8	0	1
Q6NV83	3	6	Q96QA5	0	1
Q3TDN2	1	6	Q96T76	0	1
Q3TDD9	2	6	Q99717	0	1
Q7TT37	2	6	Q99J09	0	1
P97377	3	6	Q99JB0	0	1
Q8BWQ6	3	6	Q99KU0	0	1
Q9WVJ2	3	6	Q99L47	0	1

P62196	4	6	Q99LB0	0	1
Q8C2E7	4	6	Q99LB6	0	1
Q8QZY1	2	6	Q99LC9	0	1
Q99JR8	2	6	Q99LH1	0	1
Q80UJ7	3	6	Q99MZ7	0	1
Q9Z277	4	6	Q99PL7	0	1
O09012	4	6	Q9C9A2	0	1
Q6P9P6	4	6	Q9CPV4	0	1
Q8BGD9	4	6	Q9CPW4	0	1
Q8CG48	7	6	Q9CQ71	0	1
P31230	4	6	Q9CQ75	0	1
Q99LF4	4	6	Q9CQG6	0	1
Q5SF07	3	6	Q9CQH3	0	1
Q9EP53	3	6	Q9CQP2	0	1
Q9R0A0	3	6	Q9CQR2	0	1
P97351	5	6	Q9CQS8	0	1
P39447	7	6	Q9CQV5	0	1
P07356	7	6	Q9CR27	0	1
P80315	3	6	Q9CR47	0	1
Q91YH5	3	6	Q9CS00	0	1
Q99KW3	6	6	Q9CWU9	0	1
Q8BJY1	4	6	Q9CY50	0	1
Q9D8N0	4	6	Q9CZ57	0	1
Q6Y685	5	6	Q9CZX9	0	1
Q8BHX1	9	6	Q9D1H8	0	1
P62983	6	6	Q9D1M0	0	1

Q99K48	0	5	Q9D1N9	0	1
Q7M6Y3	0	5	Q9D289	0	1
Q60634	0	5	Q9D4H1	0	1
O60506	0	5	Q9D5T0	0	1
P83741	0	5	Q9D7N3	0	1
Q02257	0	5	Q9D7S7	0	1
O70251	0	5	Q9D968	0	1
P42567	0	5	Q9D9V3	0	1
Q3UYV9	0	5	Q9DB05	0	1
Q6P4S8	0	5	Q9DB73	0	1
Q8K0D5	0	5	Q9DCU6	0	1
P61965	0	5	Q9EQ80	0	1
P59016	0	5	Q9EQP2	0	1
Q64435	0	5	Q9EQW7	0	1
Q8VHR5	0	5	Q9ER00	0	1
P33174	1	5	Q9ESX5	0	1
Q80TM9	1	5	Q9FGE6	0	1
Q3U9G9	1	5	Q9FK93	0	1
E9Q5G3	1	5	Q9FLB0	0	1
Q6NS46	1	5	Q9JHE7	0	1
Q8VIJ6	1	5	Q9JIG7	0	1
P53995	1	5	Q9JJ00	0	1
P97496	1	5	Q9JM51	0	1
Q9WUA2	1	5	Q9LFG2	0	1
Q9Z321	1	5	Q9LPC4	0	1
P43247	2	5	Q9LS76	0	1

Q8BVY0	1	5	Q9LVP0	0	1
Q9DC48	1	5	Q9LYV3	0	1
P01027	2	5	Q9MAN1	0	1
P61161	2	5	Q9QXZ0	0	1
P70698	2	5	Q9QY76	0	1
Q8BVE3	2	5	Q9R0Q9	0	1
Q9UBH6	2	5	Q9R0U0	0	1
O70194	2	5	Q9R1P1	0	1
Q61768	2	5	Q9R233	0	1
Q9CZU3	2	5	Q9SYM2	0	1
P14148	3	5	Q9U903	0	1
Q920B9	2	5	Q9WUU8	0	1
Q64337	2	5	Q9WUV0	0	1
Q9ESZ8	2	5	Q9WVL0	0	1
Q9CXY6	4	5	Q9XEA1	0	1
Q9JIF7	3	5	Q9Z1E4	0	1
Q8JZX4	3	5	Q9Z1G4	0	1
Q8BGC4	3	5	Q9Z1N2	0	1
P46471	6	5	Q9Z1Q5	0	1
P80316	2	5	Q9Z1W8	0	1
Q99LE6	3	5	Q9Z2D8	0	1
Q9CQF3	3	5	Q9ZUT8	0	1
P57784	3	5	P62245	0	1
P97311	7	5	P62821	0	1
Q9QUI0	4	5	P00405	0	1
Q8VDD8	3	5	Q1W617	0	1

Q8BG32	6	5	Q8BHT6	0	1
Q3UMB9	7	5	Q8BMD8	0	1
Q00519	2	5	Q8VEH3	0	1
Q91YJ2	2	5	Q9D3B1	0	1
Q8CGY8	5	5	P23198	0	1
P49718	3	5	Q3TZZ7	0	1
P12970	6	5	Q64008	0	1
Q6PFD9	4	5	Q6ZWY3	0	1
Q61187	6	5	Q8R1L4	0	1
Q9WUM3	3	5	Q99JI6	0	1
O35075	4	5	Q9CXW4	0	1
P46935	11	5	P04406	0	1
Q9WVK4	6	5	P10605	0	1
Q64282	5	5	P36916	0	1
Q6PHZ2	5	5	P59997	0	1
Q60605	0	4	P63272	0	1
Q80SW1	0	4	Q61263	0	1
Q60715	0	4	Q78IK2	0	1
Q8BG51	0	4	Q8K212	0	1
A2A791	0	4	Q8K2X3	0	1
P19536	0	4	Q8R5J9	0	1
P63001	0	4	Q8VBZ3	0	1
Q61035	0	4	Q91YX5	0	1
Q8K2L8	0	4	Q99N87	0	1
P10852	0	4	Q9CQX2	0	1
O35551	0	4	Q9WU56	0	1

Q01768	0	4	A6H8H2	0	1
Q811U4	0	4	B9EJ80	0	1
Q922R1	0	4	F4HW79	0	1
P21810	0	4	F4JRJ6	0	1
Q61584	0	4	G5E8P0	0	1
P62046	0	4	O08585	0	1
Q64521	0	4	O08808	0	1
Q6P5E4	0	4	O15020	0	1
Q91W96	0	4	O22173	0	1
Q9JIH2	0	4	O35218	0	1
P26516	0	4	O48686	0	1
Q9CWX9	0	4	O55023	0	1
Q8K2Z4	0	4	O70422	0	1
Q3UFY8	0	4	O88477	0	1
Q3UPF5	0	4	P10078	0	1
Q60809	0	4	P11688	0	1
Q62448	0	4	P35235	0	1
Q8BMG7	0	4	P45481	0	1
Q8R2K4	0	4	P46425	0	1
Q4VA53	0	4	P46467	0	1
Q99JY9	0	4	P53569	0	1
G5E870	0	4	P58404	0	1
P22892	0	4	P62700	0	1
B1AZI6	0	4	P62876	0	1
Q9Z0W3	0	4	P69566	0	1
P49817	0	4	P70255	0	1

Q01320	0	4	P97760	0	1
P63038	1	4	Q05769	0	1
Q8BX17	1	4	Q15528	0	1
O08528	1	4	Q39218	0	1
O09106	1	4	Q3U1J4	0	1
Q8BGH2	1	4	Q3UDW8	0	1
Q8BTI8	1	4	Q4VAA7	0	1
Q9Z0H3	1	4	Q5E924	0	1
Q3TTY5	1	4	Q5SSK3	0	1
Q8BUK6	1	4	Q60996	0	1
P50516	1	4	Q61122	0	1
Q99LI8	1	4	Q61466	0	1
Q9CWM4	1	4	Q61545	0	1
P46735	1	4	Q62073	0	1
P59999	1	4	Q62311	0	1
Q6NV72	1	4	Q62441	0	1
Q9D0W5	1	4	Q63871	0	1
Q9JK92	1	4	Q6NZA9	0	1
P58252	3	4	Q6P3E7	0	1
P55096	1	4	Q6PDN3	0	1
Q00612	1	4	Q6PE84	0	1
Q9WVA3	1	4	Q6PR54	0	1
Q6ZQ88	1	4	Q6WKZ8	0	1
Q8BTY8	1	4	Q6ZQ58	0	1
Q9QZB7	1	4	Q78T54	0	1
Q91VW5	4	4	Q78XF5	0	1

P61979	1	4	Q7TQK4	0	1
Q9DC23	1	4	Q80W47	0	1
O54950	1	4	Q80X41	0	1
P59708	1	4	Q80YS6	0	1
Q28677	1	4	Q8BQZ4	0	1
Q3TIR1	1	4	Q8BSQ9	0	1
Q3UEB3	1	4	Q8BTW3	0	1
Q9JHI7	1	4	Q8BX09	0	1
P28740	2	4	Q8BXK8	0	1
Q61316	2	4	Q8BY71	0	1
P80318	3	4	Q8C052	0	1
Q8K363	2	4	Q8C0E2	0	1
P63154	2	4	Q8C9B9	0	1
P14869	2	4	Q8CBE3	0	1
Q8VCM8	2	4	Q8CBW3	0	1
Q9CQW9	2	4	Q8CD10	0	1
Q8C092	2	4	Q8CHK4	0	1
Q8K0C9	2	4	Q8CI11	0	1
Q6QI06	3	4	Q8CIG8	0	1
Q9CW03	4	4	Q8K2A8	0	1
P62495	3	4	Q8K3B1	0	1
P62267	3	4	Q8K3Z9	0	1
P24547	3	4	Q8R001	0	1
P27048	3	4	Q8R3C0	0	1
Q80Y56	3	4	Q8R4H9	0	1
Q9QWY8	4	4	Q8VE65	0	1

Q3TDQ1	2	4	Q8VEG6	0	1
Q6P9Z1	2	4	Q8VI75	0	1
Q6PGC1	2	4	Q91XL3	0	1
P19253	4	4	Q921D4	0	1
Q9WTI7	9	4	Q924K8	0	1
P06151	5	4	Q924Z4	0	1
Q810B6	3	4	Q96KR1	0	1
Q922B2	9	4	Q99K43	0	1
P13020	5	4	Q99K46	0	1
Q99K70	3	4	Q99NH2	0	1
O70433	3	4	Q9CAG5	0	1
O88597	3	4	Q9CQI9	0	1
P40336	3	4	Q9CT10	0	1
Q60902	3	4	Q9CWK3	0	1
Q6Y7W8	3	4	Q9CX13	0	1
Q9JHS3	4	4	Q9CZE3	0	1
Q9WV32	4	4	Q9D024	0	1
Q9D706	5	4	Q9D032	0	1
P62259	4	4	Q9D1H7	0	1
Q9EQH3	7	4	Q9D2V8	0	1
P54116	5	4	Q9DBJ1	0	1
Q6IFX2	0	3	Q9DBR3	0	1
P56480	0	3	Q9DCT6	0	1
P14733	0	3	Q9JIG8	0	1
Q99JB2	0	3	Q9JJK2	0	1
P50544	0	3	Q9JK91	0	1

Q8C2Q3	0	3	Q9JLM8	0	1
P54310	0	3	Q9LHN5	0	1
Q62351	0	3	Q9LZF6	0	1
A2AF47	0	3	Q9M8M3	0	1
P62814	0	3	Q9WV70	0	1
Q6PAM1	0	3	Q9Z207	0	1
Q8VDD9	0	3	Q05920	0	1
Q9CQV8	0	3	P53986	0	1
Q9QWL7	0	3	Q5U458	0	1
P80317	0	3	Q8BWT1	0	1
P62301	0	3	Q99JX7	0	1
P62830	0	3	Q99MR8	0	1
Q99MJ9	0	3	O08756	0	1
P54823	0	3	P11757	0	1
P13864	0	3	P27612	0	1
P14824	0	3	P52875	0	1
O88796	0	3	P11103	0	1
P28658	0	3	Q3U186	0	1
P35283	0	3	Q60855	0	1
P83510	0	3	Q61655	0	1
P97789	0	3	Q91V01	0	1
Q14643	0	3	Q9CQU3	0	1
Q68FF6	0	3	Q9CRC8	0	1
Q6QD59	0	3	Q9CXW2	0	1
Q8CI95	0	3	Q9DBE8	0	1
Q99ME2	0	3	A2ABV5	0	1

Q99PU8	0	3	A2BE28	0	1
Q9R1Q9	0	3	O88544	0	1
Q9Z1Q2	0	3	P14234	0	1
P13439	0	3	P27601	0	1
Q80WJ7	0	3	P62743	0	1
Q8BH04	0	3	Q04690	0	1
Q9ERA6	0	3	Q61414	0	1
O88895	0	3	Q6DFW0	0	1
P50247	0	3	Q80WT5	0	1
P62874	0	3	Q8C5N3	0	1
P70336	0	3	Q8R5C5	0	1
Q60864	0	3	Q8VCD5	0	1
Q8BKX1	0	3	Q921G6	0	1
Q8C6G8	0	3	Q922D4	0	1
Q91ZJ5	0	3	Q99NH0	0	1
Q9CQ56	0	3	Q9CWW7	0	1
Q9CRT8	0	3	Q9CWZ3	0	1
Q9D6M3	0	3	Q9CYL5	0	1
Q9D824	0	3	Q9DC28	0	1
Q9ES28	0	3	Q9EQC5	0	1
Q9JI44	0	3	Q9QXD8	0	1
Q9QY06	0	3	Q9R1P0	0	1
Q9Z1Z2	0	3	Q8K224	0	1
Q99LR1	0	3	Q922H2	0	1
Q9D0R2	0	3	Q9DB20	0	1
Q9Z2I9	0	3	P42669	0	1

O55126	0	3	P97379	0	1
Q8BRH0	0	3	Q99JX4	0	1
Q8R323	0	3	Q9EPE9	0	1
Q148V7	0	3	P24788	0	1
Q6PAL0	0	3	Q62376	0	1
Q8BKX6	0	3	Q64127	0	1
Q8CHI8	0	3	Q6KAQ7	0	1
Q8K389	0	3	Q6KCD5	0	1
Q9CZ62	0	3	Q8K2A7	0	1
Q8C854	0	3	Q9D753	0	1
P14685	0	3	Q9EQQ9	0	1
P32233	0	3	Q03265	0	1
Q9CSU0	0	3	Q61937	0	1
P63280	0	3	Q9R112	0	1
Q62018	0	3	Q62318	0	1
Q6NZC7	0	3	Q8BH64	0	1
Q91WQ5	0	3	Q78PY7	0	1
Q64514	0	3	Q8BG05	0	1
P70388	0	3	Q8K310	0	1
Q91WG8	0	3	P48678	2	1
Q9QXE7	0	3	P53994	1	1
Q8CJF7	1	3	Q9WTX6	1	1
Q8CEC0	1	3	Q2KN98	1	1
Q501J6	1	3	Q80U72	1	1
O35593	1	3	P62702	1	1
P35980	1	3	P70333	1	1

P70288	1	3	Q810S1	1	1
Q3TXS7	1	3	Q9QZQ8	1	1
P20029	3	3	P30999	1	1
P47963	1	3	Q91WS0	1	1
Q9Z1Z0	1	3	P61290	1	1
Q91YQ5	2	3	Q3TZX8	1	1
P60122	1	3	Q99PQ1	1	1
P42125	1	3	Q9CQN1	2	1
P28028	1	3	Q9CPN8	2	1
Q9JKC8	1	3	P27546	2	1
A2A8Z1	1	3	Q9Z110	3	1
E9Q3L2	1	3	Q9CPR4	2	1
Q8BRF7	1	3	Q9QXB9	2	1
P62827	1	3	P11440	4	1
P42227	1	3	P08238	6	1
Q9Z2I8	1	3	P50446	1	1
O35643	1	3	P63276	1	1
P51863	1	3	Q920A5	1	1
P61759	1	3	Q921F4	1	1
Q3TUF7	1	3	Q9D051	1	1
Q505D1	1	3	A2RSY6	1	1
Q99J45	1	3	O35071	1	1
Q9D0D4	1	3	O64644	1	1
Q9D0M5	1	3	P28301	1	1
Q9DAJ4	1	3	P42859	1	1
Q3MHN2	1	3	P50543	1	1

Q99JR1	1	3	P62962	1	1
Q9CY27	1	3	Q3UH66	1	1
P60335	1	3	Q56A07	1	1
Q9CWK8	1	3	Q6DID7	1	1
Q3TCN2	1	3	Q8BH65	1	1
Q61033	1	3	Q8BIJ7	1	1
Q8BFQ4	1	3	Q8BS39	1	1
Q9ERG2	1	3	Q8K2A1	1	1
O88685	2	3	Q8R0H9	1	1
P58854	2	3	Q8VE97	1	1
Q8CFQ3	2	3	Q99J78	1	1
Q9CU62	3	3	Q99J93	1	1
Q8R349	2	3	Q9D666	1	1
Q9Z2N8	2	3	Q9QYR6	1	1
Q91YR7	4	3	Q9SEI2	1	1
Q9Z2K1	2	3	Q9Z2W0	1	1
Q9CQW2	2	3	P0DOV1	1	1
Q9ES74	2	3	P14206	1	1
P09405	2	3	P47964	1	1
Q5KU39	2	3	P61205	1	1
Q69Z37	2	3	P61358	1	1
Q8BWY9	2	3	Q8BFZ9	1	1
P80314	2	3	Q99KQ4	1	1
Q91V61	2	3	O54946	1	1
Q8K245	2	3	P39054	1	1
Q9CQR6	2	3	P58742	1	1

Q9Z2Q6	2	3	Q01730	1	1
Q8BK63	2	3	Q3THS6	1	1
Q9JLV1	2	3	Q9CXV1	1	1
Q99J95	2	3	Q9QX47	1	1
Q9CSN1	2	3	Q9Z1F9	1	1
Q9QXK7	2	3	F4IVL6	1	1
Q9Z204	2	3	O35796	1	1
Q9D8W5	4	3	O70404	1	1
Q8BJS4	7	3	O88455	1	1
Q9QZE5	7	3	P03081	1	1
P81795	3	3	P14069	1	1
P37913	3	3	P35979	1	1
Q9CPU0	3	3	P41731	1	1
Q8BFT2	6	3	P47713	1	1
Q9Z1D1	3	3	P62307	1	1
P62315	3	3	P70218	1	1
Q60932	3	3	P70347	1	1
Q9EQ06	3	3	Q09XV5	1	1
Q9JLQ2	3	3	Q14444	1	1
Q8CDM1	2	3	Q8C863	1	1
Q8BMG8	2	3	Q8CB77	1	1
Q922D8	2	3	Q8K3P5	1	1
O88653	2	3	Q8RXS6	1	1
P14873	2	3	Q93Y08	1	1
P97855	2	3	Q96AX1	1	1
Q7TSZ8	2	3	Q9D4F8	1	1

Q9R1C7	2	3	Q9D662	1	1
Q60953	5	3	Q9D868	1	1
Q8CIE6	14	3	Q9D8S3	1	1
Q9WTK5	3	3	Q9DAW9	1	1
P61164	5	3	Q9ESU6	1	1
Q0P5W1	5	3	Q9WV80	1	1
Q8R010	4	3	Q9Z148	1	1
Q80TH2	7	3	P35278	1	1
Q8R081	5	3	P62858	1	1
Q60716	3	3	Q8K2C9	1	1
Q922U1	3	3	Q9CPQ8	1	1
O54941	3	3	Q9CQE8	1	1
P70257	3	3	Q9DCH4	1	1
Q8CID0	3	3	G5E829	1	1
Q8K4I3	3	3	O88545	1	1
Q923T9	3	3	Q8BHY2	1	1
Q9D0R8	3	3	Q8BKC5	1	1
Q61081	4	3	Q9D7B7	1	1
Q8BMB3	4	3	Q9QY36	1	1
Q61171	5	3	Q9QYB5	1	1
Q8BLK9	5	3	O88983	1	1
Q5SYD0	5	3	P43406	1	1
B1AVY7	12	3	P62305	1	1
Q9Z1N5	0	2	Q60631	1	1
E9Q557	0	2	Q8BFW7	1	1
P63325	0	2	Q99LD4	1	1

P70404	0	2	Q99M28	1	1
Q60931	0	2	Q9JJ28	1	1
Q3TCH7	0	2	Q8BKE6	3	1
P05213	0	2	P25206	5	1
P17809	0	2	Q3TEA8	5	1
Q9Z2A7	0	2	P14131	2	1
P10639	0	2	P84099	2	1
P26369	0	2	Q64364	2	1
P28867	0	2	Q9DBR1	2	1
Q8R1Q8	0	2	O35245	2	1
Q9JKX6	0	2	P26696	2	1
Q9QYA2	0	2	Q3UTZ3	2	1
Q9R1J0	0	2	Q60838	2	1
O09005	0	2	Q9Z1X4	4	1
P07742	0	2	Q60854	3	1
P48758	0	2	Q08943	3	1
P51912	0	2	Q6A065	2	1
Q8K2T1	0	2	Q9R0P5	2	1
Q8R0S2	0	2	P08556	2	1
A2AT37	0	2	P63323	2	1
A2RSY1	0	2	Q60749	2	1
B2RXR6	0	2	Q64737	2	1
O00178	0	2	P62137	2	1
O08759	0	2	P61750	2	1
O35864	0	2	Q91VD9	2	1
O88447	0	2	Q921T2	2	1

P01031	0	2	Q9ET30	2	1
P03387	0	2	Q3TJZ6	2	1
P24270	0	2	Q61029	2	1
P46100	0	2	P62317	2	1
P46737	0	2	P63166	2	1
P59110	0	2	Q3U0V1	2	1
P62869	0	2	Q80U35	2	1
P70670	0	2	Q921W4	2	1
P84096	0	2	Q9QXN3	2	1
Q3TCJ1	0	2	P42225	7	1
Q3UM18	0	2	Q9DBG3	4	1
Q497V5	0	2	P17751	3	1
Q5PSV9	0	2	P47911	3	1
Q61586	0	2	Q6ZWV3	6	1
Q62417	0	2	Q9CY64	4	1
Q6P9R4	0	2	Q9Z2X1	4	1
Q6ZPE2	0	2	Q60973	5	1
Q86UU1	0	2	O08547	3	1
Q8BIW9	0	2	O70152	4	1
Q8BM85	0	2	P0DOV2	8	1
Q8BX70	0	2	Q61699	5	1
Q8C0M0	0	2	Q9JJI8	4	1
Q8CBY8	0	2	P09103	1	0
Q8CFJ9	0	2	Q9CQQ7	1	0
Q8K296	0	2	Q91YE6	1	0
Q91YU8	0	2	Q9R0X4	1	0

Q99M96	0	2	Q7TQH0	1	0
Q9CPU4	0	2	Q69ZS0	1	0
Q9CQM2	0	2	P60843	1	0
Q9CWH6	0	2	Q99KI3	1	0
Q9CWS4	0	2	P63073	1	0
Q9CZH3	0	2	Q8BFY9	1	0
Q9ER73	0	2	Q6DFW4	2	0
Q9JLV5	0	2	Q61753	1	0
Q9WVC3	0	2	Q99L04	1	0
Q9Z315	0	2	Q9D819	1	0
O55029	0	2	Q9DB77	1	0
Q61686	0	2	E9QAT4	1	0
Q9D0F3	0	2	Q9JIH7	1	0
P19324	0	2	Q3U7R1	2	0
P61620	0	2	O70133	4	0
Q9DC16	0	2	Q8VDN2	2	0
Q9DCX2	0	2	P35601	3	0
Q80XL6	0	2	P35700	3	0
Q91WQ3	0	2	Q6ZQ08	20	0
A2A6Q5	0	2	P46978	6	0
O54931	0	2	Q9WU78	4	0
O70305	0	2	Q922F4	2	0
O70572	0	2	Q8BK64	2	0
P26450	0	2	P62751	3	0
P33215	0	2	Q8BH59	2	0
P61963	0	2	Q6P2B1	2	0

Q06330	0	2	Q921F2	3	0
Q5XG73	0	2	Q922R8	3	0
Q60967	0	2	Q8VEM8	3	0
Q61216	0	2	Q9CZD3	3	0
Q64378	0	2	P15361	3	0
Q64674	0	2	P67778	3	0
Q6PDI5	0	2	P35279	3	0
Q7TMY8	0	2	P49717	5	0
Q80XK6	0	2	Q64511	5	0
Q8BFR4	0	2	Q9CR68	2	0
Q8BGP6	0	2	P35585	2	0
Q8BK75	0	2	O35129	2	0
Q8BVL3	0	2	P62242	2	0
Q8BZH4	0	2	P68040	2	0
Q8K0H5	0	2	O08917	2	0
Q8K3W0	0	2	P62141	2	0
Q8R2T8	0	2	Q9EQU5	2	0
Q8R4C2	0	2	Q9ERE3	4	0
Q91Z38	0	2	Q9CR62	5	0
Q922Q1	0	2	P26443	3	0
Q924C1	0	2	Q9DBG6	3	0
Q99MR6	0	2	Q9QZ85	3	0
Q9CQT2	0	2	Q3UPL0	4	0
Q9D0I8	0	2	P62717	4	0
Q9JM13	0	2	Q8QZT1	4	0
Q9WTQ8	0	2	P63017	22	0

Q9WUD1	0	2	Q8CGK3	1	0
Q9Z1R2	0	2	P68368	2	0
Q9Z2D0	0	2	Q9R0E1	1	0
Q9CS42	0	2	E9Q4Z2	3	0
Q3THK7	0	2	Q9CZU6	1	0
Q9D1R9	0	2	P97820	1	0
P02535	0	2	P99024	1	0
P46638	0	2	Q99KI0	1	0
P80313	0	2	Q8BXQ2	1	0
Q78IK4	0	2	Q9CXT8	2	0
Q9D379	0	2	Q61548	2	0
Q9DC69	0	2	Q8VCW8	3	0
E9QAM5	0	2	P62737	1	0
O08529	0	2	P36371	3	0
O35609	0	2	Q99020	1	0
P33609	0	2	Q04857	2	0
P70677	0	2	Q07797	4	0
Q03958	0	2	Q9CX34	1	0
Q0GNC1	0	2	P61027	1	0
Q61103	0	2	B1ARD6	1	0
Q62193	0	2	P00374	1	0
Q6A026	0	2	P21550	1	0
Q6NVF9	0	2	Q80YU0	1	0
Q6P1F6	0	2	Q8BTX9	1	0
Q6ZQE4	0	2	Q8VCM7	1	0
Q6ZQH8	0	2	Q91WD5	1	0

Q7TSY8	0	2	Q9DC51	1	0
Q8BUV3	0	2	P51807	2	0
Q8C079	0	2	P55258	2	0
Q91WG4	0	2	Q63617	3	0
Q921N8	0	2	P11152	4	0
Q922J9	0	2	P54071	1	0
Q923J1	0	2	P61021	1	0
Q9D287	0	2	Q61550	1	0
Q9QYF1	0	2	Q61990	1	0
Q9R1T4	0	2	P39748	1	0
O08807	0	2	Q8BJM5	1	0
O55028	0	2	Q91YR1	2	0
P63094	0	2	Q99JW4	2	0
Q8JZU2	0	2	Q60766	3	0
O35343	0	2	P61211	1	0
P35821	0	2	Q64475	1	0
P52479	0	2	Q9D1G1	1	0
P61202	0	2	P11928	2	0
Q3U319	0	2	P41105	2	0
Q5F2E7	0	2	Q3TUH1	2	0
Q8BT07	0	2	Q9CX86	2	0
Q8CHY6	0	2	P62071	2	0
Q8VCX5	0	2	Q5FWK3	2	0
Q91V92	0	2	P10107	5	0
Q922P9	0	2	P61222	3	0
Q923D4	0	2	Q61553	1	0

Q9D3S3	0	2	Q2QL90	3	0
Q9QUM9	0	2	O55142	1	0
Q9WV55	0	2	P12815	1	0
P54276	0	2	P47915	1	0
Q99ME9	0	2	P68181	1	0
Q5XJE5	0	2	Q07076	1	0
Q6ZQ93	0	2	P42128	1	0
Q7TT50	0	2	P59326	1	0
Q80U95	0	2	Q3UFY0	1	0
Q8BQZ5	0	2	Q8RX72	1	0
Q8K2T8	0	2	Q9CQT1	1	0
P17182	0	2	Q9EQ61	1	0
E9PYH6	0	2	G5E8K5	1	0
Q9JIK5	0	2	O04656	1	0
Q8VEK3	0	2	O08734	1	0
Q04750	0	2	O23147	1	0
Q99P72	0	2	O35638	1	0
Q9WUK4	1	2	O54798	1	0
A2BH40	1	2	O70435	1	0
Q63850	1	2	P01948	1	0
Q00PI9	2	2	P02788	1	0
P62918	1	2	P08071	1	0
Q99J62	1	2	P08226	1	0
Q9ERK4	1	2	P11404	1	0
Q8VBV3	1	2	P29387	1	0
Q8C0C7	2	2	P32067	1	0

P21278	2	2	P35565	1	0
P19096	3	2	P48036	1	0
Q8BP67	1	2	P48455	1	0
Q8R0X7	1	2	P50295	1	0
Q8R0G9	1	2	P60764	1	0
Q9DB25	1	2	P84104	1	0
Q9QXX4	1	2	P98083	1	0
Q9Z103	1	2	Q03141	1	0
P62309	1	2	Q08857	1	0
Q62188	1	2	Q14141	1	0
Q8BTU1	1	2	Q14DK5	1	0
Q8R332	1	2	Q27968	1	0
Q8VEE4	1	2	Q3UL36	1	0
Q91VM3	1	2	Q4FZC9	1	0
Q9CQI7	1	2	Q5SQP1	1	0
Q9CWN7	1	2	Q61390	1	0
Q9D1L9	1	2	Q63844	1	0
Q9D8Z1	1	2	Q6ZPF4	1	0
Q9JJG0	1	2	Q6ZQ06	1	0
O70318	1	2	Q7TNV0	1	0
Q8R1K1	1	2	Q80TQ2	1	0
P68373	1	2	Q80U44	1	0
Q8K3G9	1	2	Q80Y86	1	0
Q9D0N7	1	2	Q84K16	1	0
Q9Z160	1	2	Q84WL9	1	0
P62900	1	2	Q8BHJ5	1	0

Q99L45	1	2	Q8BMQ2	1	0
Q9ER88	1	2	Q8BWQ1	1	0
Q9R1T2	1	2	Q8BYC6	1	0
A2AAY5	1	2	Q8BZ20	1	0
E9Q8I9	1	2	Q8C159	1	0
O55013	1	2	Q8C6Z1	1	0
P58801	1	2	Q8CFE4	1	0
P61982	1	2	Q8K3R3	1	0
P63087	1	2	Q8L7S0	1	0
P83940	1	2	Q8LAH8	1	0
Q3URQ0	1	2	Q8R151	1	0
Q5HZI9	1	2	Q8R5F7	1	0
Q8BH48	1	2	Q8RY66	1	0
Q8BKH7	1	2	Q8VCR7	1	0
Q8BZ36	1	2	Q8VY00	1	0
Q99LM9	1	2	Q91VK1	1	0
Q9CYN9	1	2	Q91WR3	1	0
Q9D554	1	2	Q91YN0	1	0
Q64133	1	2	Q91Z31	1	0
Q9D883	1	2	Q91ZU6	1	0
O88384	1	2	Q944S1	1	0
P63168	1	2	Q9C648	1	0
P63321	1	2	Q9D0B0	1	0
Q3UHJ0	1	2	Q9DCF9	1	0
Q3UMG5	1	2	Q9ERI2	1	0
Q64433	1	2	Q9JMK0	1	0

Q6PF93	1	2	Q9LHG9	1	0
Q7TSI3	1	2	Q9LRV8	1	0
Q8CGB3	1	2	Q9LSJ6	1	0
Q8R050	1	2	Q9QUJ7	1	0
Q91ZE0	1	2	Q9WUQ2	1	0
Q9CYZ2	1	2	Q9Z2F2	1	0
Q9D0M0	1	2	Q9ZRF9	1	0
Q9JJY4	1	2	O88569	2	0
P62754	2	2	Q91YM4	2	0
Q9QZD8	2	2	A0JNP2	2	0
Q6ZQ38	4	2	O08573	2	0
Q9WV92	3	2	O75175	2	0
P63037	2	2	P23249	2	0
Q3UW53	2	2	P68510	2	0
Q922V4	2	2	P80195	2	0
Q9DBS1	2	2	Q9D2G9	2	0
Q9D8E6	5	2	Q9DBP0	2	0
Q9JIF0	2	2	Q9UNQ0	3	0
Q8K1M6	2	2	P07900	7	0
P08752	2	2	P51881	1	0
Q60520	2	2	P60712	1	0
Q8VDJ3	3	2	P62264	1	0
P17918	3	2	P62855	1	0
P27773	3	2	P62911	1	0
Q6ZWN5	3	2	Q9CR67	1	0
P18652	2	2	D3Z7P3	1	0

Q60960	2	2	O35658	1	0
P31938	2	2	O88696	1	0
Q9D5V5	2	2	P39053	1	0
P70227	2	2	P63082	1	0
F8VPU2	2	2	Q4KWH5	1	0
O89079	2	2	Q60632	1	0
Q9CY58	2	2	Q8BIJ6	1	0
Q9WVL2	2	2	Q8R404	1	0
P62320	2	2	Q8VDP6	1	0
Q60930	2	2	Q96292	1	0
Q9QYC0	2	2	Q99LC8	1	0
O35350	2	2	Q9CR60	1	0
Q64012	2	2	Q9D8N2	1	0
Q80X95	2	2	Q9ES97	1	0
Q9CZB0	2	2	Q9JK42	1	0
Q69Z38	2	2	Q9JLR9	1	0
Q6PEB6	2	2	Q9WVR4	1	0
Q8C5L3	2	2	B2RY04	1	0
Q921G8	2	2	G3X9K3	1	0
Q60598	4	2	O23628	1	0
Q8VEH5	3	2	O23657	1	0
Q9CSH3	3	2	O70126	1	0
Q9CXW3	3	2	O70439	1	0
P62280	3	2	P15331	1	0
Q91YN9	3	2	P63208	1	0
P60228	5	2	Q3ULW8	1	0

P70168	5	2	Q6A009	1	0
P11983	6	2	Q6A0D4	1	0
Q3U0J8	8	2	Q6Q899	1	0
Q9DCA5	3	2	Q6ZPU9	1	0
Q8R2U0	3	2	Q7TPV2	1	0
Q99KN9	3	2	Q7TSH2	1	0
P40124	5	2	Q8BGQ1	1	0
P10126	6	2	Q8BPU7	1	0
Q791V5	5	2	Q8CF89	1	0
P25444	4	2	Q8CI59	1	0
P12382	5	2	Q8R570	1	0
Q9JIW9	0	1	Q920E5	1	0
Q8VH51	0	1	Q93ZR1	1	0
B9DHQ0	0	1	Q99MU3	1	0
P10833	0	1	Q9C5A9	1	0
P10922	0	1	Q9C5J3	1	0
P62331	0	1	Q9CRA8	1	0
P70699	0	1	Q9EPC1	1	0
Q02780	0	1	Q9WV60	1	0
Q69ZK0	0	1	Q9Z2X8	1	0
Q6ZWQ7	0	1	P17225	2	0
Q920D2	0	1	P53690	2	0
Q9D8M4	0	1	P08207	2	0
Q9R0P6	0	1	P67871	2	0
P18760	0	1	Q03350	2	0
P60867	0	1	Q6ZPR5	2	0

P62852	0	1	E9Q9A9	2	0
Q3THW5	0	1	Q9JHH9	2	0
Q8BGZ7	0	1	O88844	3	0
Q921X9	0	1	O54724	1	0
F4HPN2	0	1	O70503	1	0
P32261	0	1	P14115	1	0
P62270	0	1	P43274	1	0
Q62186	0	1	P62889	1	0
Q64339	0	1	Q64310	1	0
Q7TQI3	0	1	Q8C3X8	1	0
Q8BFZ3	0	1	Q99LC3	1	0
Q99K01	0	1	Q9CQD1	1	0
Q9CQR4	0	1	O70579	1	0
Q9D6R2	0	1	P52332	1	0
Q9T043	0	1	P70202	1	0
P31324	0	1	Q8VDW0	1	0
P37889	0	1	Q9CZ13	1	0
P50136	0	1	Q9WV02	1	0
P70372	0	1	O08709	1	0
Q61398	0	1	Q62348	1	0
Q8C1E7	0	1	Q80UU9	1	0
Q8K3K7	0	1	Q80W49	1	0
Q8R2Q4	0	1	Q8CDG3	1	0
Q921J2	0	1	Q8K4Z5	1	0
Q99J27	0	1	Q9QZQ1	1	0
Q9CYH2	0	1	P61255	2	0

Q9DCT5	0	1	Q03963	2	0
Q9EQ20	0	1	Q60875	2	0
A1L4W5	0	1	O55222	2	0
A2A935	0	1	P32921	2	0
A2AVZ9	0	1	P84244	2	0
A8MRP2	0	1	Q3THE2	2	0
C0LGG7	0	1	P01899	3	0
C0SUT9	0	1	Q7TPR4	3	0
E9PVB5	0	1	Q9D023	3	0
E9Q414	0	1	P51150	4	0
E9Q793	0	1	Q2EMV9	6	0

Supplemental Table 4. Intensities of BRG1 phosphorylation sites identified in *in vitro* kinase assay with or without SGK3 by MS.

Position	Modified Peptide Sequence	Best -10logP	Best AScore	Best Ion Intensity (%)	Best AScore -kinase	Best Ion Intensity (%) -kinase	Best AScore +kinase	Best Ion Intensity (%) +kinase	-kinase modified	-kinase unmodified	+kinase modified	+kinase unmodified
T428	QEVVVCMRRDtALETALNAKA	30.01	14.18	7	N/A	N/A	14.18	7	0	1290000000	2410000	1450000000
S613	GEPLDETSQMsDLPVKVIHVE	59.99	12.15	7	12.15	6	5.76	7	32200000	773000000	7460000	478000000
S695	EEKKKIPDPDsDDVSEVDARH	52.6	42.88	5	42.88	5	N/A	N/A	28400000	910000000	0	374000000
S699	KIPDPDSDDVsEVDARHIIEN	51.57	1000	13	16.5	13	1000	11	12400000	993000000	39100000	483000000
S1417	KKSSRKRKRDsDAGSSTPTTS	45.23	49.78	17	N/A	N/A	49.78	17	0	532000000	4600000	580000000

Supplemental Table 5. Antibodies used in the study.

Antibodies	Supplier	Identifier
Rabbit monoclonal SGK3	Cell Signaling Tech	Cat#8573, RRID: AB_10949896
Rabbit monoclonal BRG1	Cell Signaling Tech	Cat#49360, RRID: AB_2728743
Rabbit monoclonal RXX-p-S/T	Cell Signaling Tech	Cat#9614, RRID: AB_331810
Rabbit monoclonal p-NDRG1 S330	Cell Signaling Tech	Cat#11899, RRID: AB_2797761
Rabbit monoclonal p-NDRG1 T346	Cell Signaling Tech	Cat#5482, RRID: AB_10693451
Rabbit monoclonal NDRG1	Cell Signaling Tech	Cat#9485, RRID: AB_2721143
Rabbit monoclonal SMARCC2	Cell Signaling Tech	Cat#12760, RRID: AB_2798017
Rabbit monoclonal PPARγ	Cell Signaling Tech	Cat#2435, RRID: AB_2166051
Rabbit monoclonal C/EBPα	Cell Signaling Tech	Cat#8178, RRID: AB_11178517
Rabbit monoclonal Adiponectin	Cell Signaling Tech	Cat#2789, RRID: AB_2221630
Rabbit polyclonal FABP4	Cell Signaling Tech	Cat#2120, RRID: AB_2102466
Rabbit monoclonal ACC1	Cell Signaling Tech	Cat#3676, RRID: AB_2219397
Rabbit polyclonal HSL	Cell Signaling Tech	Cat#4107, RRID: AB_2296900
Mouse monoclonal Ub	Cell Signaling Tech	Cat#3936, RRID: AB_331292
Rabbit monoclonal SGK1	Cell Signaling Tech	Cat#12103, RRID: AB_2687476
Rabbit monoclonal p-AKT T308	Cell Signaling Tech	Cat#13038, RRID: AB_2629447
Rabbit monoclonal AKT	Cell Signaling Tech	Cat#4691, RRID: AB_915783
Mouse monoclonal Lamin A/C	Cell Signaling Tech	Cat#4777, RRID: AB_10545756
Rabbit polyclonal GR	ProteinTech	Cat#24050-1-AP, RRID: AB_2813890
Rabbit monoclonal FLAG	ProteinTech	Cat#80010-1-RR, RRID: AB_2882940
Mouse monoclonal FLAG	ProteinTech	Cat#66008-4-Ig, RRID: AB_2918475
Mouse monoclonal HSP90	ProteinTech	Cat#60318-1-Ig, RRID: AB_2881429
Mouse monoclonal β-Actin	ProteinTech	Cat#66009-1-Ig, RRID: AB_2687938
Rabbit polyclonal H3	ProteinTech	Cat#17168-1-AP, RRID: AB_2716755

Mouse monoclonal GST	ProteinTech	Cat#66001-2-Ig, RRID: AB_2881488
Rabbit monoclonal SMARCB1	Abclonal	Cat#A23466
Rabbit polyclonal Pan Phospho-Serine/Threonine	Abclonal	Cat#AP0893, RRID: AB_2770782
Rabbit polyclonal catalase	Abclonal	Cat#A11780, RRID: AB_2758751
Rabbit polyclonal MR	Abclonal	Cat#A3308, RRID: AB_2765039
Mouse monoclonal Pan Phospho-Serine	BD	Cat#612546, RRID: AB_399841
Mouse monoclonal Importin β1	abcam	Cat#AB2811, RRID: AB_2133989
Rabbit polyclonal ACTL6A	ProteinTech	Cat#10341-1-AP, RRID: AB_2289267
Rabbit polyclonal DPF1	ProteinTech	Cat#21769-1-AP, RRID: AB_3085672
Rabbit monoclonal ARID1A	Abclonal	Cat#A19570
Recombinant Rabbit monoclonal α-SMA	ProteinTech	Cat#80008-1-RR, RRID: AB_2882938
Rabbit polyclonal SGK3	ProteinTech	Cat#12699-1-AP, RRID: AB_2188548
HRP-conjugated streptavidin	Proteintech	Cat#SA00001-0
HRP-conjugated Goat Anti-Mouse IgG (H+L)	Jackson ImmunoResearch	Cat#115-005-003, RRID: AB_2338447
HRP-conjugated Goat Anti-Rabbit IgG (H+L)	Jackson ImmunoResearch	Cat#111-005-003, RRID: AB_2337913
HRP-conjugated AffiniPure Mouse Anti-Rabbit IgG Light Chain	Abclonal	Cat#AS061, RRID: AB_2864055
HRP-conjugated AffiniPure Goat Anti-Mouse IgG Light Chain	Abclonal	Cat#AS062, RRID: AB_2864056
Multi-rAb Polymer HRP-Goat Anti-Rabbit Recombinant Secondary Antibody (H+L)	ProteinTech	Cat#RGAR011, RRID: AB_3094534
CoraLite488-conjugated Goat Anti-Mouse IgG(H+L)	Proteintech	Cat#SA00013-1, RRID: AB_2810983
CoraLite594-conjugated Goat Anti-Rabbit IgG(H+L)	Proteintech	Cat#SA00013-4, RRID: AB_2810984
Mouse IgG	Proteintech	Cat#B900620, RRID: AB_2883054

Rabbit IgG	Proteintech	Cat#B900610, RRID: AB_3674206

Supplemental Table 6. Reagents used in the study.

Reagents	Supplier	Identifier
PROTAC SGK3 degrader-1	selleck	Cat#S9672
PROTAC SGK3 degrader-1	MCE	Cat#HY-125878
Corn oil	Aladdin	Cat#C116023
Tamoxifen	Sigma-Aldrich	Cat#T5648
doxycycline hyclate	Beyotime	Cat#ST039A
Water soluble dexamethasone	pSaitong	Cat#D80005
HFD	Research diet	Cat#12492
IBMX	Sigma	Cat#I5879
Rosiglitazone	Sigma	Cat#R2408
Dexamethasone	Sigma	Cat#D4902
Insulin	Beyotime	Cat#P3376
DMEM	Gibco	Cat#8117254
DMEM/F12	Gibco	Cat#11330032
Penicillin/Streptomycin	Beyotime	Cat# ST488
primocin	invivogen	Cat#ant-pm-1
FBS	Gibco	Cat#10270106
Dextran coated charcoal	Sigma-Aldrich	Cat#C6241
Cycloheximide	Selleck	Cat#S7418
Bortzomib	MedChemExpress	Cat#HY-10227
Collagenase type I	Worthington	Cat#LS004197
Oil Red O	Sigma-Aldrich	Cat#O1391
BODIPY409/503	Invitrogen	Cat#D3922
DAPI	Beyotime	Cat#1002
Citrate Antigen Retrieval Solution	Beyotime	Cat#P0081

DAB	proteintech	Cat# PR30018
Trizol	Thermo	Cat#15596026
Phosphatase inhibitor cocktail A	Beyotime	Cat#P1082
Phosphatase inhibitor cocktail C	Beyotime	Cat#P1092
Protease inhibitor cocktail	Beyotime	Cat#P1006
N-Ethylmaleimide	Sigma	Cat#E1271
Protein A/G-beads	Beyotime	Cat#P2012
Flag beads	Thermo	Cat# A36797
3×flag peptides	meilunbio	Cat#MB2512
GST beads	yeasen	Cat#20508ES10
IPTG	Beyotime	Cat#ST098
R250	Beyotime	Cat#ST1123
RU486	MCE	Cat#HY-13683
H&E staining kit	Solarbio	Cat#G1120
In Situ β-galactosidase Staining Kit	Beyotime	Cat#RG0039
Chromatin Profile Kit	Novoprotein	Cat#N248-01A
BCA Protein assay kit	Beyotime	Cat# P0009
ECL detection kit	Millipore	Cat#WBKLS0500
ECL detection kit	Epizyme	Cat#SQ202L
EdU	Selleck	Cat#S1661
EdU-488 staining kit	Epizyme	Cat#CX002
MTS	Promega	Cat#G9683
HiScript II Q RT SuperMix for qPCR	Vazyme	Cat#Q711
ChamQ Universal SYBR qPCR Master Mix	Vazyme	Cat#R223-01
AdV5-CMV-Cre-mCMV-copGFP	WZ Bioscience Inc.	Cat#AD201001
AdV5-CMV-C-FH-mCMV-copGFP	WZ Bioscience Inc.	Cat#AD100010-OE

Red Blood Cell Lysing Buffer	Beyotime	Cat#C3702
ALT kit	SSUF	Cat#20152400366
AST kit	SSUF	Cat#20152400367
Serum albumin kit<br>Serum TBil kit	Applygen	Cat#E2062
Serum uric acid kit	Solarbio	Cat#BC5185
Serum urea kit	Solarbio	Cat#BC1365

Supplemental Table 7. Oligonucleotides used in the study.

**shRNA, sgRNA and siRNA sequences**	
sg*Sgk3* (mus)	TGAAGGAATGCTCGGACATC
sh*Sgk3* (mus)	GTTCATGGTATGATCGAAATG
sh*Brg1*-1 (mus)	CCATATTTATACAGCAGAGAA
sh*Brg1*-2 (mus)	GGCATAGGCCTTAGCAGTAAC
si*Nr3c1*-1(mus)	UGAGAUUCGAAUGACUUAUAUTT
si*Nr3c1*-2 (mus)	GGAUAAGUCCAUGAGUAUUGTT
**RT-qPCR primers**	
Sgk3 (mus)-F	CTTCACCGGCAGGAGAGTG
Sgk3 (mus)-R	GGCAGGAATCTTCAGAGCCATA
Pparg (mus)-F	TCGCTGATGCACTGCCTATG
Pparg (mus)-R	GAGAGGTCCACAGAGCTGATT
Cebpa (mus)-F	CAAGAACAGCAACGAGTACCG
Cebpa (mus)-R	GTCACTGGTCAACTCCAGCAC
Adipoq (mus)-F	TGACGACACCAAAAGGGCTC
Adipoq (mus)-R	ACCTGCACAAGTTCCCTTGG
Nr3c1 (mus)-F	AGCTCCCCCTGGTAGAGAC
Nr3c1 (mus)-R	GGTGAAGACGCAGAAACCTTG
Sgk1 (mus)-F	CTGCTCGAAGCACCCTTACC
Sgk1 (mus)-R	TCCTGAGGATGGGACATTTTCA
Rasd1 (mus)-F	CGCCTCTCTATCCTCACAGG
Rasd1 (mus)-R	GGTCCAAGCTGCTGTTCTTC
Brg1 (mus)-F	CAAAGACAAGCATATCCTAGCCA
Brg1 (mus)-R	CACGTAGTGTGTGTTAAGGACC
Catalase (mus)-F	AGCGACCAGATGAAGCAGTG

Catalase (mus)-R	TCCGCTCTCTGTCAAAGTGTG
Actb (mus)-F	GGCTGTATTCCCCTCCATCG
Actb (mus)-R	CCAGTTGGTAACAATGCCATGT
