# Supplementary material for: Serum- and glucocorticoid-induced kinase 3 orchestrates glucocorticoid signaling to facilitate chromatin remodeling during murine adipogenesis
Source: J Clin Invest. 2025 Jul 24;135(19):e186534. doi: 10.1172/JCI186534 (PMC12483569; doi:10.1172/JCI186534)

Full unedited blot for Figure 1

Fig 1B

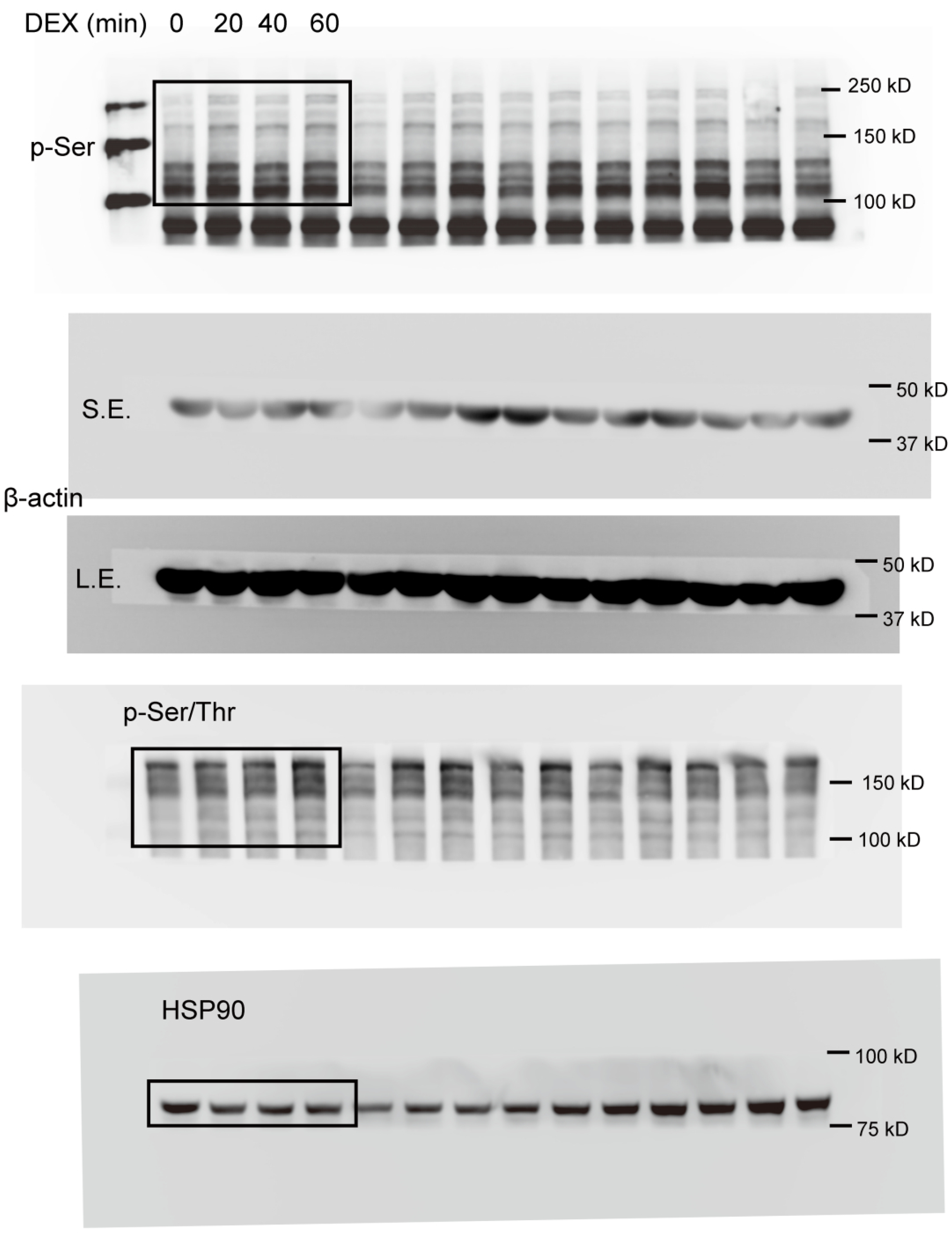

Identical sample aliquots were loaded across multiple gels.
HSP90 (serving as loading control) and p-Ser/Thr were immunoblotted from a single gel, whereas p-Ser and β-actin (alternative loading control) were analyzed on a parallel gel.

Fig 1C

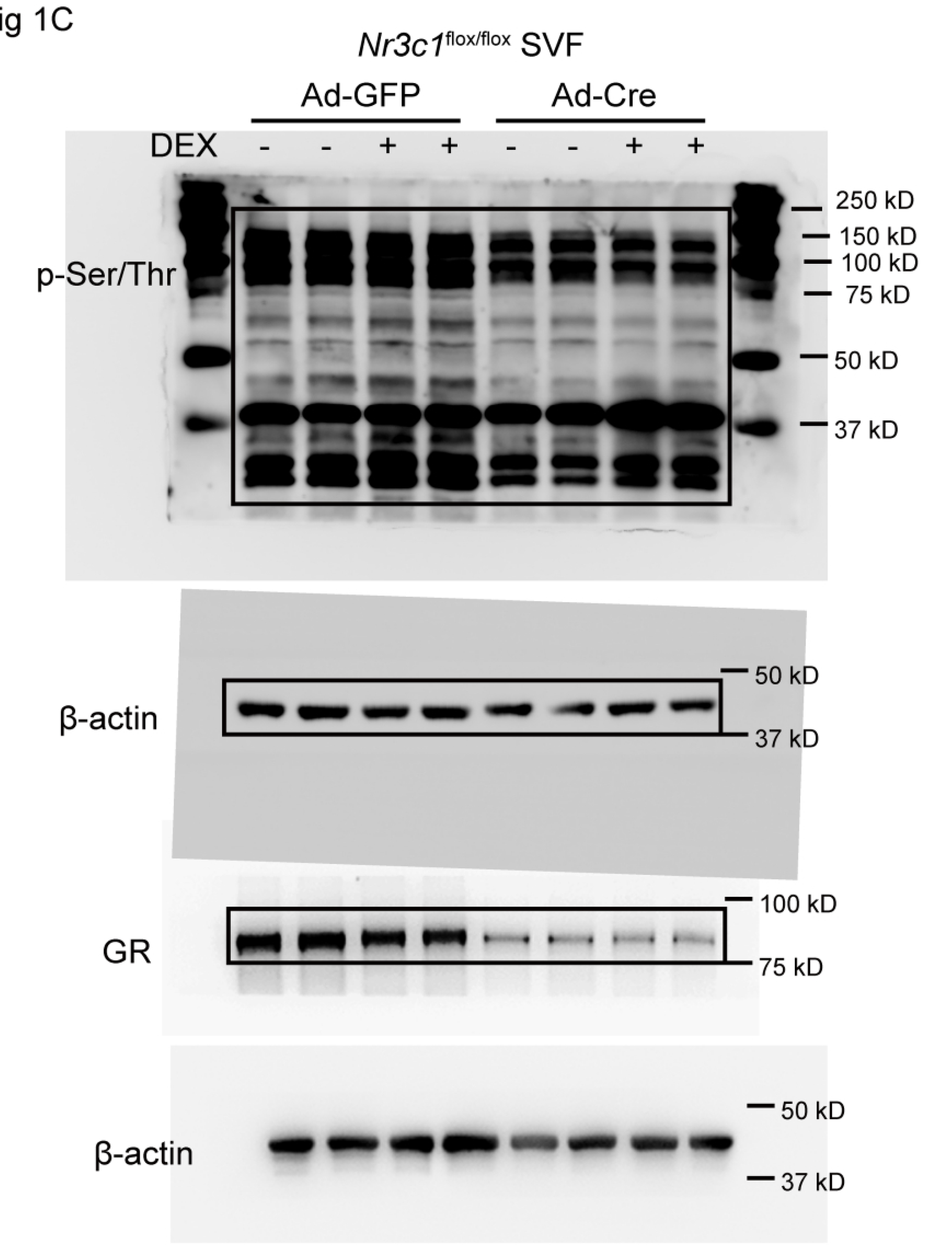

Identical sample aliquots were loaded across multiple gels.
β-actin (upper, serving as loading control) and p-Ser/Thr were immunoblotted from a single gel, whereas GR and β-actin (lower,alternative loading control) were analyzed on a parallel gel.

Fig 1H

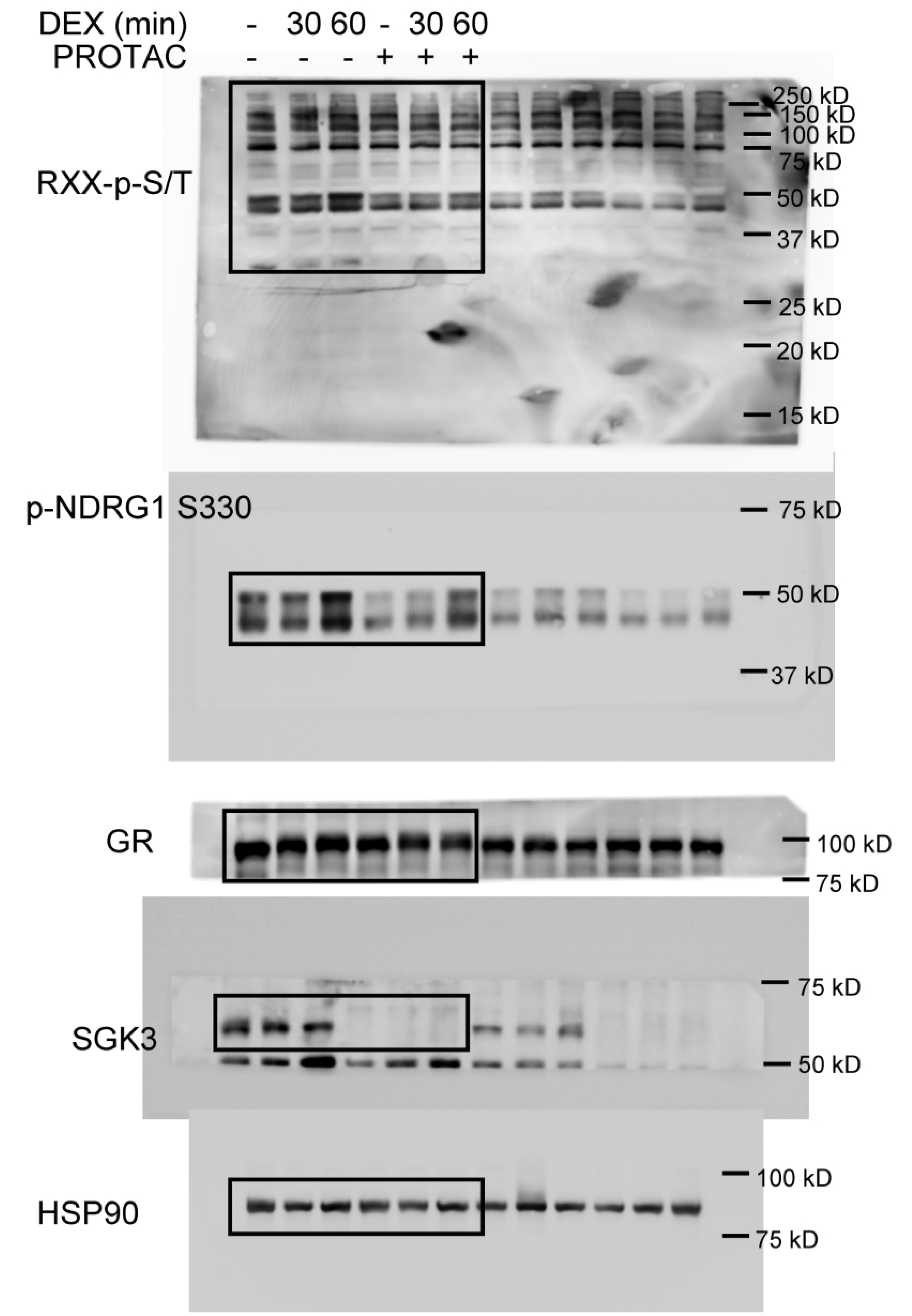

Identical sample aliquots were loaded across multiple gels.
HSP90 (serving as loading control) and SGK3 were immunoblotted from a single gel, whereas ohters were analyzed on parallel gels (no additional loading control detected).

Full unedited blot for Figure 3

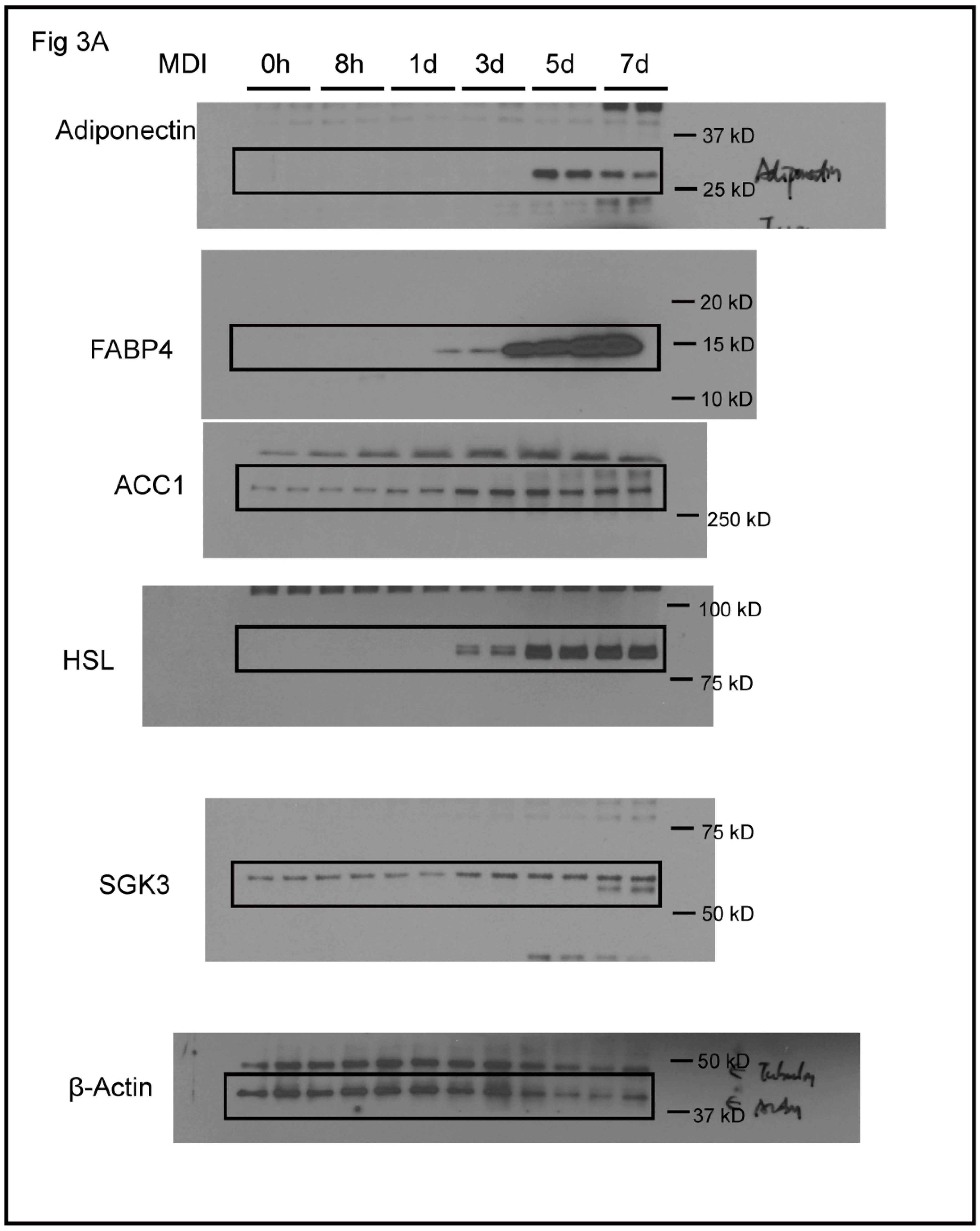

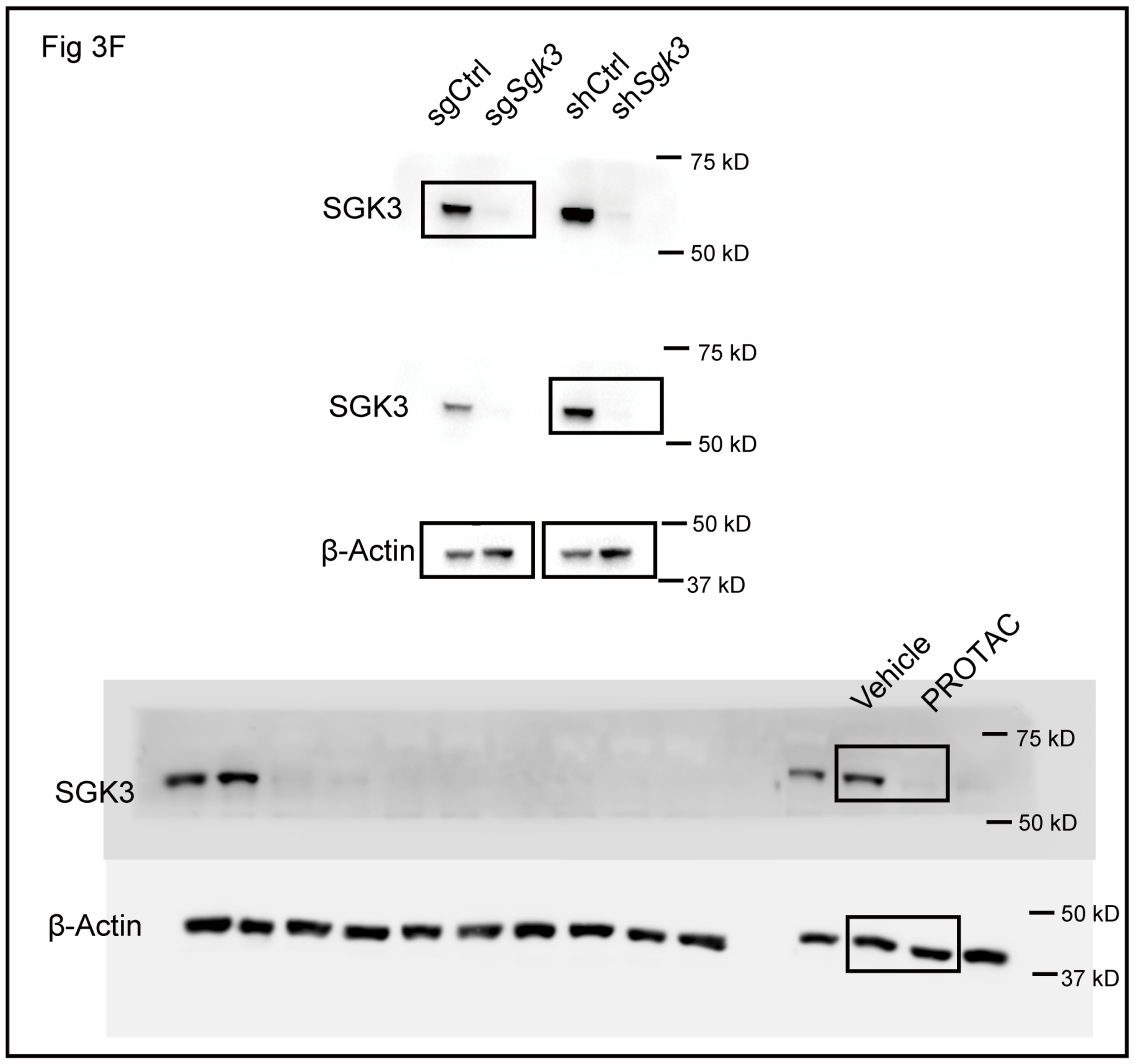

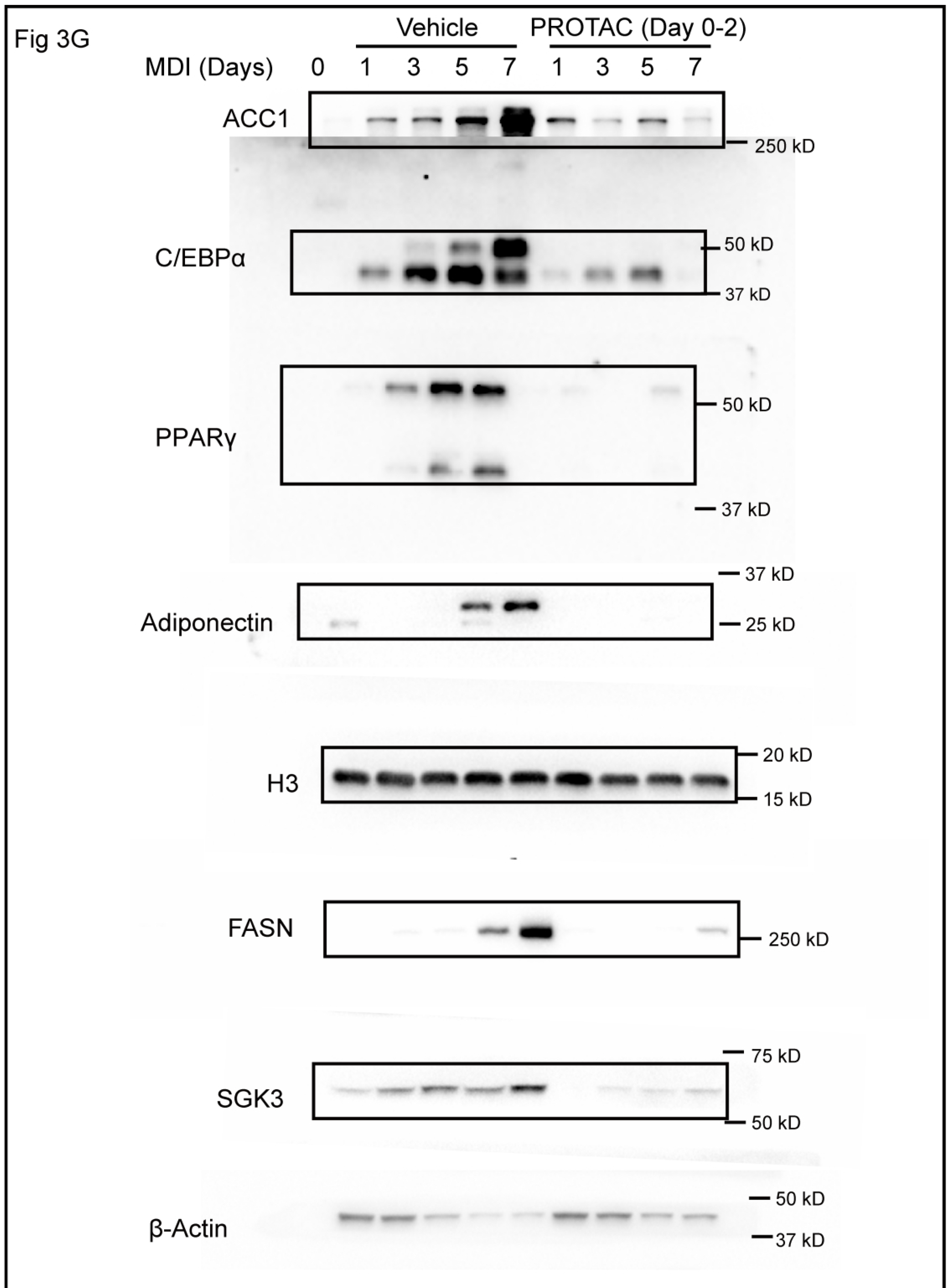

Identical sample aliquots were loaded across multiple gels.
H3 (serving as loading control) and Adiponectin were immunoblotted from a single gel, SGK3, FASN and β-Actin were immunoblotted from a single gel, whereas others were analyzed on parallel gels (no additional loading control detected).

Full unedited blot for Figure 4

Fig 4G

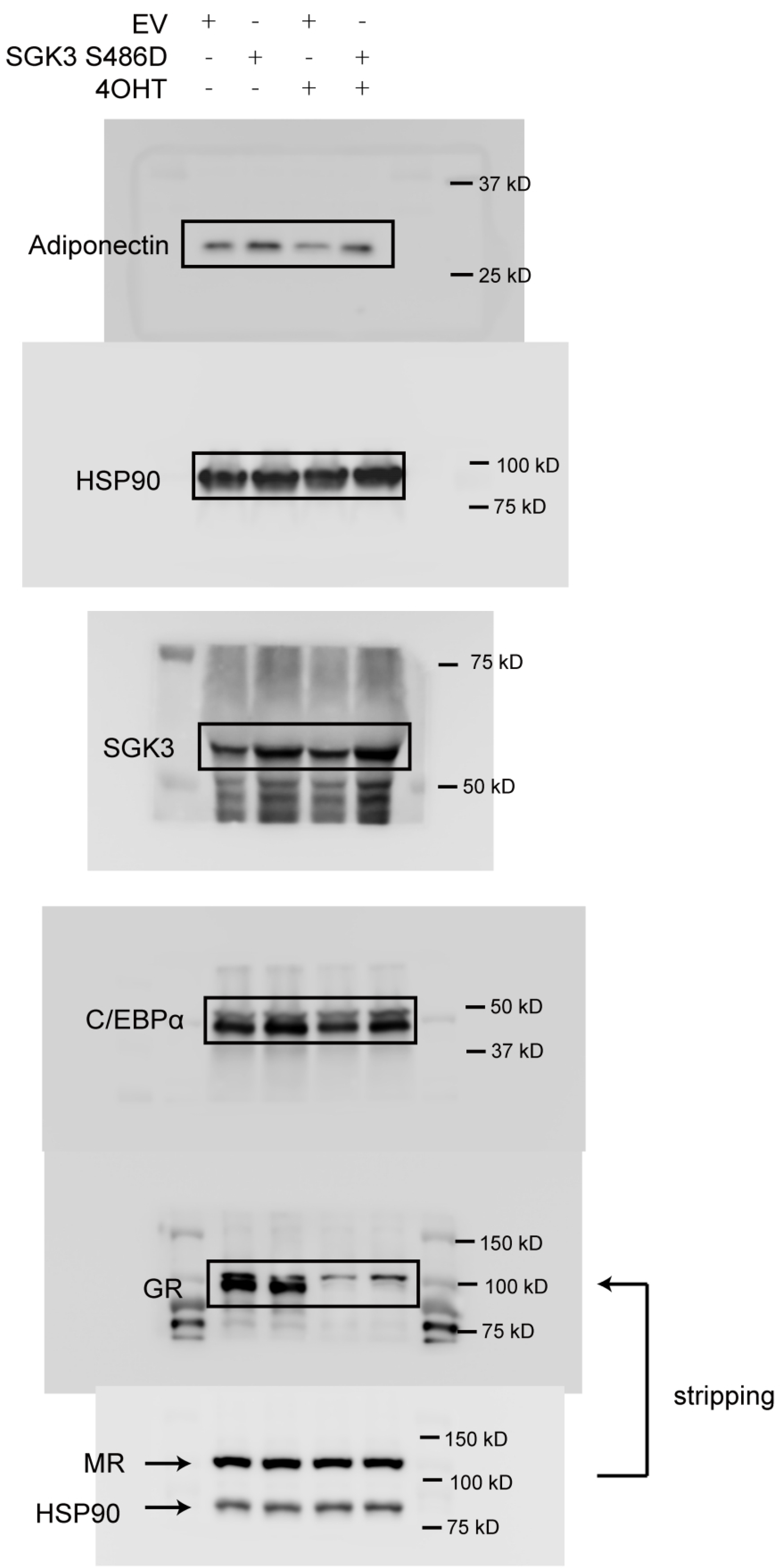

Identical sample aliquots were loaded across multiple gels.
HSP90 (serving as loading control) and Adiponectin were immunoblotted from a single gel, HSP90 (lower, alternative loading control) GR and C/EBPα were immunoblotted from a single gel, whereas SGK3 was analyzed on a parallel gel (no additional loading control detected).

Full unedited blot for Figure 5

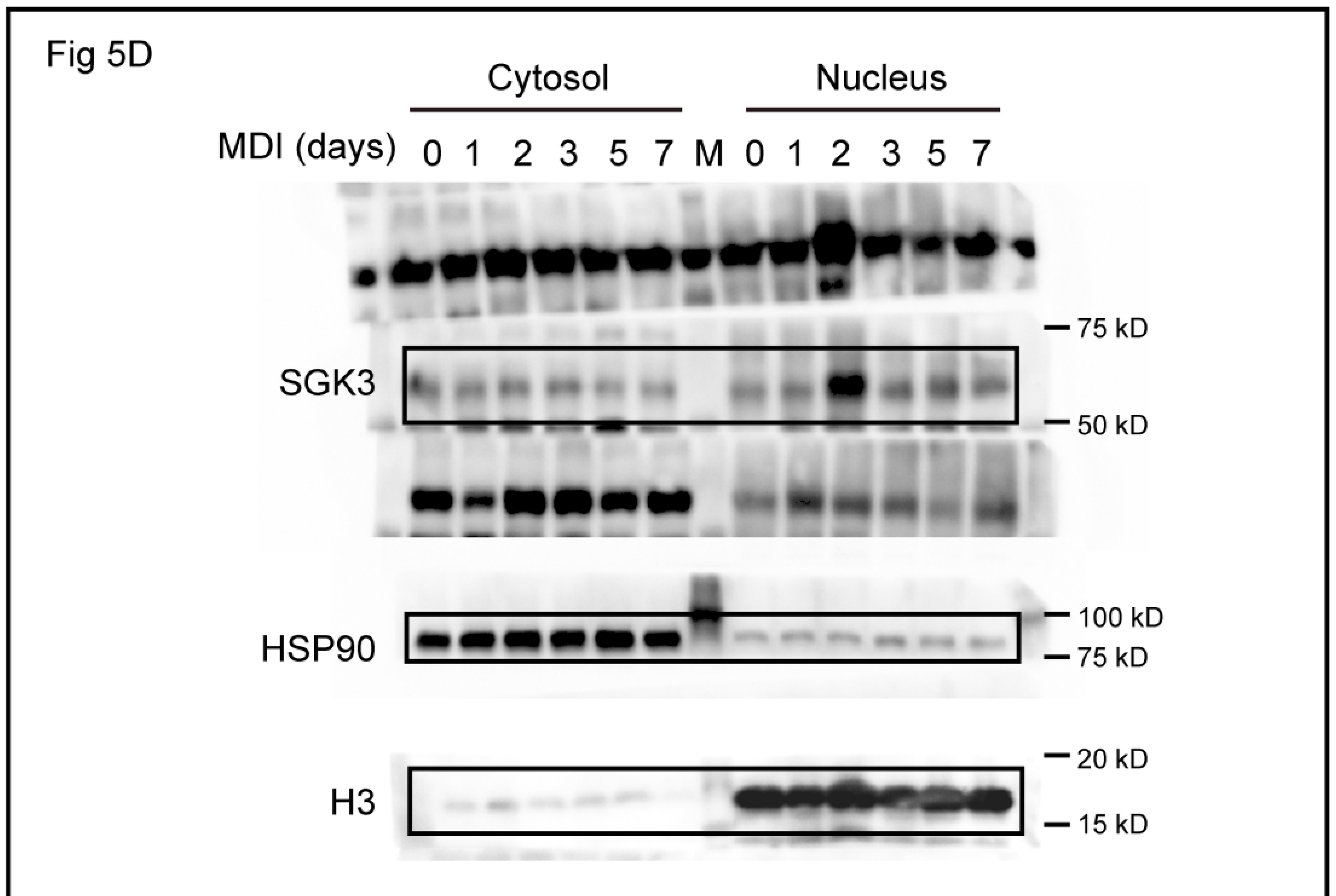

Identical sample aliquots were loaded across multiple gels.
HSP90 (serving as cytosol marker) and SGK3 were immunoblotted from a single gel, whereas H3 (serving as nucleus marker) were analyzed on a parallel gel.

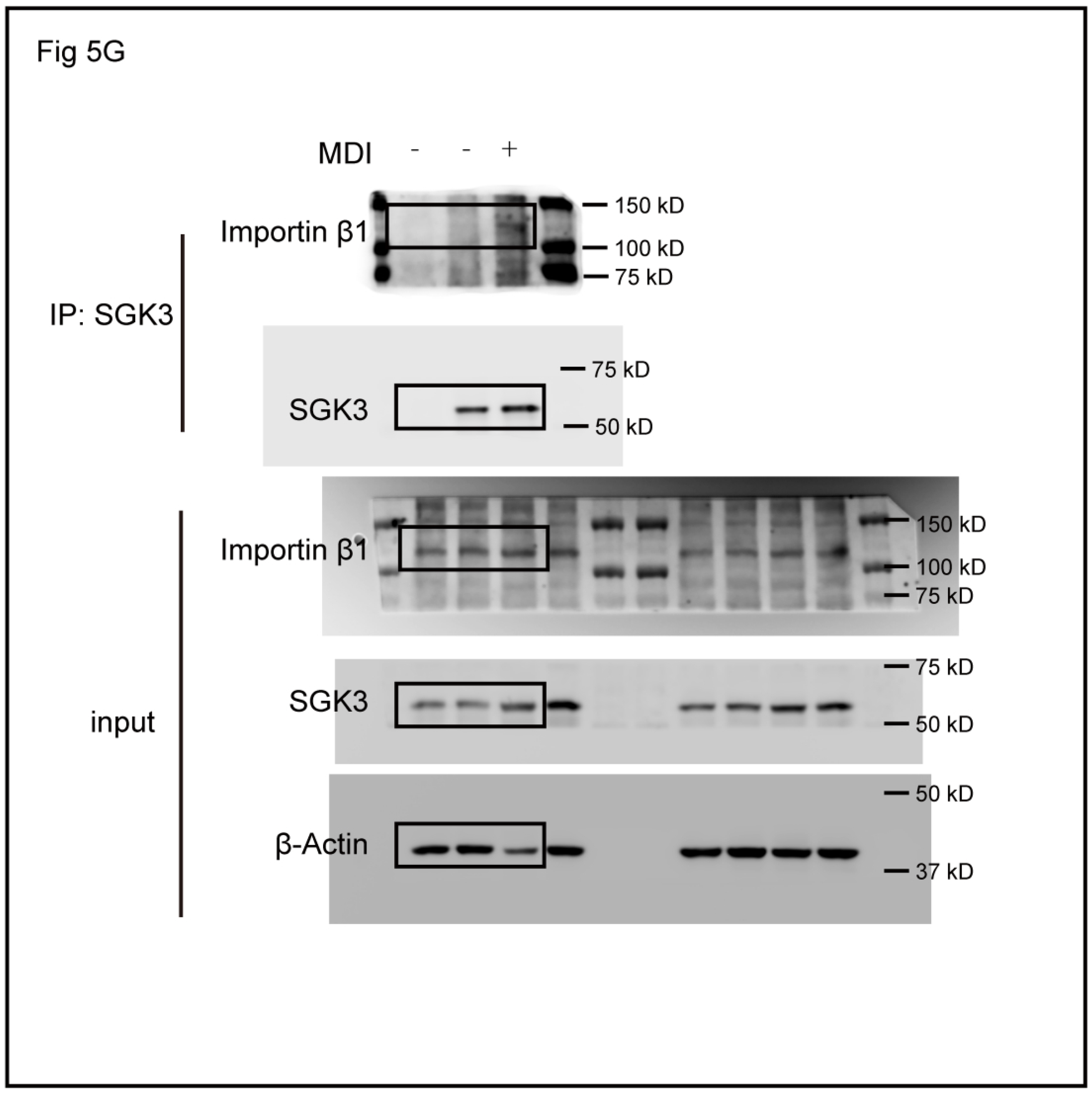

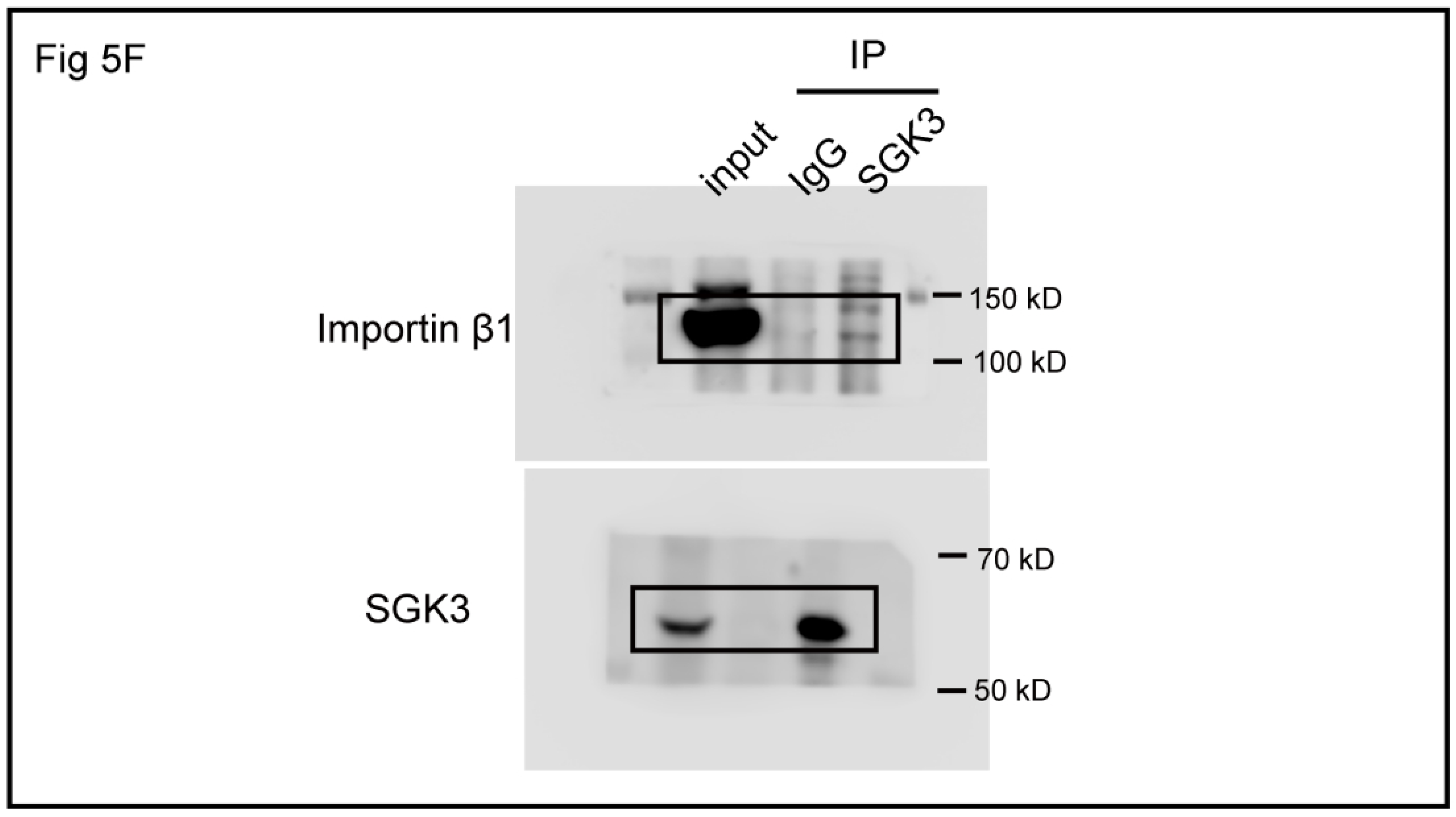

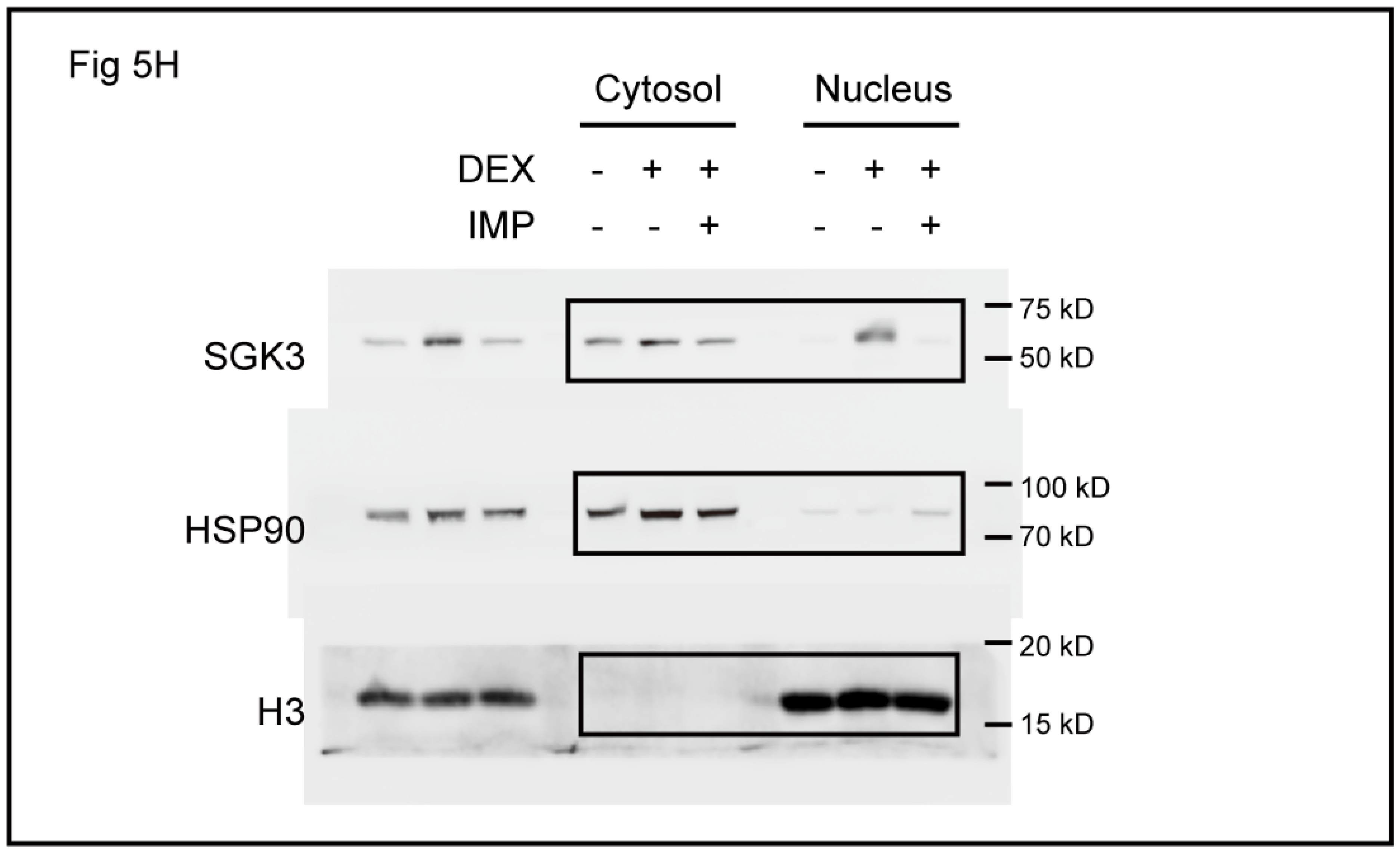

Full unedited blot for Figure 6

Fig 6A

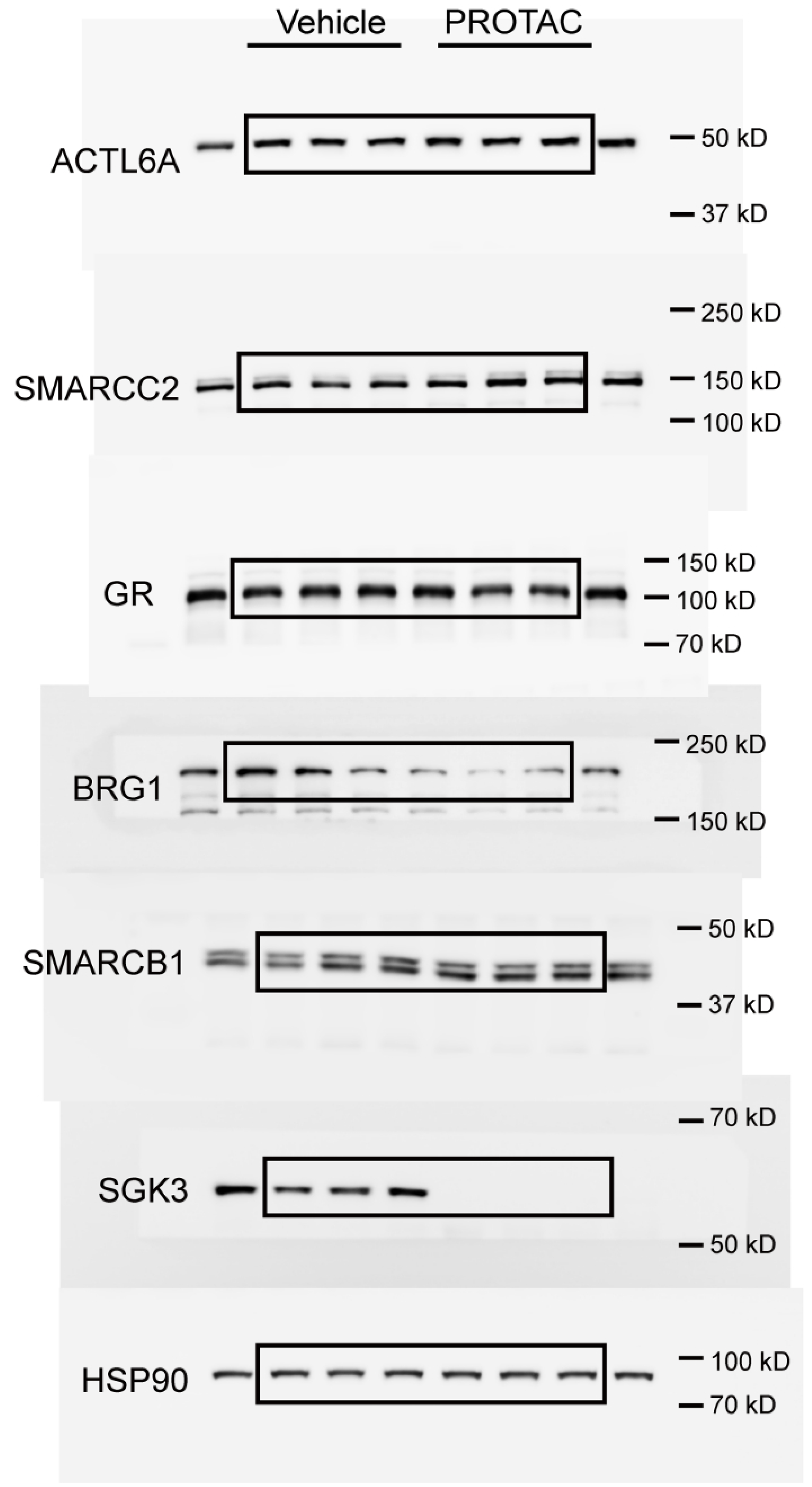

Identical sample aliquots were loaded across multiple gels.
HSP90 (serving as loading control), BRG1, SGK3 and SMARCB1 were immunoblotted from a single gel, whereas others were analyzed on parallel gels (no additional loading control detected).

Full unedited blot for Figure 7

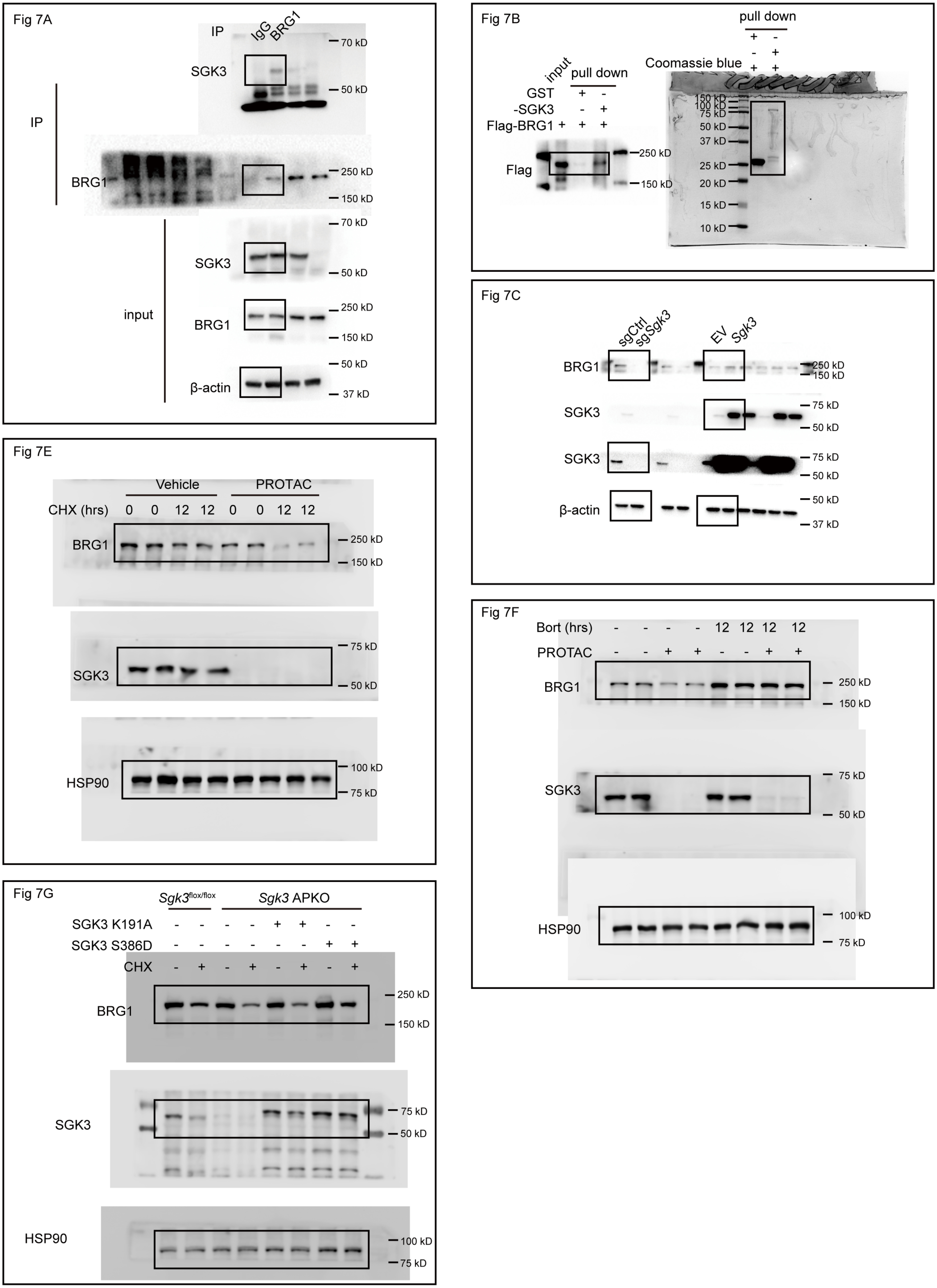

Full unedited blot for Figure 7

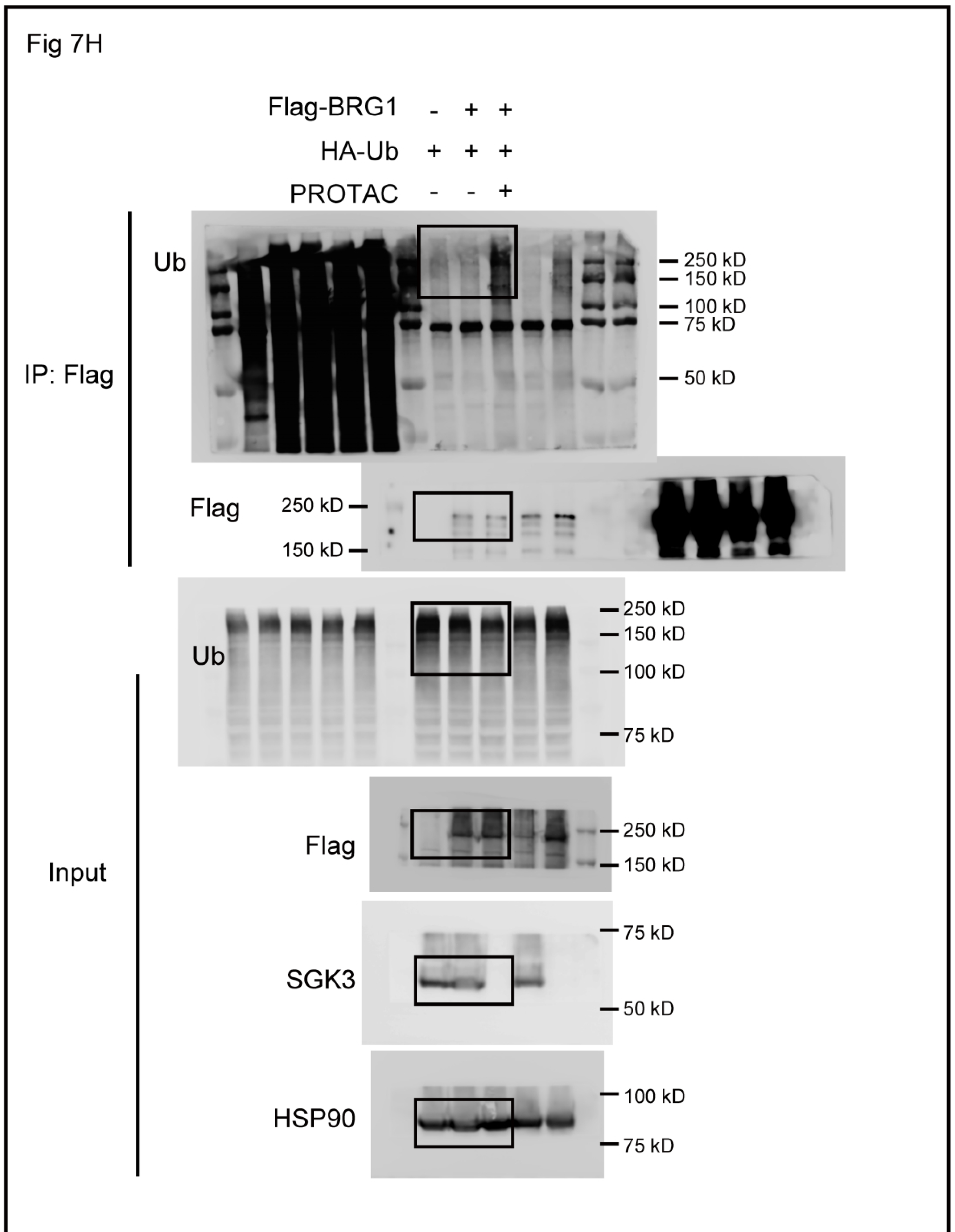

Identical sample aliquots were loaded across multiple gels.
HSP90 (serving as loading control), SGK3 and Flag (input) were immunoblotted from a single gel, whereas others were analyzed on parallel gels.

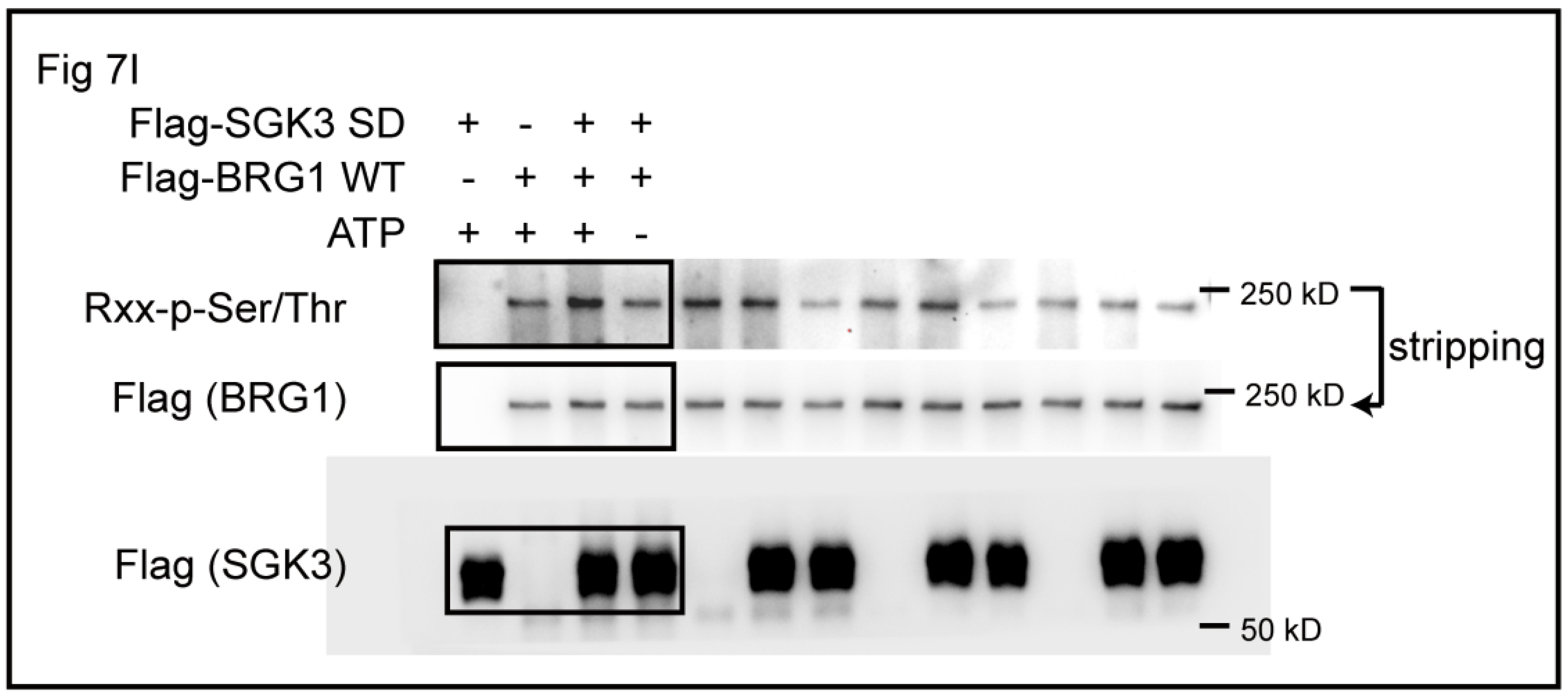

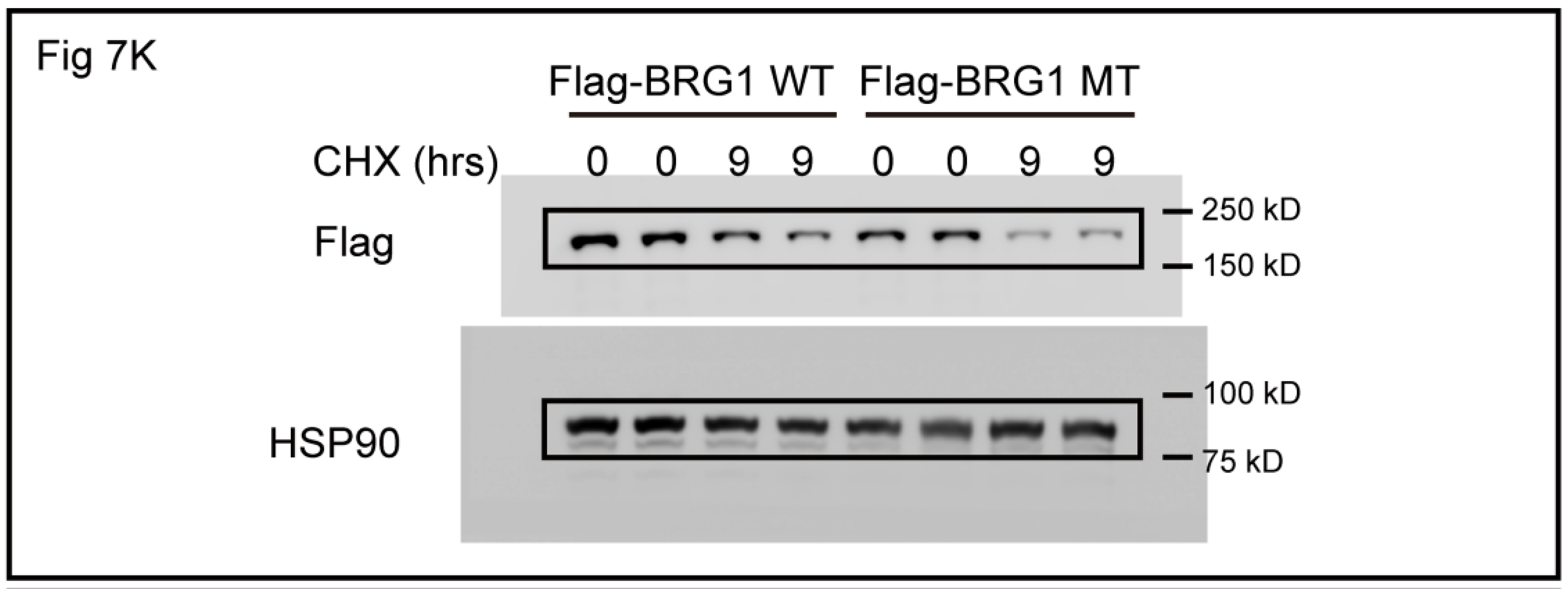

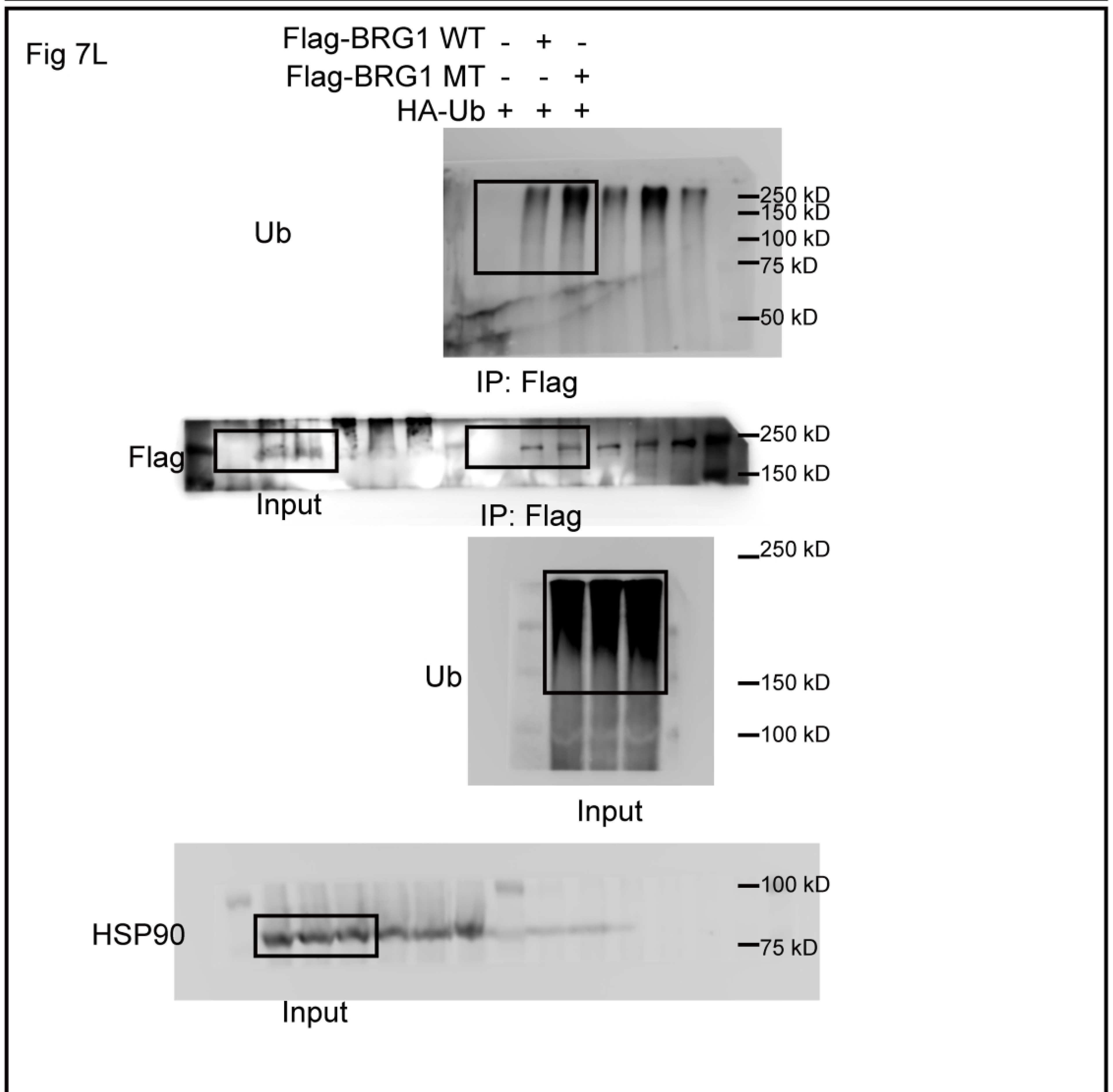

Identical sample aliquots were loaded across multiple gels.
HSP90 (serving as loading control) and Flag were immunoblotted from a single gel, whereas others were analyzed on parallel gels.

Full unedited blot for Figure 8

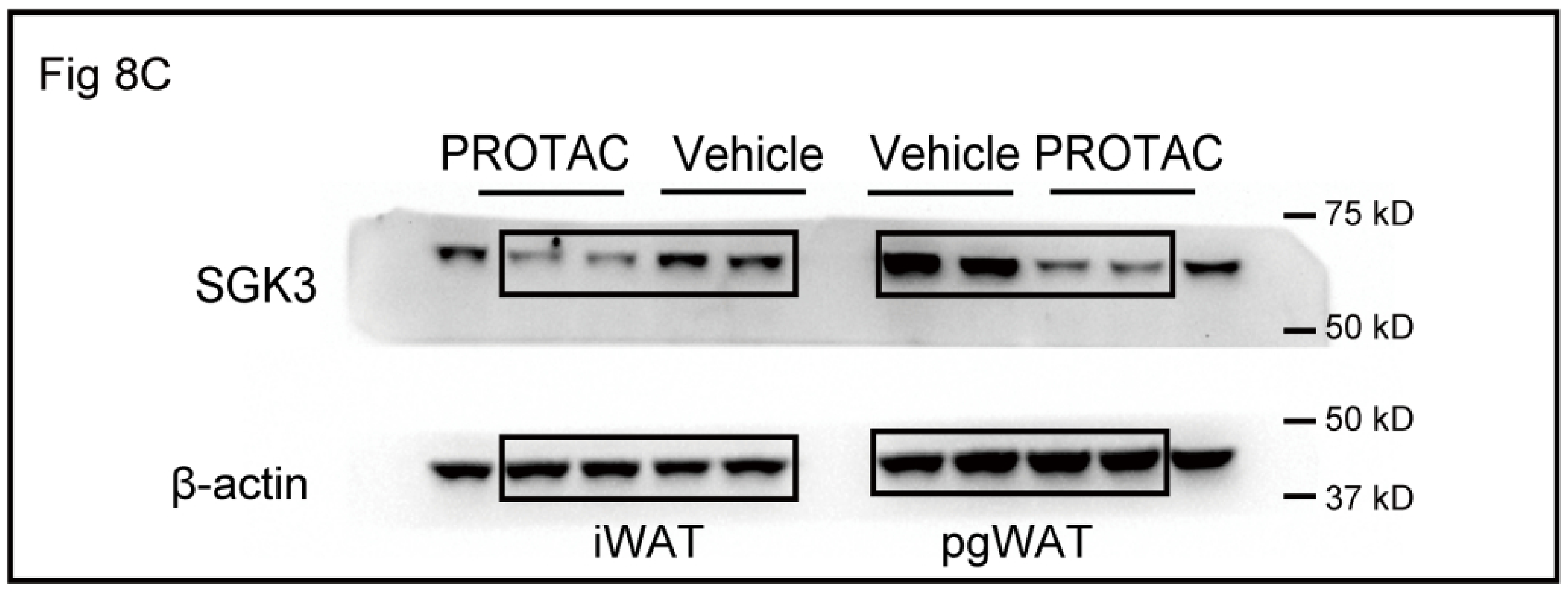

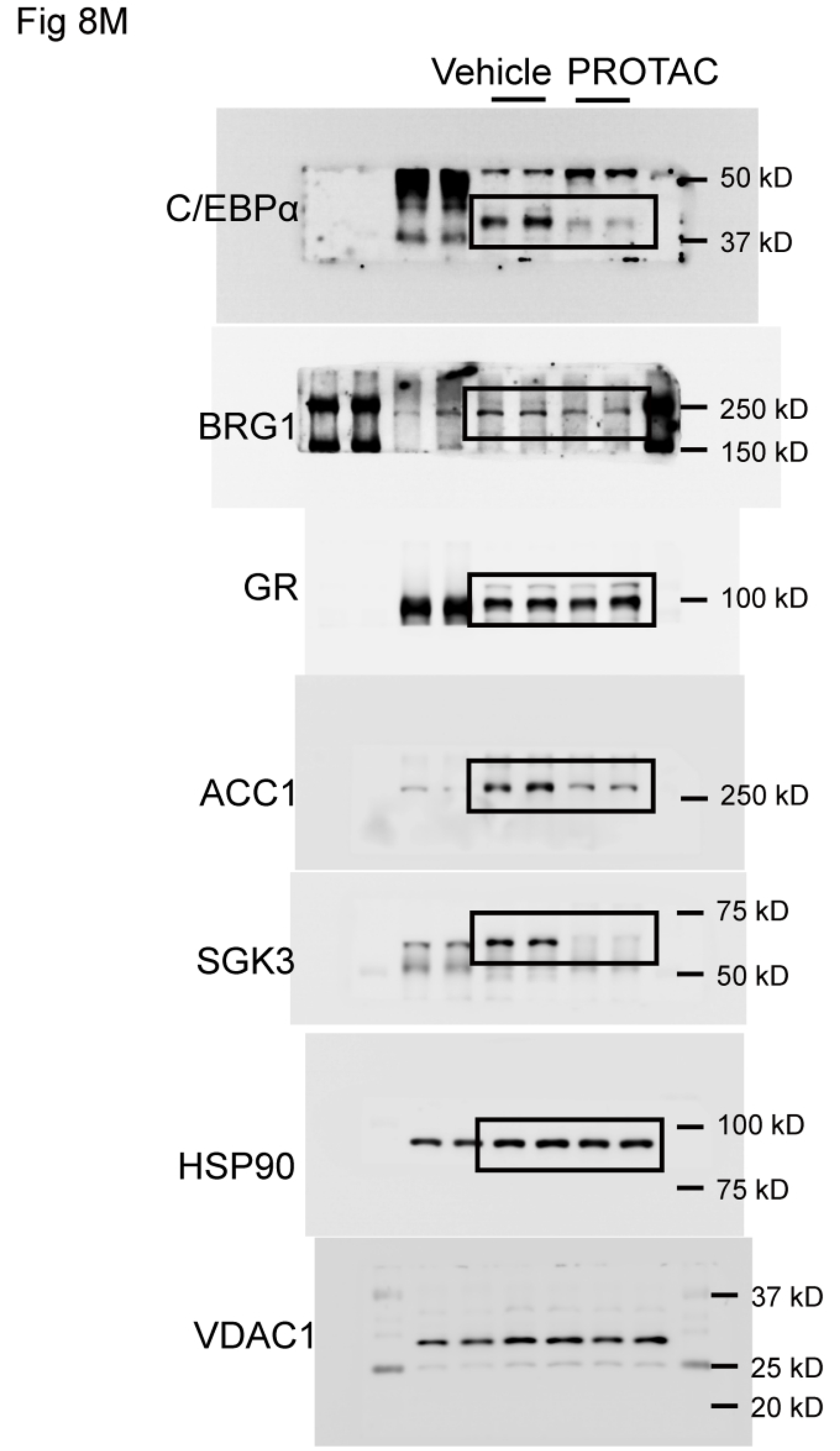

Identical sample aliquots were loaded across multiple gels.
HSP90 (serving as loading control), VDAC1 (alternative loading control), SGK3 and ACC1 were immunoblotted from a single gel, whereas others were analyzed on a parallel gel (no additional loading control detected).

Full unedited blot for Figure S2

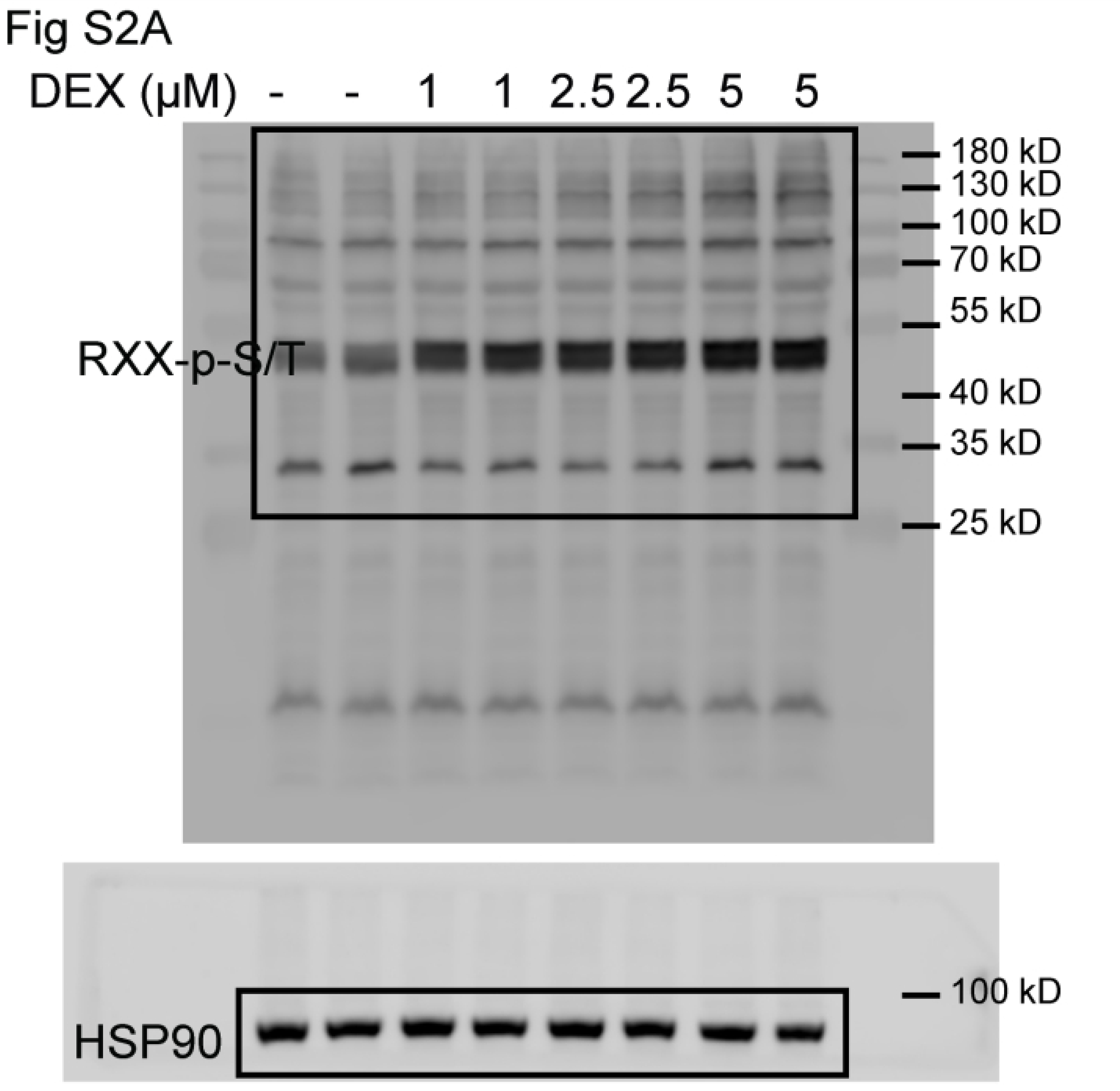

Identical sample aliquots were loaded across multiple gels. HSP90 (serving as loading control) was analyzed on a parallel gel.

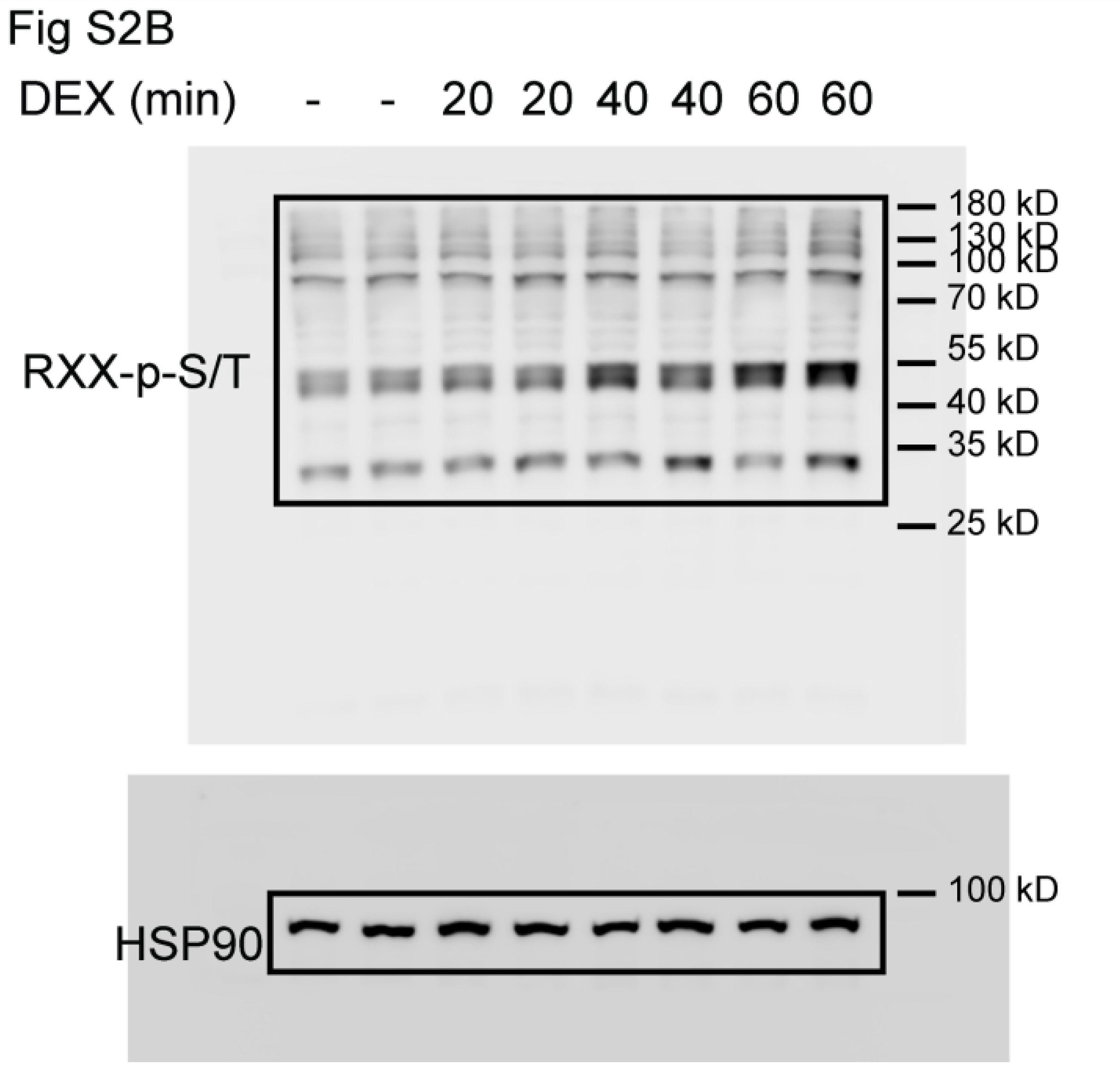

Identical sample aliquots were loaded across multiple gels. HSP90 (serving as loading control) was analyzed on a parallel gel.

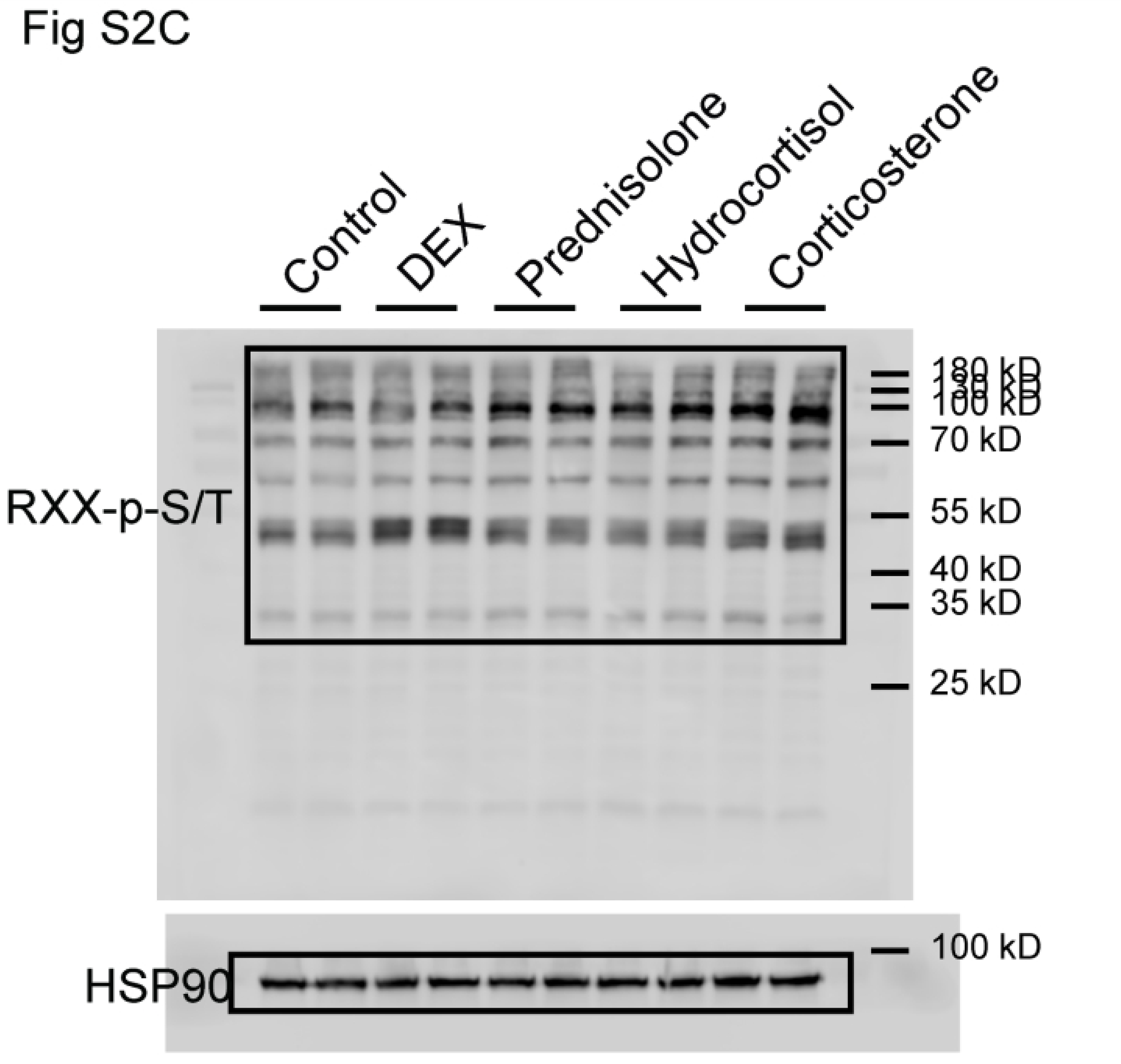

Identical sample aliquots were loaded across multiple gels. HSP90 (serving as loading control) was analyzed on a parallel gel.

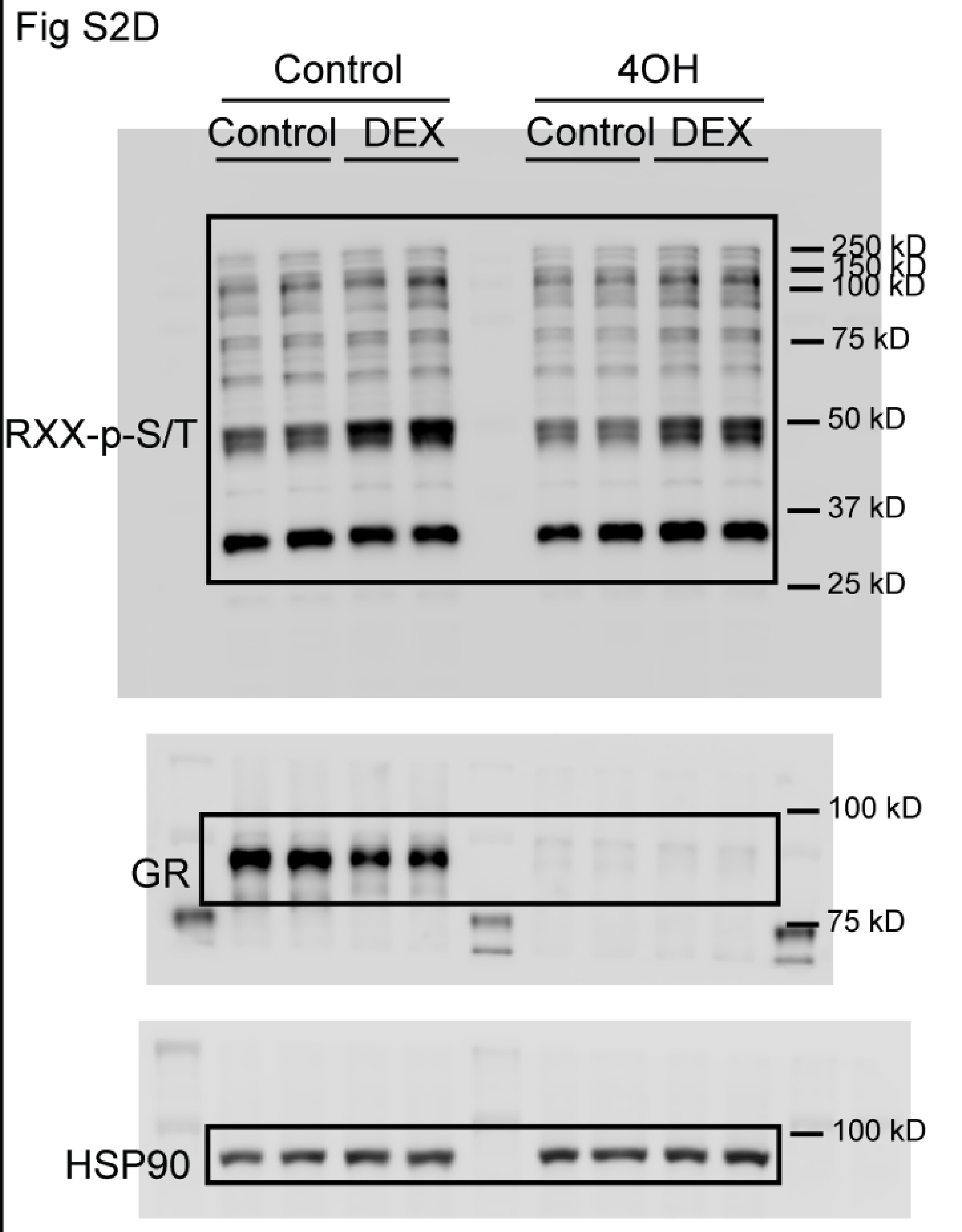

Identical sample aliquots were loaded across multiple gels. HSP90 (serving as loading control) and GR was analyzed on a parallel gel.

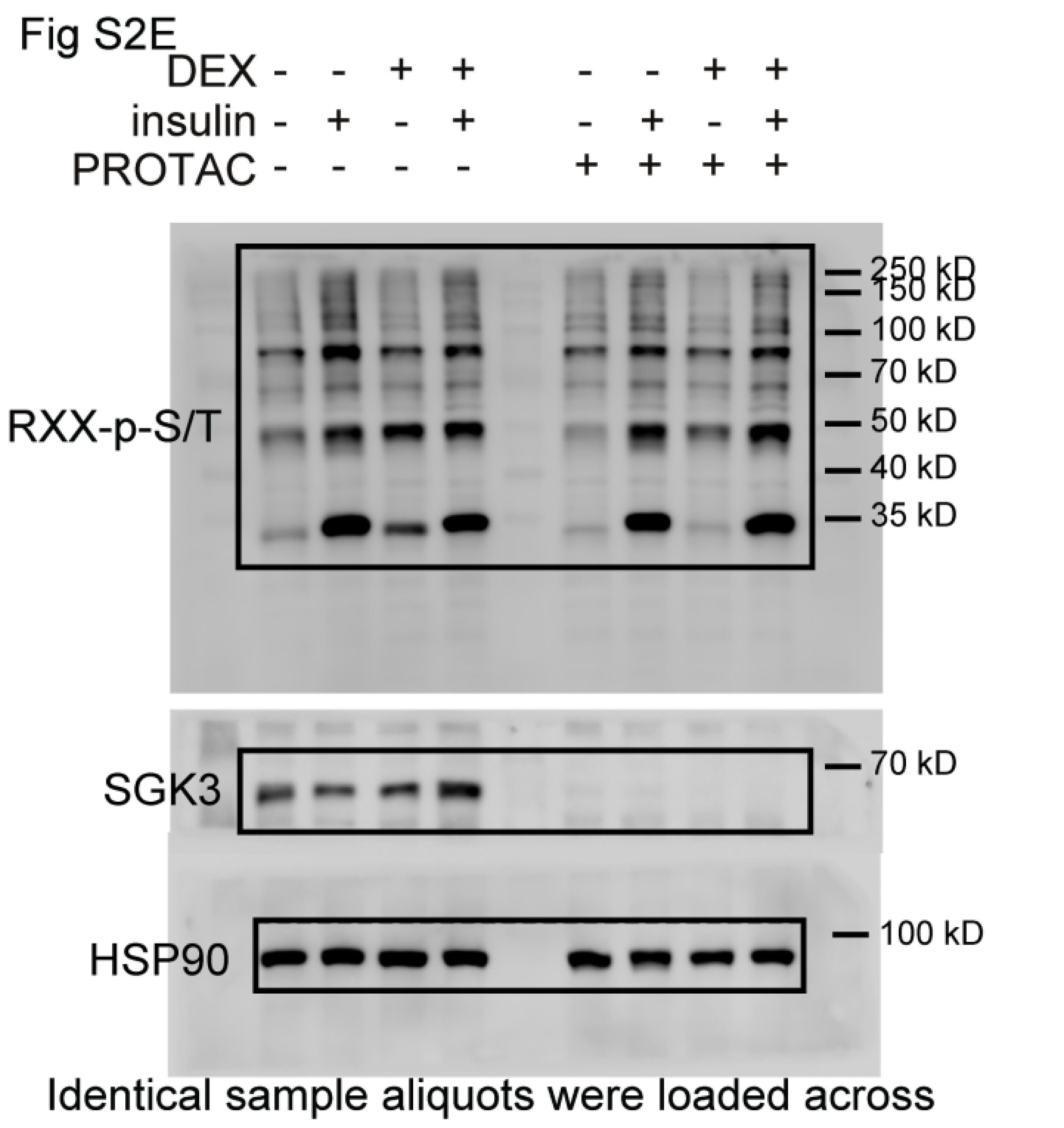

Identical sample aliquots were loaded across multiple gels. HSP90 (serving as loading control) and SGK3 were immunoblotted from a single gel, whereas RXX-p-S/T was analyzed on a parallel gel (no additional loading control detected).

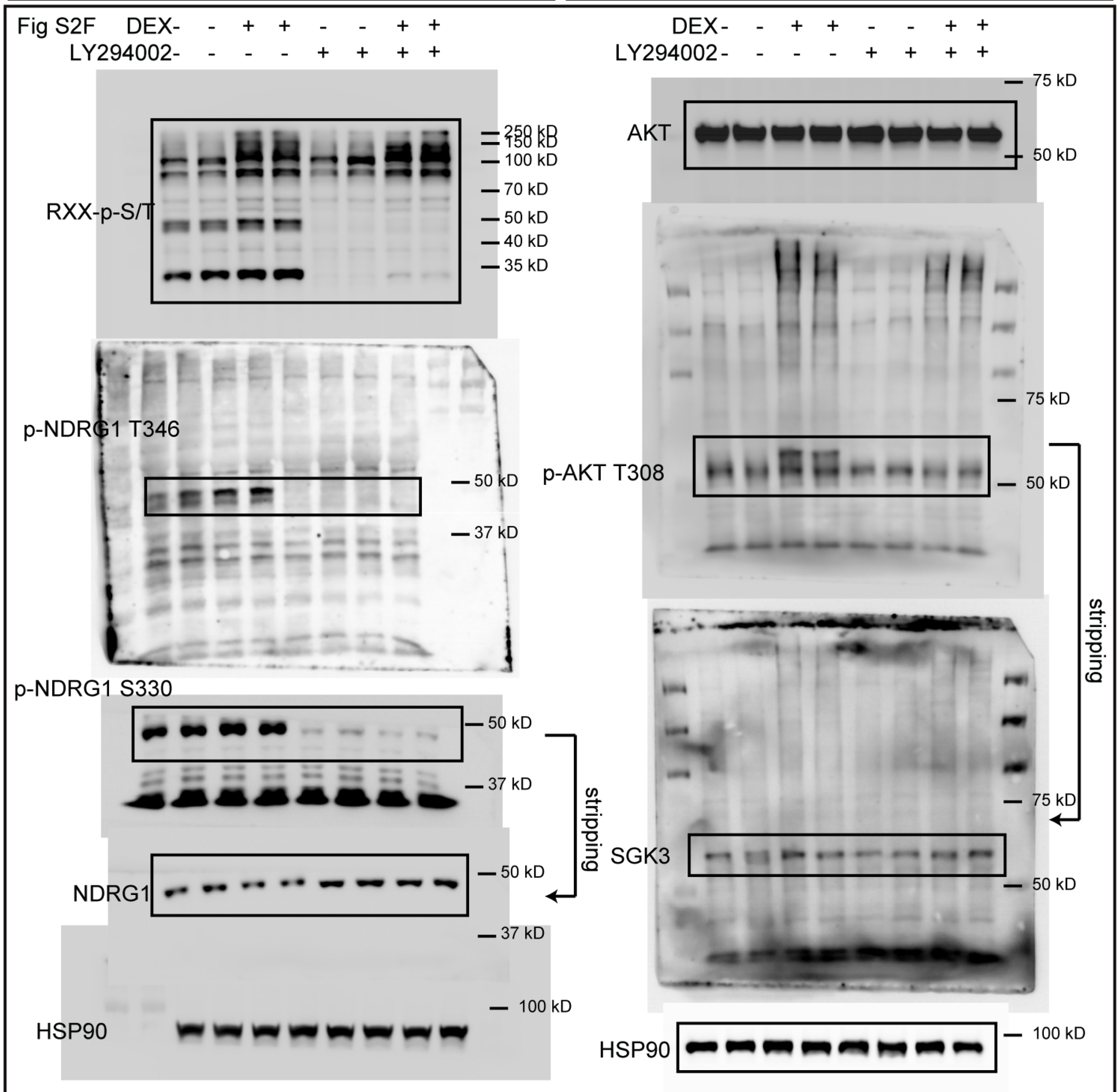

Identical sample aliquots were loaded across multiple gels.
HSP90 (right, serving as loading control) p-AKT T308 and SGK3 were immunoblotted from a single gel, HSP90 (left, alternative loading control), p-NDRG1 S330 and NDRG1 were immublotted from a single gel, whereas RXX-p-S/T, p-NDRG1 T346 and AKT were detected in parallel gels (no additional loading control detected).

Full unedited blot for Figure S5

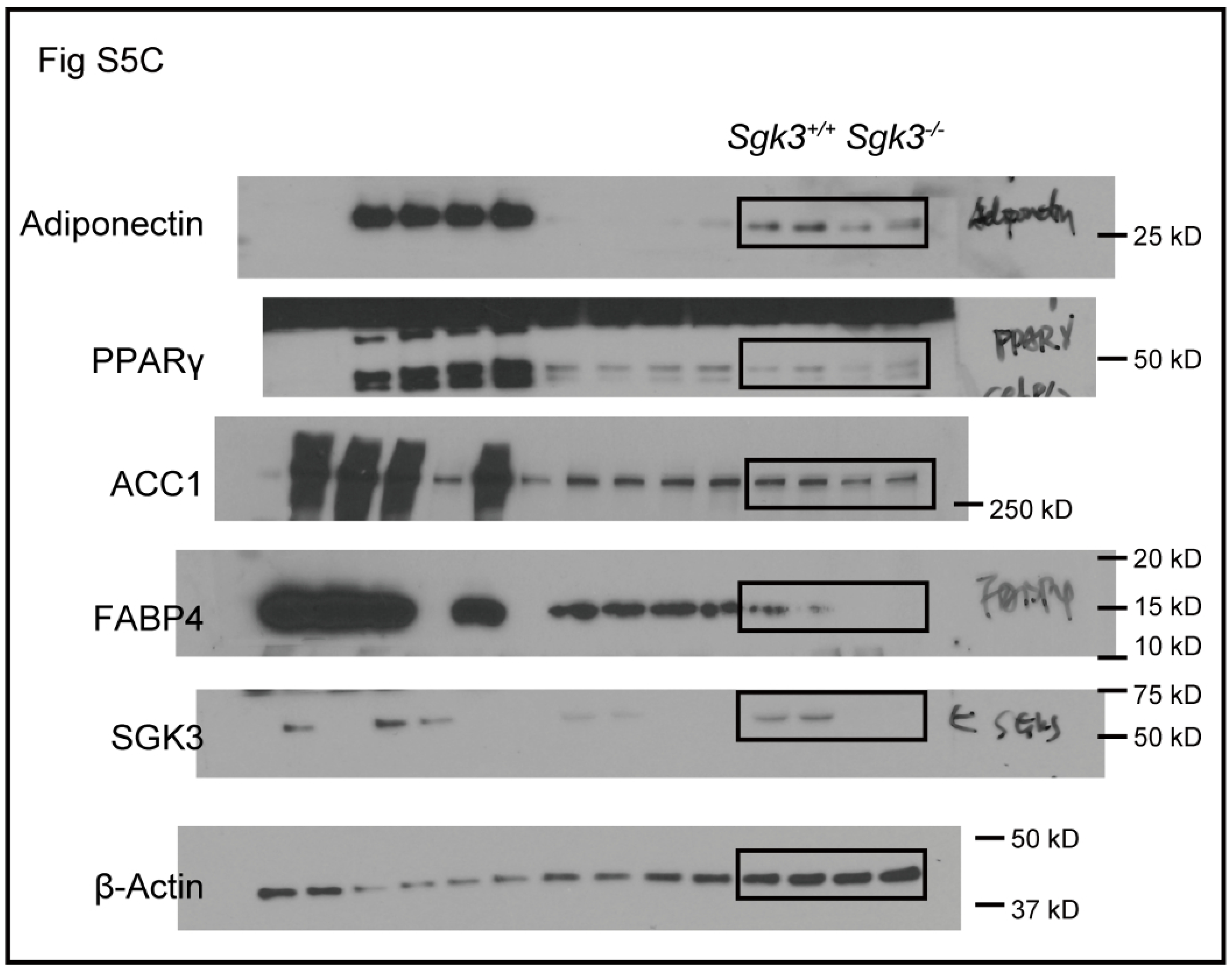

Full unedited blot for Figure S6

Fig S6D

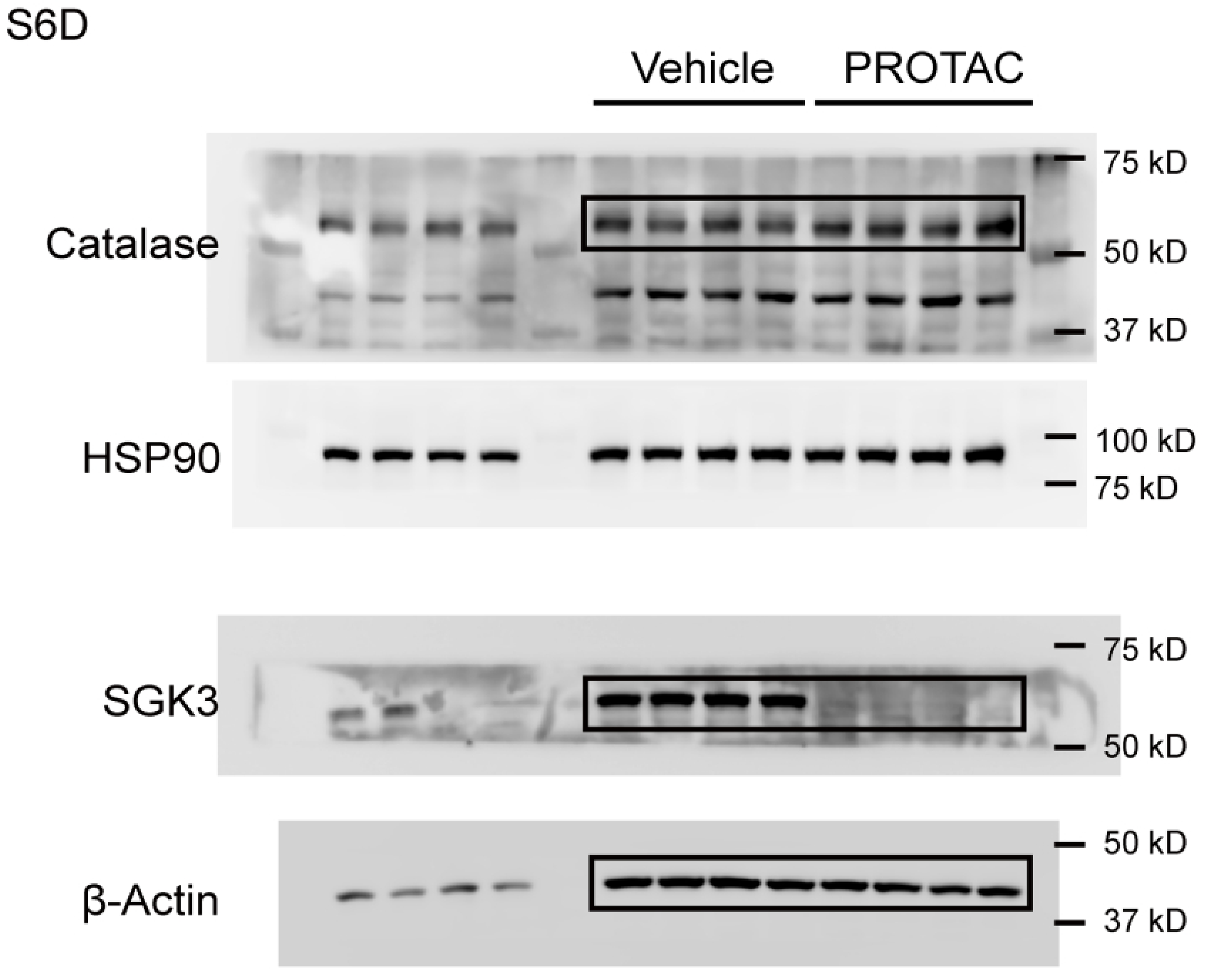

Identical sample aliquots were loaded across multiple gels.
β-Actin (serving as loading control) and SGK3 were immunoblotted from a single gel, whereas HSP90 (alternative loading control) and Catalase were analyzed on parallel gels.

Fig S6E

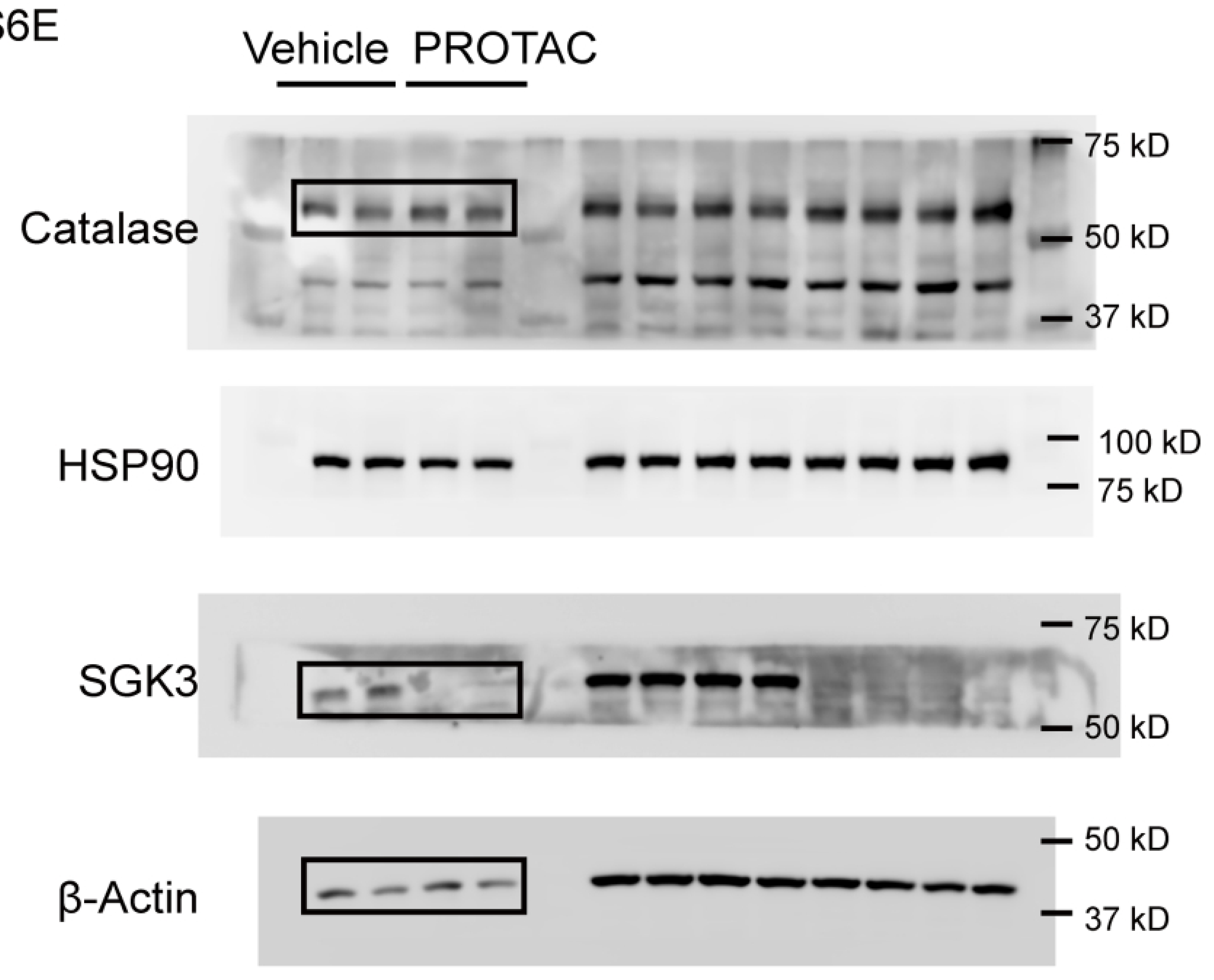

Identical sample aliquots were loaded across multiple gels.
β-Actin (serving as loading control) and SGK3 were immunoblotted from a single gel, whereas HSP90 (alternative loading control) and Catalase were analyzed on parallel gels.

Fig S6F

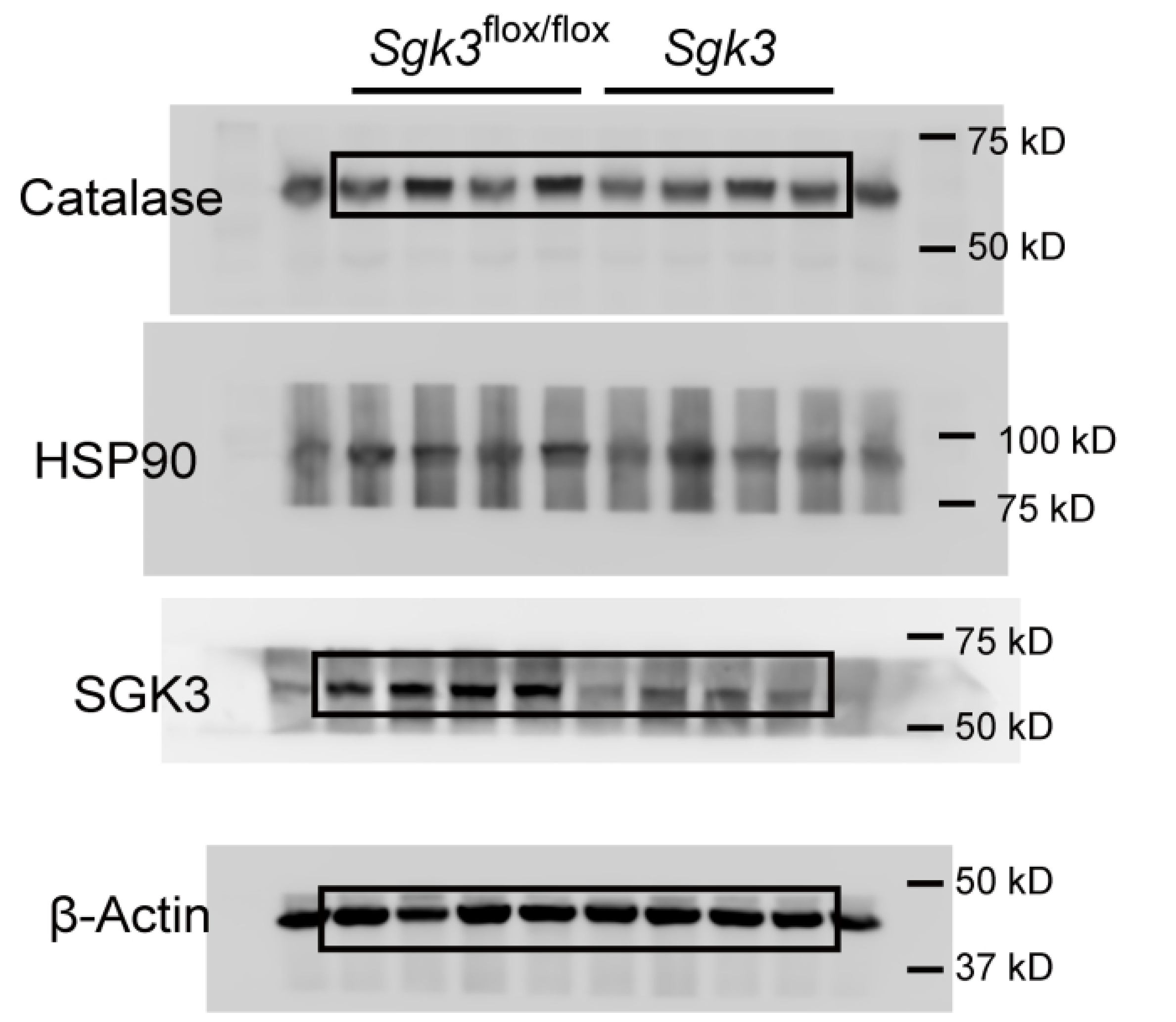

Identical sample aliquots were loaded across multiple gels.
β-Actin (serving as loading control) and SGK3 were immunoblotted from a single gel, whereas HSP90 (alternative loading control) and Catalase were analyzed on parallel gels.

Full unedited blot for Figure S7

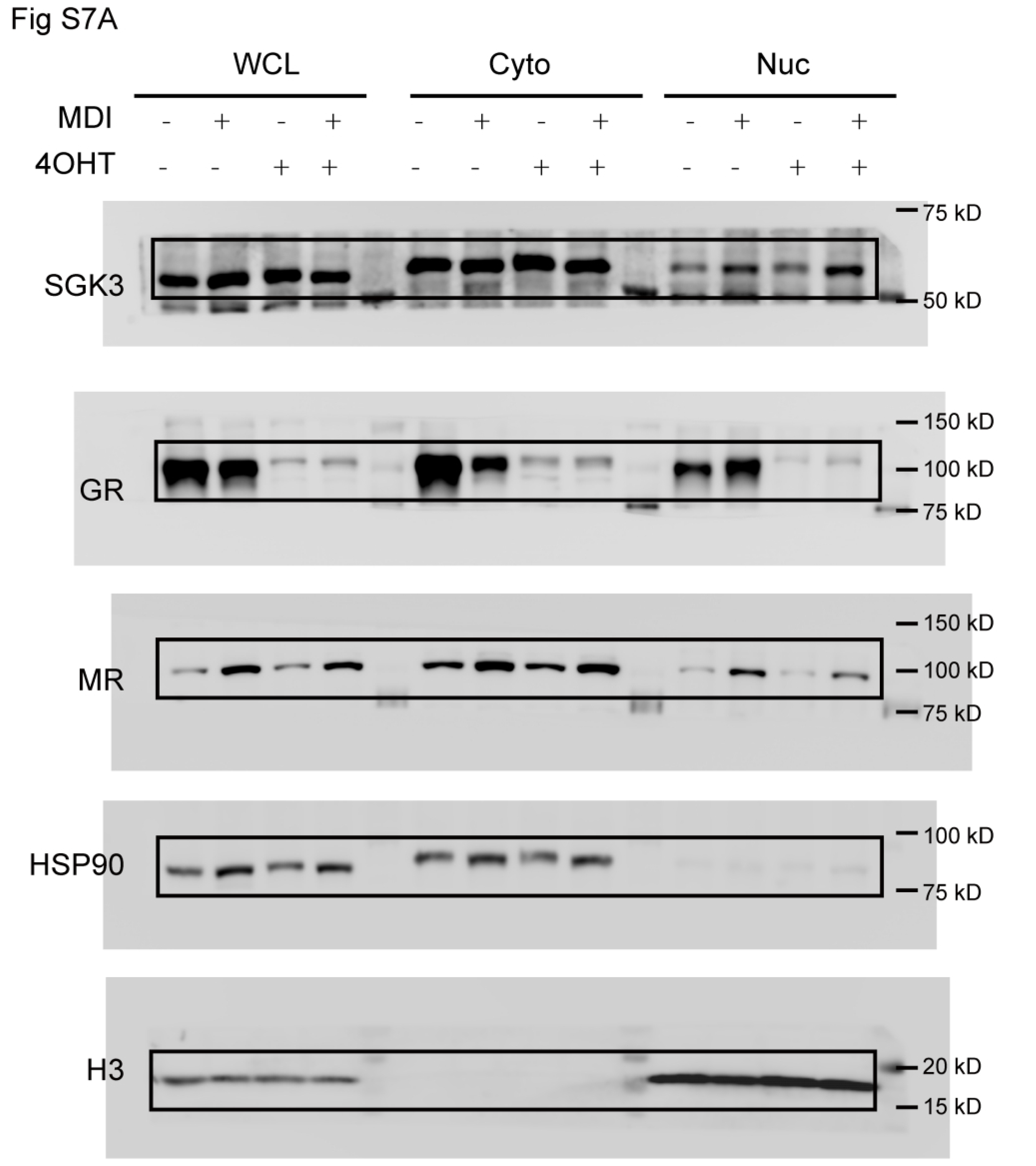

Identical sample aliquots were loaded across multiple gels.
HSP90 (serving as cytosol marker) and SGK3 were immunoblotted from a single gel, whereas others were analyzed on parallel gels.

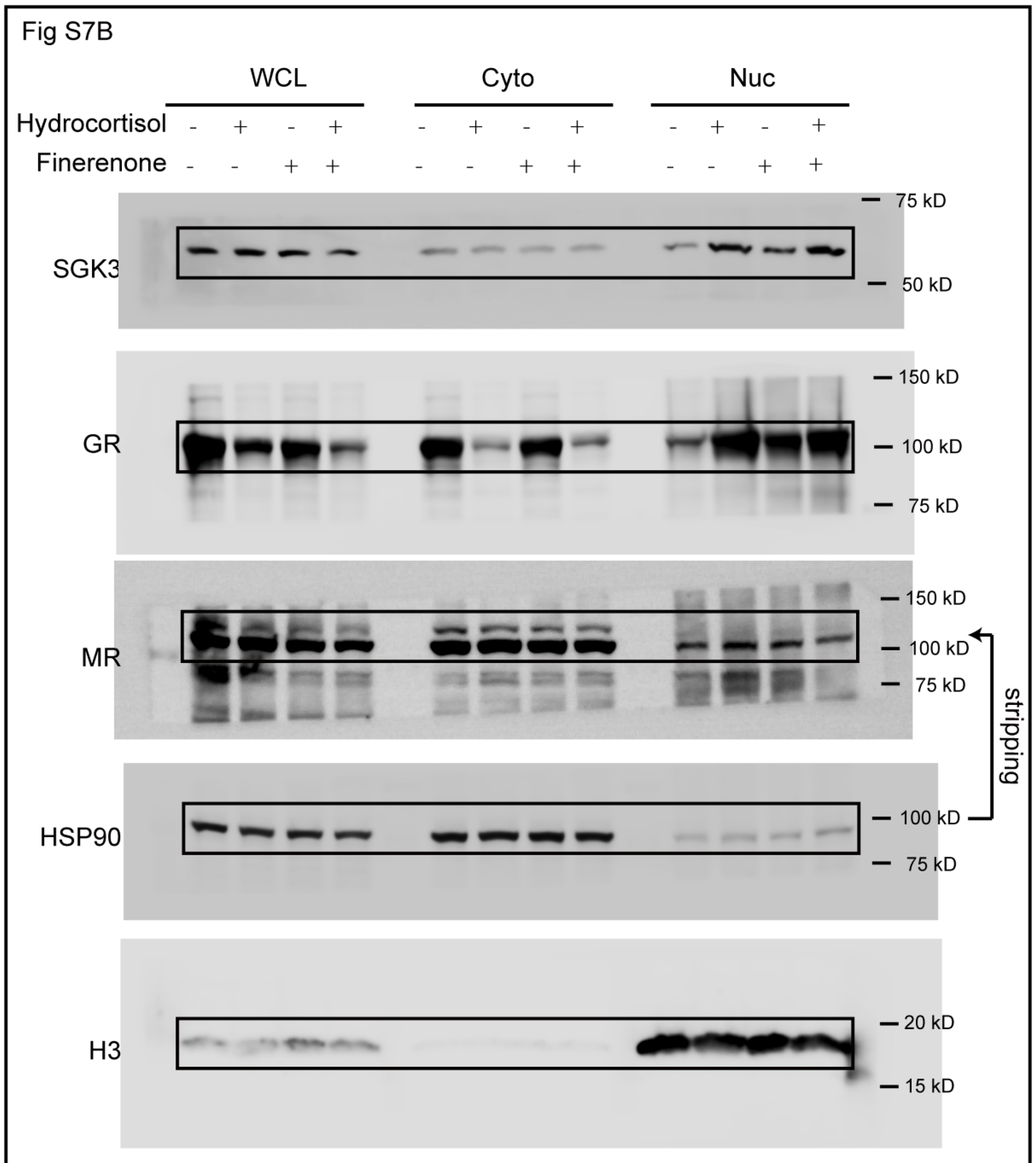

Identical sample aliquots were loaded across multiple gels.
HSP90 (serving as cytosol marker), MR and SGK3 were immunoblotted from a single gel, whereas H3 (serving as nucleus marker) were analyzed on parallel gels.

Full unedited blot for Figure S8

Fig S8A

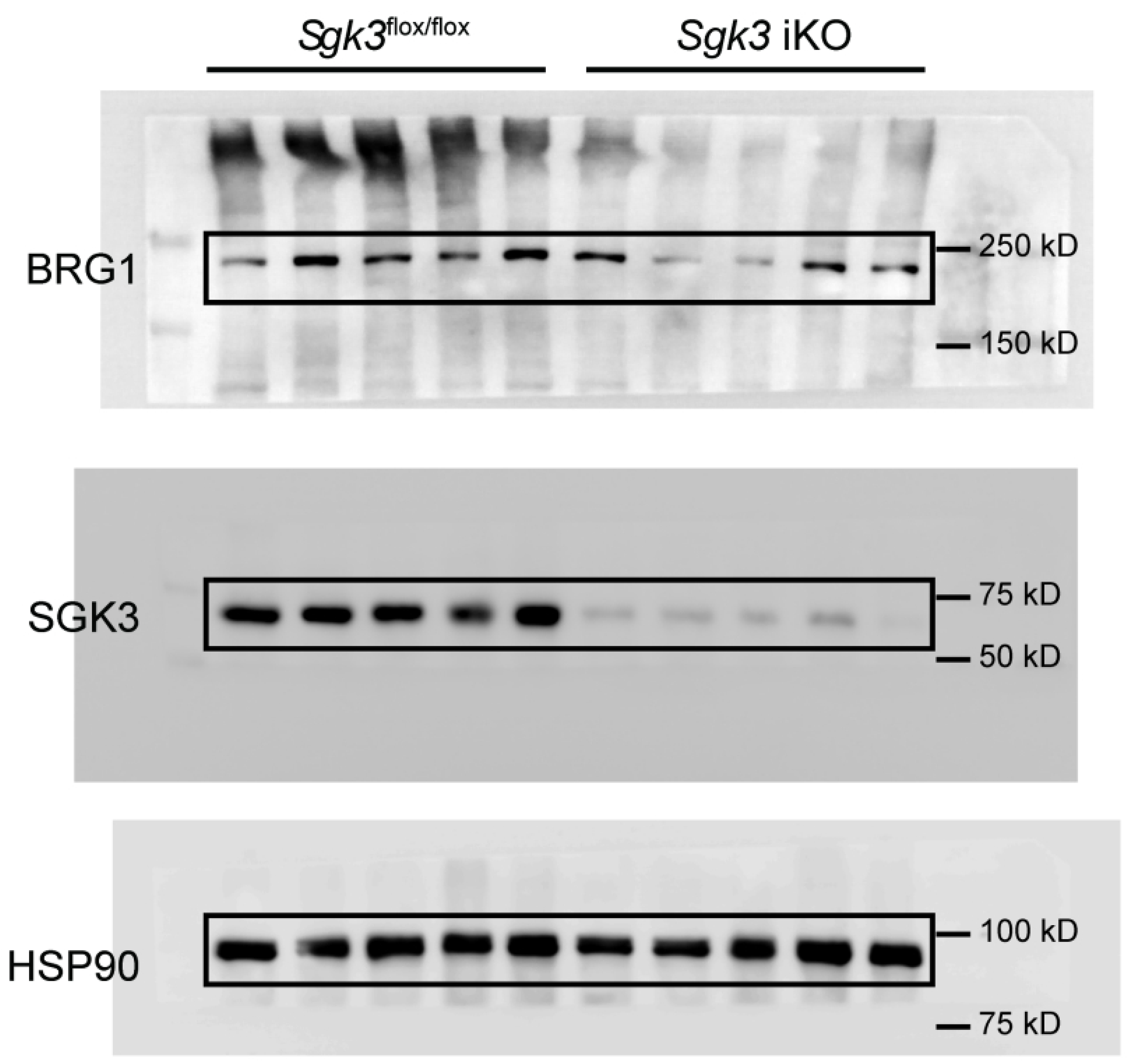

Fig S8B

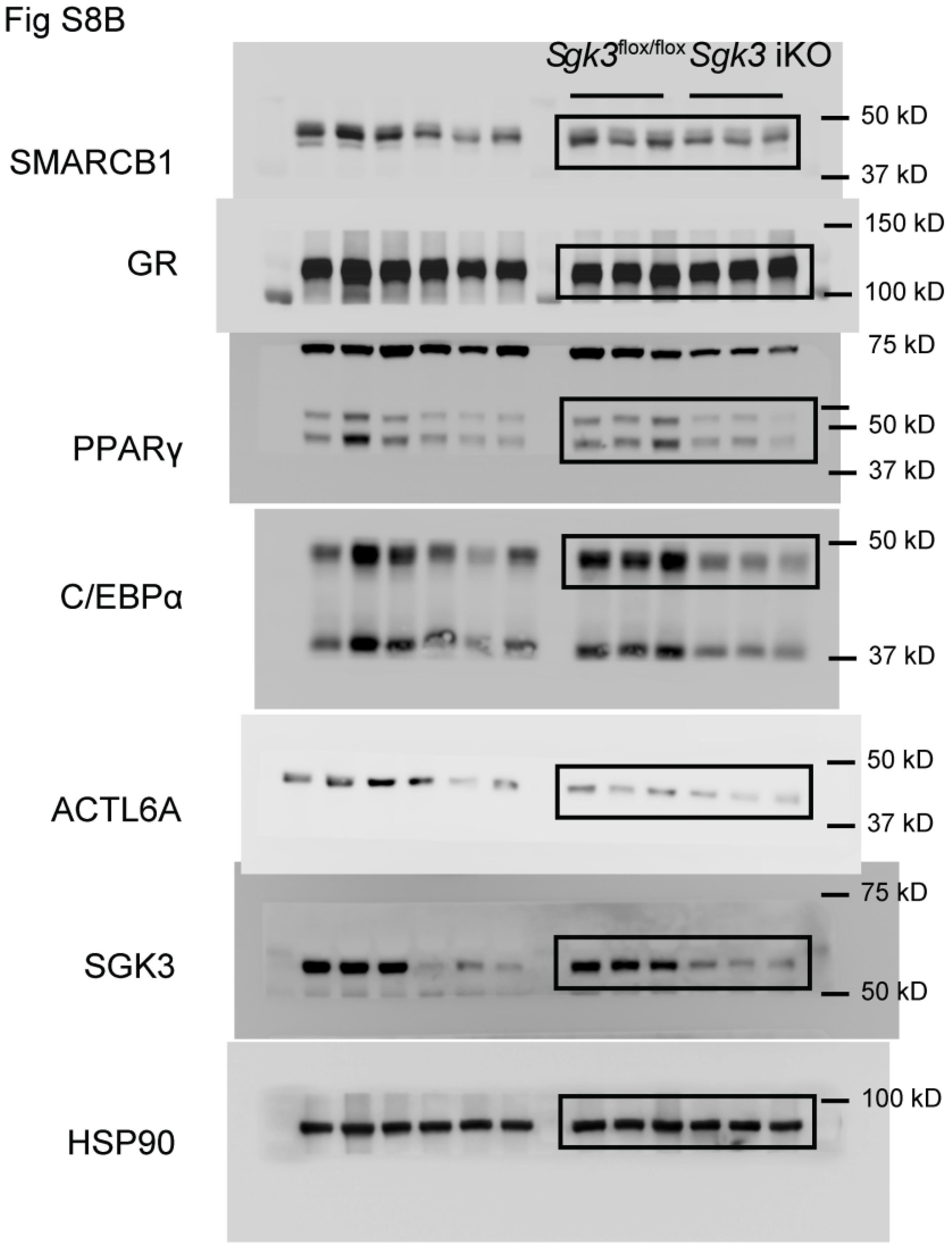

Identical sample aliquots were loaded across multiple gels.
HSP90 (serving as loading control), ACTL6A and SGK3 were immunoblotted from a single gel, whereas others were analyzed on parallel gels (no additional loading control detected).

Fig S8C

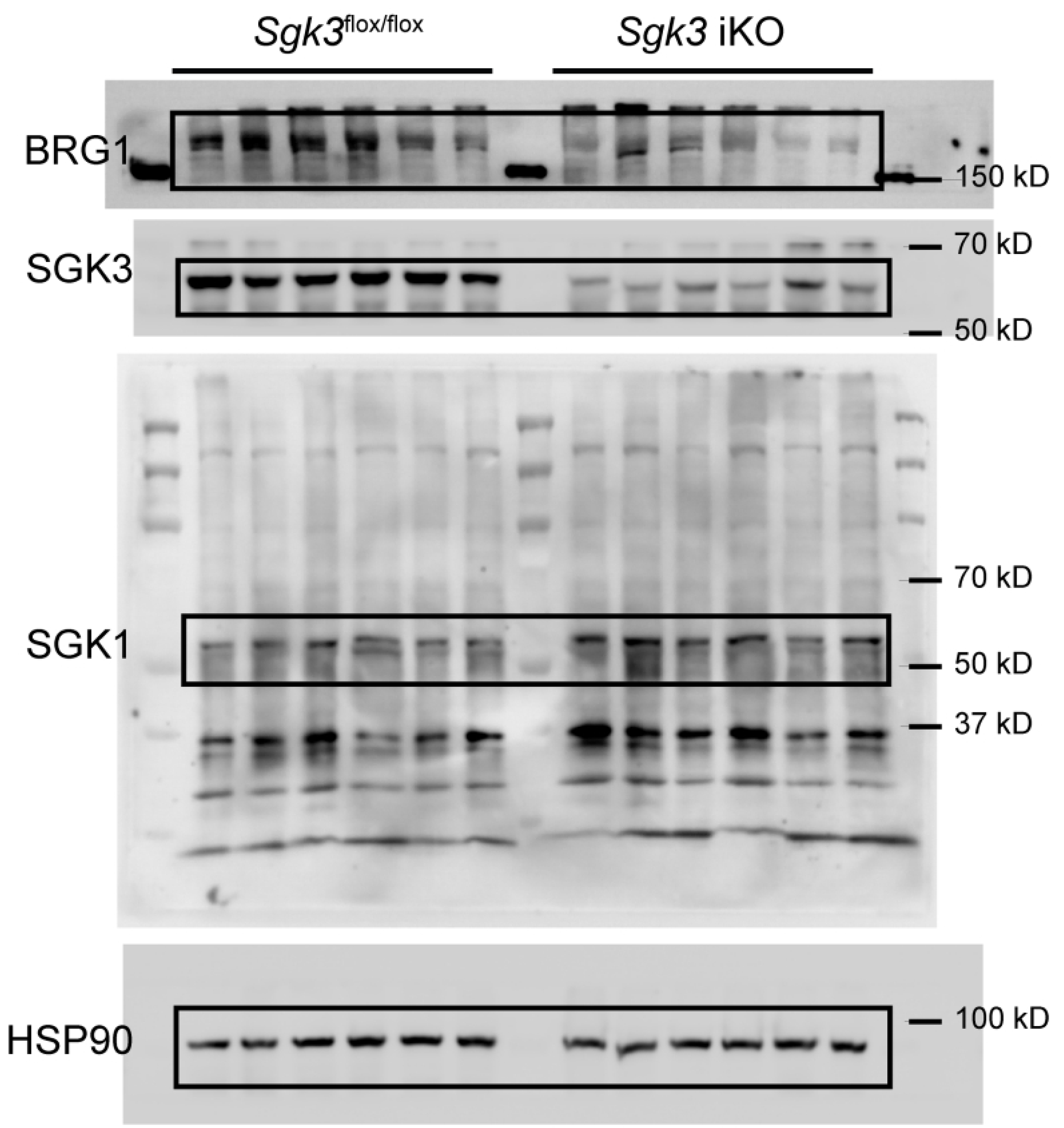

Identical sample aliquots were loaded across multiple gels.
HSP90 (serving as loading control), BRG1 and SGK3 were immunoblotted from a single gel, whereas SGK1 was analyzed on a parallel gel (no additional loading control detected).

Fig S8D

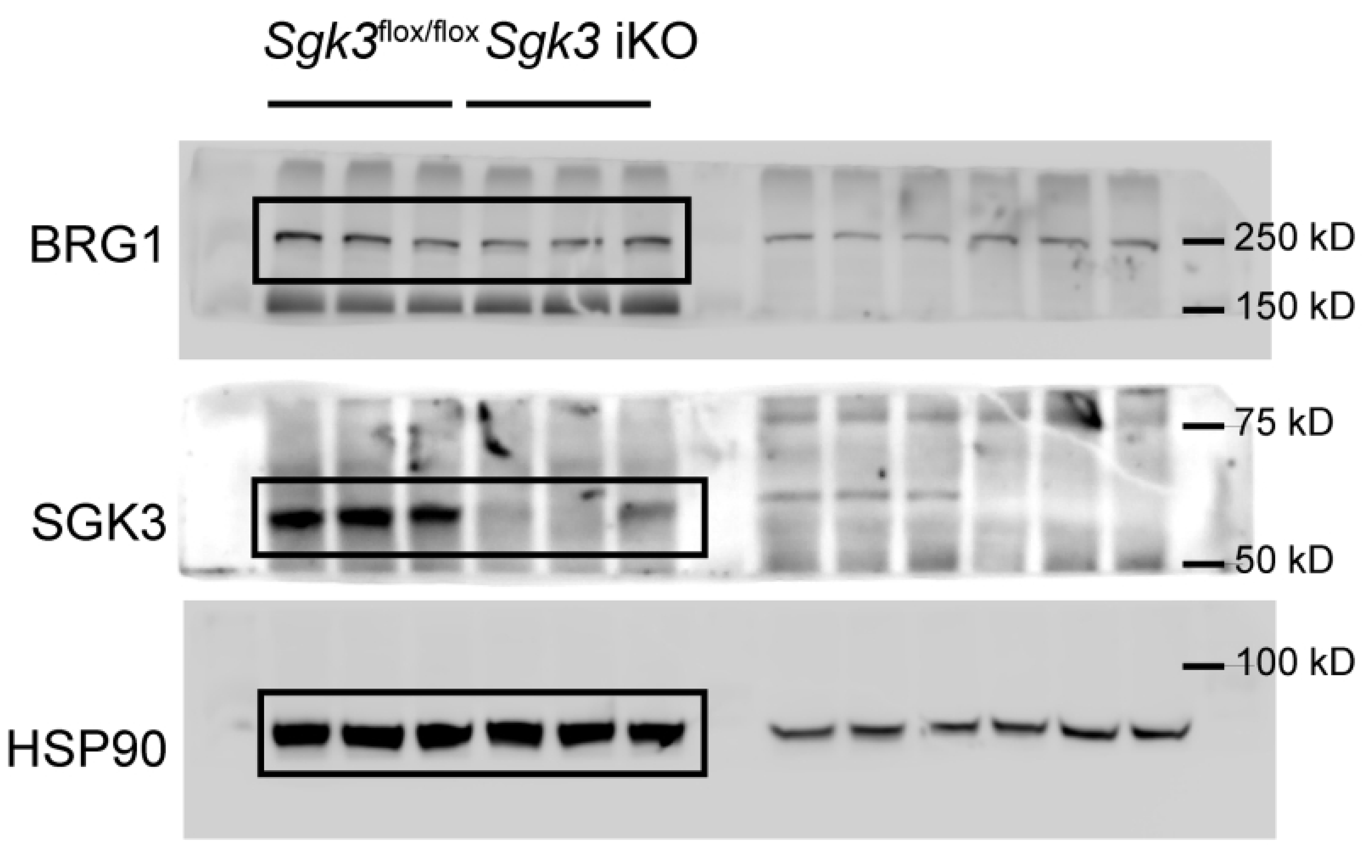

Fig S8E

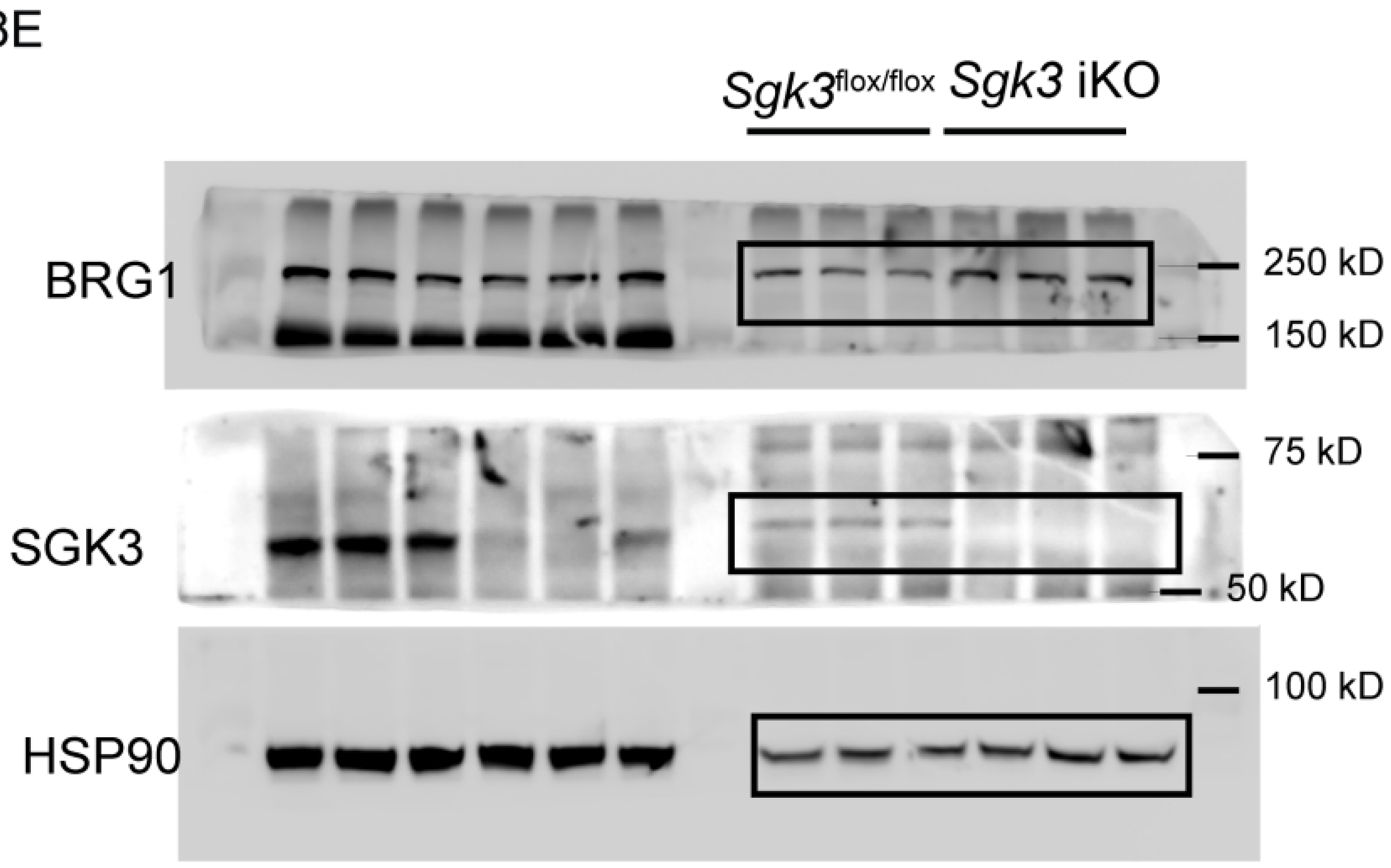

Full unedited blot for Figure S9

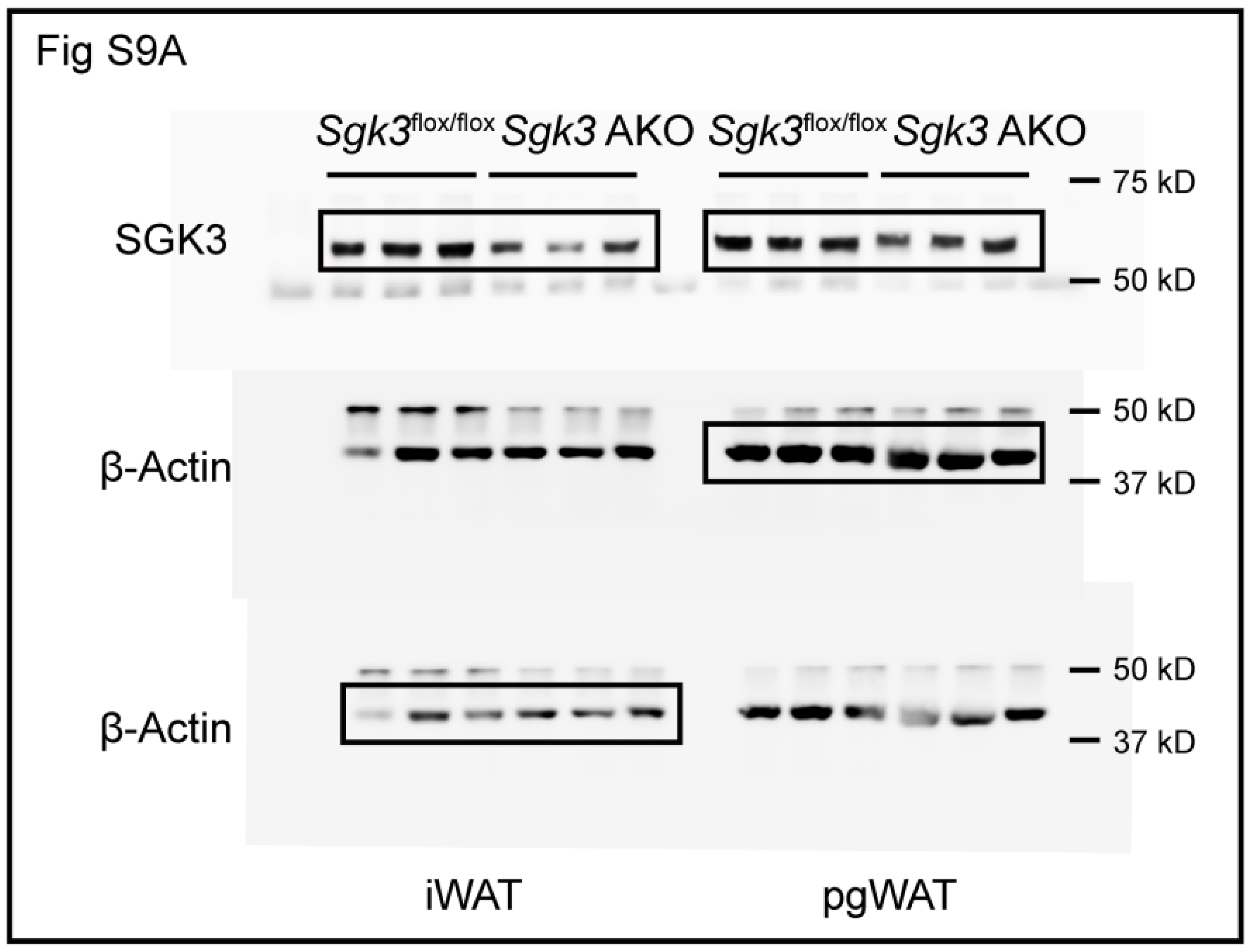

Full unedited blot for Figure S10

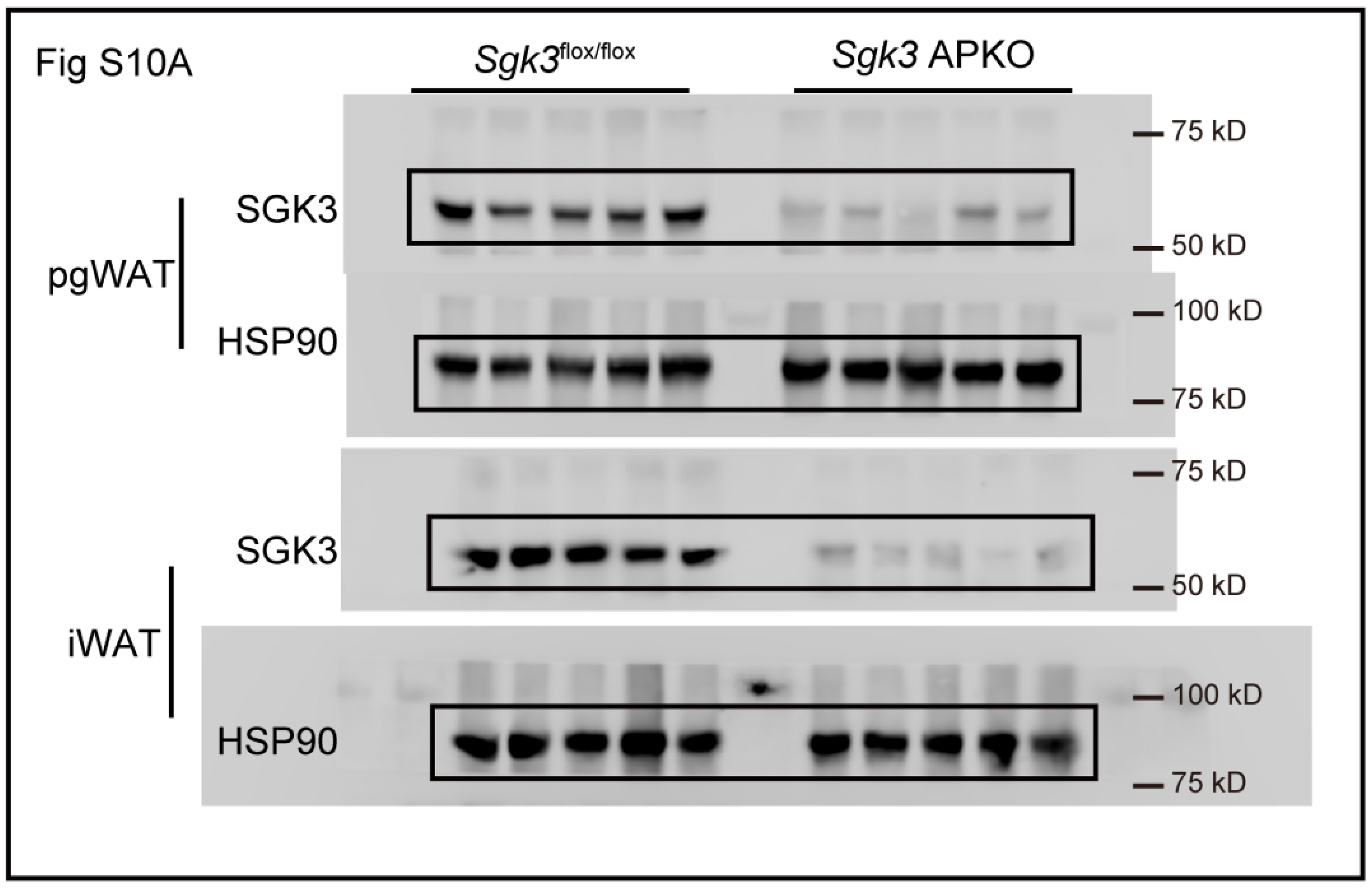

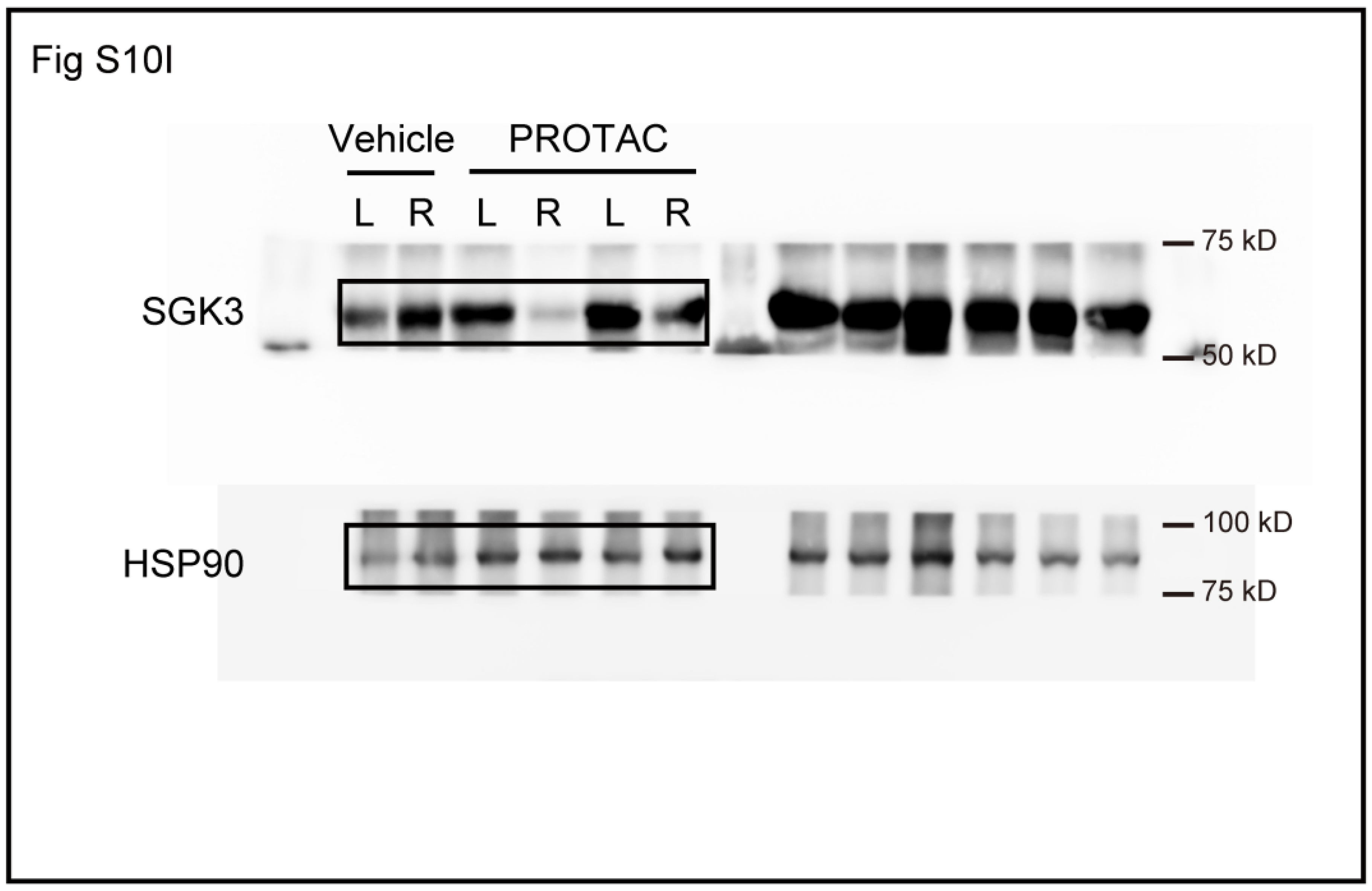

Supplement: Unedited blot and gel images [file jci-135-186534-s038.pdf]
